# Comparative risk of post-acute sequelae following SARS-CoV-2 or influenza virus infection: A retrospective cohort study among United States adults

**DOI:** 10.1371/journal.pmed.1004777

**Published:** 2025-10-09

**Authors:** Joseph A. Lewnard, Debbie E. Malden, Vennis Hong, Jessica Skela, Leora R. Feldstein, Sharon Saydah, Iris Anne C. Reyes, Rulin Hechter, Lina S. Sy, Bradley K. Ackerson, Sara Y. Tartof

**Affiliations:** 1 School of Public Health, University of California, Berkeley, California, United States of America; 2 Kaiser Permanente Southern California Department of Research & Evaluation, Pasadena, California, United States of America; 3 Centers for Disease Control & Prevention, Atlanta, Georgia, United States of America; 4 Department of Health Systems Science, Kaiser Permanente Bernard J. Tyson School of Medicine, Pasadena, California, United States of America; University of Glasgow, UNITED KINGDOM OF GREAT BRITAIN AND NORTHERN IRELAND

## Abstract

**Background:**

Post-acute sequelae (PAS) of SARS-CoV-2 infection are well documented. However, it remains unclear whether such long-term health effects are unique to COVID-19, or also occur following other viral respiratory infections.

**Methods and findings:**

We undertook a retrospective cohort study of 74,738 COVID-19 cases and 18,790 influenza cases within the Kaiser Permanente Southern California healthcare system diagnosed between 1 September, 2022 and 31 December, 2023. Cases received care for index infections across a spectrum of clinical settings, spanning virtual (*n = *35,835; 38.3%), ambulatory (*n* = 26,579; 28.4%), emergency department (*n = *23,388; 25.0%) and inpatient (*n* = 7,726; 8.3%) facilities. We compared 180-day risk of PAS-related healthcare utilization among COVID-19 cases and influenza via adjusted hazard ratios (aHRs) weighted to account for cases’ index infection type and follow-up retention. Adjustment models addressed patients’ demographic characteristics, comorbidity profiles, prior healthcare utilization patterns, and index episode severity. Risk of PAS diagnoses in any clinical setting was only modestly higher among COVID-19 cases in comparison to influenza cases within 31−90 days after cases’ initial illness (aHR = 1.04 [95% confidence interval: 0.99, 1.09]; risk difference = 0.6 [–0.1, 1.2] cases per 100 person-months). This difference was attenuated by 91−180 days (aHR = 1.01 [0.97, 1.06]; risk difference = 0.4 [–0.1, 0.9] cases per 100 person-months). However, COVID-19 cases faced higher risk of severe PAS conditions requiring hospitalization (aHR = 1.31 [1.07, 1.59] and 1.24 [1.03, 1.49] within 31−90 and 91−180 days, respectively). This excess risk of severe PAS was concentrated among COVID-19 cases hospitalized during acute-phase illness, and was attenuated among cases who received antiviral treatment, who had up-to-date vaccination status prior to infection, or who did not require inpatient admission for acute-phase illness. As a limitation, analyses included only PAS resulting in healthcare utilization; patient-reported symptoms and quality-of-life measures were not captured.

**Conclusions:**

In this large, real-world cohort, individuals with non-severe acute respiratory illness caused by SARS-CoV-2 experienced only modestly greater risk of PAS in comparison to those whose illness was caused by influenza. However, COVID-19 cases hospitalized for their initial illness experienced greater risk of severe PAS necessitating inpatient care, and this difference persisted through 180 days of follow-up. Our findings challenge assumptions about the uniqueness of post-acute COVID-19 morbidity and suggest the long-term burden of influenza may be underrecognized.

## Author summary

**Why was this study done?:**

- It is known that people may suffer medical complications long after COVID-19, which are called post-acute sequelae (PAS).
- Similar sequelae are also known to happen after people are infected with influenza or other respiratory viruses.
- It is unclear whether PAS are a unique or more prominent feature of COVID-19 in comparison to influenza.

**What did the researchers find?:**

- Risk of PAS was only modestly higher (4%) after COVID-19 in comparison to influenza.
- By 91−180 days after infection, risk of PAS was similar for COVID-19 and influenza cases.
- Differences in PAS risk were driven by the fact that people with severe COVID-19 tended to have greater risk of severe PAS afterwards.

**What do these findings mean?:**

- PAS risk is similar among most COVID-19 and influenza cases, although people hospitalized for COVID-19 may be at particular risk.
- While PAS is well understood to occur after COVID-19, the burden of PAS associated with influenza may have been historically overlooked.
- As a key limitation of the study, outcomes encompass only PAS necessitating healthcare utilization. Patient-reported symptoms and quality-of-life measures are also important outcomes when measuring PAS.

## Introduction

Post-acute sequelae (PAS) of COVID-19 comprise a complex constellation of symptoms which may involve multiple organ systems. Longitudinal studies have estimated that as many as 30% of individuals may experience at least one post-acute symptom or PAS condition ≥30 days after SARS-CoV-2 infection [1–5], with some patients experiencing persistent PAS up to 3 years following their initial illness [6]. Conditions defining PAS are diverse and have encompassed cardiovascular, respiratory, renal, musculoskeletal, gastrointestinal, endocrine, and neurologic disorders, along with mental health and substance use-related conditions [7]. These outcomes include both exacerbations of pre-existing conditions and new-onset syndromes among individuals without related comorbid conditions prior to the precipitating SARS-CoV-2 infection [8]. Differences in both risk and persistence of PAS after COVID-19 have been reported by sex, by presence of pre-existing comorbidities, and by characteristics of individuals’ initial illness, including multisymptomatic presentation and severity [9–11]. Relatedly, factors preventing progression to severe COVID-19 during the acute stage, including history of COVID-19 vaccination [12–14] and receipt of antivirals during initial illness [15,16], have been reported to lessen individuals’ risk of PAS.

Whether PAS have similar prominence in disease caused by other respiratory viruses is uncertain. Consistent with observations in COVID-19, exacerbations of ischemic heart disease, congestive heart failure, and cerebrovascular disease are known to occur after influenza virus infection [17]. Neurological conditions [18,19]—including Guillain–Barré syndrome [20] and narcolepsy [21]—have also been reported as sequelae of influenza, along with chronic fatigue [22], rhabdomyolysis [23], and renal [24] and hepatic [25] conditions. Case reports have documented both new-onset diabetes associated with severe influenza [26] and exacerbations of existing disease following infection [27]. While awareness of this post-acute disease burden associated with influenza infection dates to the 1918 pandemic [28], it remains unclear whether SARS-CoV-2 and influenza infections pose comparable risk for PAS, and whether the clinical phenotypes of PAS associated with SARS-CoV-2 and influenza are distinct.

Several comparative studies [29–34] have reported greater risk of PAS after COVID-19 in comparison to influenza, albeit inconsistently [35,36]. However, a limitation of some studies has been their restriction to hospitalized COVID-19 and influenza patients, as PAS also affect patients whose preceding viral infections were not severe [37]. Persons hospitalized for COVID-19 and influenza are not representative of all people who acquire infection, and may experience different PAS outcomes in comparison to non-hospitalized patients. We therefore assessed PAS-associated healthcare utilization among a large cohort of individuals receiving care for laboratory-confirmed COVID-19 and influenza (“cases”) whose acute illnesses and subsequent PAS were managed across a spectrum of clinical settings, spanning virtual to inpatient care. We quantified differences in risk of PAS according to both infecting virus and severity of acute-phase illness.

## Methods

### Setting

We analyzed data collected from members of Kaiser Permanente Southern California (KPSC), an integrated healthcare organization comprised of 16 hospitals and 226 medical offices providing care to >4.8 million members across Southern California. Members of KPSC health plans are enrolled through employer-provided, pre-paid, and publicly subsidized insurance plans; broadly, members reflect the socioeconomic and racial and ethnic diversity of the area’s population, have similar prevalence of chronic comorbid conditions, and reside in communities with socioeconomic characteristics mirroring the service area at large [38,39]. All aspects of clinical care received at KPSC facilities, including diagnoses, provider notes, laboratory tests, vaccinations, and prescriptions, are linked by a unique identifier in patients’ electronic health records (EHRs). Insurance claims submitted for reimbursement capture care received from external healthcare providers, enabling near-complete capture of healthcare receipt among members.

### Study design

We conducted an observational cohort study of all KPSC members aged ≥18 years with a positive molecular test for SARS-CoV-2 or influenza between September 1, 2022 and December 31, 2023. We restricted analyses to individuals with accompanying acute respiratory illness (ARI) diagnoses in any clinical setting between 7 days before or after the positive test, and defined index dates as the date of the first ARI diagnosis. This study is reported as per the Strengthening the Reporting of Observational Studies in Epidemiology (STROBE) guideline (S1 Checklist). While the study was retrospective in nature, the design and variable definitions followed a pre-specified protocol (S2 File).

Eligible participants had ≥1 year of continuous KPSC membership (allowing 45-day enrollment gap) before the positive test date to enable accurate capture of comorbid conditions and healthcare utilization. We limited analyses to the first documented ARI diagnosis associated with a positive SARS-CoV-2 or influenza test result in any 180-day period for each individual (index ARI episode). Multiple episodes were included from the same individual if these episodes occurred ≥180 days apart, and each studied episode could be associated with either SARS-CoV-2 or influenza. We excluded SARS-CoV-2 infections identified between 30 days before and 14 days after individuals received any COVID-19 vaccine dose, and influenza infections identified between 30 days before and 14 days after individuals received any influenza vaccine, so that participants’ recorded vaccination status at time of infection reflected doses from which they could be expected to have mounted a response. All individuals meeting eligibility criteria were included in analyses. As our study was retrospective in nature, there was no prespecified sample size or enrollment target.

### Exposures

The primary exposure was the virus detected at each index ARI episode; we defined exposure groups as COVID-19 cases and influenza cases. We further stratified exposure groups based on respiratory viral season (October, 2022–September, 2023, and October–December, 2023) and by viral lineages (influenza type A or B, where available from testing data, and putative SARS-CoV-2 variant based on dominant lineages circulating at the time of individuals’ index date [40]). Additionally, we distinguished index episodes according to the highest-acuity care setting in which individuals received ARI diagnoses during the acute stage of their illness (through 14 days after the index date). In order from lowest to highest acuity, strata included virtual (online/telehealth) settings, ambulatory (ambulatory/urgent care) settings, emergency departments, and inpatient (hospital) facilities.

### Outcomes

We defined PAS as diagnoses occurring within distinct proximal (31−90 days) and distal (91−180 days) risk periods after cases’ index date. We used prespecified International Classification of Diseases, 10th edition, Clinical Modification (ICD-10) diagnoses codes categorized into 10 disease categories with similar mechanisms and body systems affected (cardiopulmonary, hemolytic, respiratory, musculoskeletal, renal, gastrointestinal, neurological, skin, endocrine, and mental health conditions; S1 Table). Outcomes encompassed both new-onset PAS and exacerbations of pre-existing conditions resulting in post-acute healthcare utilization. Although there is no universal case definition for PAS, the study outcome encompassed all diagnosis codes included in US Centers for Disease Control (CDC) criteria for Post-COVID Conditions [2], augmented with definitions from other EHR-based studies [8,41–43]. This approach facilitates alignment with prior studies [2,16] and ongoing surveillance [44] employing CDC’s EHR-based case definition. The list of codes used by CDC to define PAS is purposefully broad to avoid missing conditions potentially occurring as PAS, and prior studies have demonstrated the included codes occur over twice as often among COVID-19 cases in the year following infection as among matched controls not known to have experienced SARS-CoV-2 infection [2]. We conducted separate analyses for PAS within each disease category, and for a composite outcome of any PAS. Consistent with our classification for index ARI diagnoses, we took the highest-acuity care setting where PAS diagnoses were assigned in each follow-up period as an indicator of severity.

We censored observations at death, disenrollment, end of study period, or ARI associated with a distinct virus, whichever occurred first.

### Statistical analysis

We quantified differences in PAS risk between COVID-19 cases versus influenza cases via the adjusted hazard ratio (aHR) for time to first PAS diagnosis. We used a two-stage weighting approach to mitigate selection bias (due to differential depletion of susceptibles prior to follow-up initiation) and confounding [45–47]. First, to address differential censoring prior to the beginning of each follow-up interval among COVID-19 cases and influenza cases, we generated inverse probability of censoring weights (IPCWs) addressing each individual’s probability of remaining enrolled through the beginning of each 30-day period after index [48]. We estimated IPCWs via the Breslow estimator for survival curves from fitted Cox proportional hazards models with death or disenrollment 0–180 days after cases’ index date as the outcome. We fit these models including data from all individuals meeting eligibility criteria for analyses, including those censored before the post-acute follow-up period. Second, to account for differences in characteristics of COVID-19 cases and influenza cases, we also generated inverse probability of treatment (exposure) weights (IPTWs), addressing each individual’s probability of an index infection with SARS-CoV-2 or influenza. We computed stabilized IPTWs via logistic regression models defining infecting virus as the outcome [49]. All weighting models accounted for pre-specified covariates including individuals’ age group, sex, highest-acuity care setting for the index ARI episode, race/ethnicity, Charlson comorbidity index, history of diagnoses in each PAS disease category, history of depression, cigarette smoking, prior-year healthcare utilization (across ambulatory, emergency department, and inpatient settings), body mass index, COVID-19 and seasonal influenza vaccination status, receipt of antiviral treatment for the index ARI episode (as described below), calendar month of the index episode, and neighborhood deprivation index [50]. We included Charlson comorbidity index as a quantitative variable representing individuals’ overall health status due to collinearity of individual Charlson conditions with individuals’ history of diagnoses within the PAS disease categories. We categorized continuous variables (according to pre-specified breakpoints listed in Table 1) to avoid estimation of extreme weights in IPTW and IPCW models. We estimated risk differences for each outcome by subtracting weighted incidence rate estimates among influenza cases from weighted incidence rate estimates among COVID-19 cases.

**Table 1:** Characteristics of COVID-19 cases and influenza cases.

Characteristic		Index infection	
		*COVID-19*	*Influenza*
		*N* = 74,738	*N* = 18,790
Age			
	18–29 years	7,081 (9.5)	4,012 (21.4)
	30–39 years	10,206 (13.7)	3,786 (20.1)
	40–49 years	11,433 (15.3)	3,040 (16.2)
	50–59 years	13,815 (18.5)	2,769 (14.7)
	60–69 years	13,114 (17.5)	2,422 (12.9)
	70–79 years	11,195 (15.0)	1,765 (9.4)
	80–89 years	6,265 (8.4)	808 (4.3)
	≥90 years	1,629 (2.2)	188 (1.0)
		*Median (interquartile range)*	
		56 (41–70)	44 (31–61)
Sex			
	Female	44,689 (59.8)	11,216 (59.7)
	Male	30,049 (40.2)	7,574 (40.3)
Race/ethnicity			
	White, non-Hispanic	19,284 (25.8)	4,483 (23.9)
	Black, non-Hispanic	8,030 (10.7)	1,818 (9.7)
	Hispanic, any race	35,094 (47.0)	10,013 (53.3)
	Asian	8,865 (11.9)	1,553 (8.3)
	Pacific Islander	601 (0.8)	126 (0.7)
	Native American, Alaska Native	147 (0.2)	40 (0.2)
	Multiple/Other/Unknown	2,717 (3.6)	757 (4.0)
Neighborhood deprivation index			
	1st quintile (most deprived)	15,249 (20.4)	3,513 (18.7)
	2nd quintile	15,077 (20.2)	3,712 (19.8)
	3rd quintile	14,967 (20.0)	3,690 (19.6)
	4th quintile	14,885 (19.9)	3,758 (20.0)
	5th quintile (least deprived)	14,529 (19.4)	4,110 (21.9)
Body mass index			
	Underweight	1,016 (1.4)	199 (1.1)
	Normal weight	14,572 (19.5)	3,301 (17.6)
	Overweight	21,002 (28.1)	4,862 (25.9)
	Obese	23,169 (31.0)	5,874 (31.3)
	Morbidly obese	6,257 (8.4)	1,567 (8.3)
Charlson comorbidity index			
	0	36,285 (48.5)	10,992 (58.5)
	1–2	22,347 (29.9)	5,128 (27.3)
	3–5	10,199 (13.6)	1,742 (9.3)
	≥6	5,907 (7.9)	928 (4.9)
		*Median (interquartile range)*	
		1 (0–2)	0 (0–1)
Cigarette smoking			
	Never smoked	48,327 (64.7)	11,971 (63.7)
	Former smoking	16,594 (22.2)	3,324 (17.7)
	Current smoking	3,042 (4.1)	981 (5.2)
History of depression			
	No depression diagnoses	62,177 (83.2)	15,966 (85.0)
	Any depression diagnosis	12,561 (16.8)	2,824 (15.0)
Prior-year ambulatory healthcare utilization			
	0 visits	3,250 (4.3)	1,460 (7.8)
	1–9 visits	42,079 (56.3)	11,779 (62.7)
	10–19 visits	18,015 (24.1)	3,575 (19.0)
	20–29 visits	6,511 (8.7)	1,168 (6.2)
	≥30 visits	4,883 (6.5)	808 (4.3)
		*Median (interquartile range)*	
		8 (4–15)	6 (3–12)
Prior year emergency department utilization			
	No presentation	51,841 (69.4)	13,368 (71.1)
	Any presentation	22,897 (30.6)	5,422 (28.9)
Prior year inpatient admission			
	No admission	67,171 (89.9)	17,480 (93.0)
	Any admission	7,567 (10.1)	1,310 (7.0)
COVID-19 vaccination (doses received)			
	0 doses received	7,350 (9.8)	3,020 (16.1)
	1–2 doses received	15,116 (20.2)	5,497 (29.3)
	≥3 doses received	52,272 (69.9)	10,273 (54.7)
COVID-19 vaccination (recency of last dose)			
	No COVID-19 vaccination within 30–180 days	63,897 (85.5)	17,557 (93.4)
	Received 31–90 days previously	4,281 (5.7)	680 (3.6)
	Received 91–180 days previously	6,560 (8.8)	553 (2.9)
Seasonal influenza vaccination prior to index date			
	Not received in current season	31,013 (41.5)	11,242 (59.8)
	Received in current season	43,725 (58.5)	7,548 (40.2)
Antiviral treatment within 7 days of index date			
	Not received	52,808 (70.7)	8,809 (46.9)
	Received	21,930 (29.3)	9,981 (53.1)
History of previous diagnosis within each PAS category			
	Cardiopulmonary	34,871 (46.7)	6,418 (34.2)
	Hemolytic	7,399 (9.9)	1,121 (6.0)
	Respiratory	32,975 (44.1)	8,289 (44.1)
	Musculoskeletal	32,786 (43.9)	6,843 (36.4)
	Renal	9,896 (13.2)	1,553 (8.3)
	Gastrointestinal	17,820 (23.8)	3,466 (18.4)
	Neurological	22,164 (29.7)	4,406 (23.4)
	Skin	9,910 (13.3)	2,215 (11.8)
	Endocrine	18,701 (25.0)	3,353 (17.8)
	Mental health	25,426 (34.0)	5,549 (29.5)
Highest-acuity care setting for index infection			
	Virtual	32,788 (43.9)	3,047 (16.2)
	Ambulatory	18,017 (24.1)	8,562 (45.6)
	Emergency department	17,253 (23.1)	6,135 (32.7)
	Inpatient	6,680 (8.9)	1,046 (5.6)

PAS: Post-acute sequelae.

Eligible individuals were Kaiser Permanente Southern California healthcare plan members aged ≥18 years with a positive molecular test for SARS-CoV-2 or influenza between September 1, 2022 and December 31, 2023, who had been members of KPSC for ≥12 months preceding their index episode. We restricted analyses to individuals with accompanying acute respiratory illness diagnoses in any clinical setting between 7 days before or after the positive test.

We estimated aHRs using Cox proportional hazards models, defining distinct observations for each 30-day period (31–60, 61–90, 91–120, 121–150, 151–180 days) after the index date to update IPCWs. Weights for each follow-up interval were the product of the individual’s (time-varying) IPCW and the individual’s (time-invariant) IPTW for infection with the identified virus [51]. We generated doubly-robust aHR estimates by including covariates used in the weighting models in analysis models, and used the sandwich variance estimator to account for repeated observations across multiple periods for each individual. We fit separate models for follow-up periods 31–90 days and 91–180 days after index. We verified the proportional hazards assumption within each follow-up period by testing for non-zero slopes of Schoenfeld residuals from fitted Cox proportional hazards models [52]. As an unplanned sensitivity analysis, we verified our estimates in analyses defining alternative interval breaks for continuous covariates, and defining these covariates with a continuous linear term, to verify findings were not sensitive to categorization schemes for continuous variables (S2 Table).

We conducted subgroup analyses restricted to individuals with or without history of prior diagnoses corresponding to PAS conditions within each organ system category in the preceding year. Among persons with such history, we interpreted PAS outcomes as exacerbations of pre-existing conditions, such that aHR estimates conveyed the differences in risk of post-viral exacerbations among COVID-19 cases and influenza cases. Among persons without history of such diagnoses, we interpreted PAS outcomes as new-onset illness, such that aHR estimates conveyed the differential risk of new-onset PAS among COVID-19 cases and influenza cases.

Last, to explore whether other factors modified the relationship between infecting virus and PAS risk, we conducted subgroup analyses stratified by age, sex, severity of the index ARI episode, and receipt of antivirals and vaccines. We defined antiviral receipt for the index infection as the dispensing of molnupiravir or nirmatrelvir–ritonavir (for COVID-19 cases) and oseltamivir, zanamivir, peramivir, or baloxavir (for influenza cases), occurring within 7 days before or after index. We defined up-to-date influenza vaccination status as receipt of seasonal influenza vaccination within 15–180 days before index. Based on US vaccination recommendations during the study period [53] and estimated durations of protection against SARS-CoV-2 infection after vaccination [54–56], we defined up-to-date COVID-19 vaccination as receipt of ≥3 COVID-19 vaccine doses, including one dose within 15–180 days before index.

We conducted analyses using R (version 4.5.1; R Foundation for Statistical Computing, Vienna, Austria, 2025). We fit Cox proportional hazards models using the survival package [57].

### Ethics

This study was reviewed and approved by the Kaiser Permanente Southern California Institutional Review Board with a waiver of informed consent, and was conducted consistent with federal law and CDC policy (45 Code of Federal Regulations [CFR] part 46.101(c); 21 CFR part 56).

## Results

**Fig 1:**
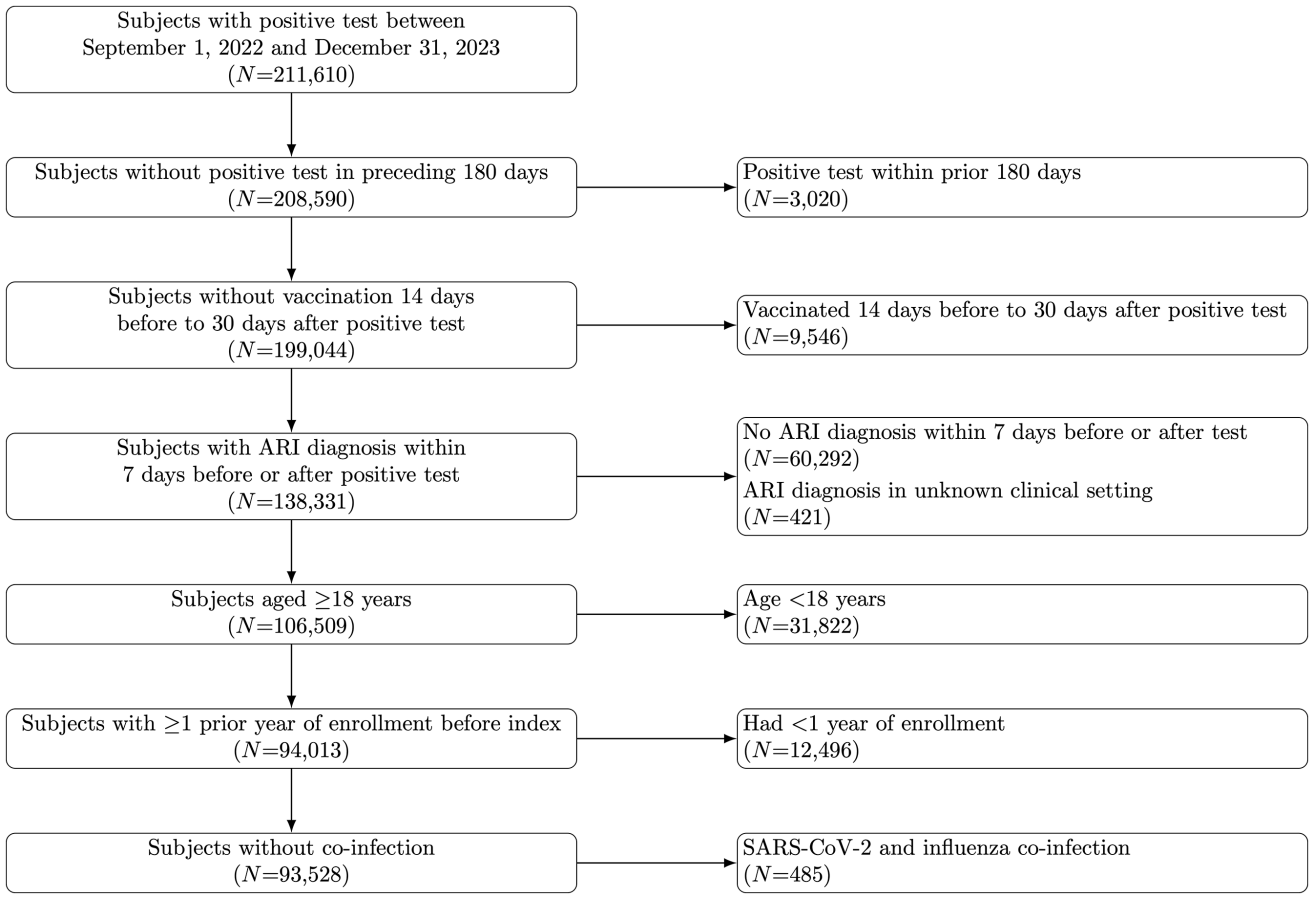
Study flowchart. Enumeration of individuals retained and excluded after applying each eligibility criterion. ARI: acute respiratory illness.

Analyses included 74,738 eligible COVID-19 cases and 18,790 eligible influenza cases (Fig 1). In comparison to influenza cases, COVID-19 cases were older, had higher Charlson comorbidity index scores, and had higher ambulatory healthcare utilization in the year prior to index date as well as greater likelihood of hospital admission in the prior year (Tables 1 and S3). Additionally, COVID-19 cases had higher likelihood of receiving care in either inpatient settings or in virtual settings only for their initial infection.

Overall, 97.3% and 95.3% of COVID-19 cases were retained in follow-up through 31 and 91 days after their index date, respectively (72,745 and 71,238 of 74,738, respectively), as were 97.9% and 95.7% of influenza cases (18,395 and 17,990 of 18,790, respectively; S4 Table). A total of 826 COVID-19 cases (1.1%) and 89 influenza cases (0.5%) died within 30 days after their index dates, while 1,391 COVID-19 cases (1.9%) and 149 influenza cases (0.8%) died within 90 days. Within 31−90 days after index dates, 45.2% of COVID-19 cases and 38.9% of influenza cases received PAS diagnoses, and within 91−180 days after index dates, 52.8% of COVID-19 cases and 44.0% of influenza cases received PAS diagnoses. Cardiopulmonary PAS were the most common outcome among both COVID-19 cases and influenza cases.

Absolute differences in weighted incidence of PAS diagnoses between COVID-19 cases and influenza cases were modest in analyses adjusting for individuals’ probability of infection with each virus and censoring (Table 2). Within 31–90 days after index, weighted incidence rates of PAS diagnoses per 100 person-months at risk were 26.5 (95% confidence interval: 26, 2–26.8) among COVID-19 cases and 25.9 (25.4, 26.5) among influenza cases. Within 91–180 days after index, weighted incidence rates were 23.0 (22.7, 23.2) and 22.6 (22.1, 23.0) per 100 person-months among COVID-19 and influenza cases, respectively. Risk differences totaled 0.6 (–0.1, 1.2) additional COVID-19 cases experiencing PAS per 100 person-months within 31–90 days after index, and 0.4 (–0.1, 0.9) additional COVID-19 cases experiencing PAS per 100 person-months within 91–180 days after index. We observed 1.5 (1.4, 1.5) and 1.2 (1.1, 1.4) inpatient PAS diagnoses per 100 person-months among COVID-19 and influenza cases, respectively, 31–90 days after index, and 1.1 (1.0, 1.1) and 0.9 (0.8, 1.0) inpatient PAS diagnoses per 100 person-months among COVID-19 and influenza cases, respectively, 91–180 days after index. Risk differences corresponded to 0.2 (0.1, 0.4) and 0.1 (0.0, 0.2) additional COVID-19 cases experiencing PAS requiring inpatient admission per 100 person-months within 31–90 and 91–180 days after index, respectively. These differences increased with severity of cases’ initial ARI (Table 3). Among cases whose initial ARI necessitated inpatient care, risk differences corresponded to 3.2 (2.2, 4.1) and 1.5 (0.8, 2.2) additional COVID-19 cases receiving PAS diagnoses in inpatient settings per 100 person-months within 31–90 and 91–180 days after index, respectively.

**Table 2:** Risk of post-acute sequelae, by disease category and severity.

PAS outcome	Weighted cumulative incidence over period per 100 person-months at risk (95% CI), by index infection^a^					
	*Within 31–90 days after index*			*Within 91–180 days after index*		
	COVID-19	Influenza	Risk difference	COVID-19	Influenza	Risk difference
*PAS diagnosed in any setting, by disease category*						
Cardiopulmonary	10.4 (10.3, 10.6)	10.5 (10.2, 10.9)	–0.1 (–0.5, 0.3)	8.5 (8.4, 8.7)	8.2 (8.0, 8.5)	0.3 (0.0, 0.6)
Hemolytic	1.6 (1.5, 1.7)	1.6 (1.4, 1.7)	0.0 (–0.1, 0.2)	1.2 (1.2, 1.3)	1.1 (1.0, 1.2)	0.1 (0.0, 0.2)
Respiratory	7.3 (7.2, 7.5)	8.1 (7.8, 8.4)	–0.7 (–1.1, –0.4)	6.0 (5.9, 6.1)	6.3 (6.1, 6.6)	–0.3 (–0.6, –0.1)
Musculoskeletal	6.2 (6.1, 6.4)	6.3 (6.0, 6.5)	0.0 (–0.3, 0.3)	5.7 (5.6, 5.8)	5.5 (5.3, 5.7)	0.2 (0.0, 0.4)
Renal	3.1 (3.0, 3.2)	3.4 (3.2, 3.6)	–0.3 (–0.6, –0.1)	2.4 (2.3, 2.5)	2.5 (2.4, 2.6)	–0.1 (–0.3, 0.1)
Gastrointestinal	3.3 (3.2, 3.4)	2.9 (2.7, 3.1)	0.4 (0.2, 0.6)	2.7 (2.7, 2.8)	2.5 (2.4, 2.6)	0.2 (0.1, 0.4)
Neurological	4.5 (4.4, 4.7)	4.2 (4.0, 4.4)	0.3 (0.1, 0.6)	4.0 (4.0, 4.1)	3.4 (3.2, 3.6)	0.7 (0.5, 0.8)
Skin	1.2 (1.2, 1.3)	1.3 (1.2, 1.4)	–0.1 (–0.2, 0.0)	1.2 (1.2, 1.3)	1.3 (1.2, 1.4)	–0.1 (–0.2, 0.0)
Endocrine	6.8 (6.6, 6.9)	6.4 (6.1, 6.6)	0.4 (0.1, 0.7)	5.4 (5.3, 5.5)	5.3 (5.1, 5.5)	0.1 (–0.2, 0.3)
Mental health	6.7 (6.6, 6.9)	7.0 (6.7, 7.3)	–0.3 (–0.6, 0.0)	5.5 (5.4, 5.7)	5.2 (5.0, 5.4)	0.3 (0.1, 0.5)
*PAS, by severity*						
PAS diagnosed in any setting	26.5 (26.2, 26.8)	25.9 (25.4, 26.5)	0.6 (–0.1, 1.2)	23.0 (22.7, 23.2)	22.6 (22.1, 23.0)	0.4 (–0.1, 0.9)
PAS diagnosed in ambulatory or higher-acuity setting	20.9 (20.6, 21.1)	20.2 (19.7, 20.7)	0.7 (0.1, 1.2)	18.4 (18.2, 18.6)	18.3 (17.9, 18.7)	0.2 (–0.3, 0.6)
PAS diagnosed in emergency department or higher-acuity setting	6.6 (6.5, 6.7)	6.5 (6.2, 6.8)	0.1 (–0.2, 0.4)	5.9 (5.8, 6.0)	5.6 (5.4, 5.8)	0.3 (0.0, 0.5)
PAS diagnosed in inpatient setting	1.5 (1.4, 1.5)	1.2 (1.1, 1.4)	0.2 (0.1, 0.4)	1.1 (1.0, 1.1)	0.9 (0.8, 1.0)	0.1 (0.0, 0.2)

PAS: post-acute sequelae; CI: Confidence interval.

^a^Incidence is computed accounting for time to first event for the indicated outcomes; person-time after the first event is excluded from denominators. Weights are computed as the product of inverse probability of treatment/exposure weights (accounting for individuals’ probability of infection with their identified virus) and inverse probability of censoring weights (accounting for the probability of retention through each 30-day period after the index date). Weighting models adjusted for individuals’ age group, sex, highest-acuity care setting for the index acute respiratory illness episode, race/ethnicity, Charlson comorbidity index, history of diagnoses in each PAS disease category, history of depression, cigarette smoking, prior-year healthcare utilization (across ambulatory, emergency department, and inpatient settings), body mass index, COVID-19 and seasonal influenza vaccination status, receipt of antiviral treatment for the index episode, calendar month of the index episode, and neighborhood deprivation index. We present unadjusted frequencies of each outcome, stratified by index infection, in S4 Table.

**Table 3:** Risk of PAS diagnoses in inpatient settings according to severity of initial infection.

Population	Weighted cumulative incidence over period per 100 person-months at risk (95% CI), by index infection^a^					
	*Within 31–90 days after index*			*Within 31–90 days after index*		
	COVID-19	Influenza	Risk difference	COVID-19	Influenza	Risk difference
All cases	1.5 (1.4, 1.5)	1.2 (1.1, 1.4)	0.2 (0.1, 0.4)	1.1 (1.0, 1.1)	0.9 (0.8, 1.0)	0.1 (0.0, 0.2)
Cases with ARI diagnoses in ambulatory or higher-acuity settings	2.1 (2.0, 2.2)	1.6 (1.4, 1.7)	0.5 (0.3, 0.7)	1.5 (1.4, 1.6)	1.3 (1.2, 1.4)	0.2 (0.0, 0.3)
Cases with ARI diagnoses in emergency department or higher-acuity settings	3.4 (3.3, 3.6)	2.4 (2.1, 2.7)	1.0 (0.7, 1.3)	2.4 (1.8, 2.2)	2.0 (1.8, 2.2)	0.4 (0.2, 0.7)
Cases with ARI diagnoses in inpatient settings	8.1 (7.6, 8.6)	4.9 (4.2, 5.7)	3.2 (2.2, 4.1)	5.5 (5.1, 5.9)	3.9 (3.4, 4.6)	1.5 (0.8, 2.2)

ARI: Acute respiratory illness; PAS: post-acute sequelae; CI: Confidence interval.

^a^Incidence is computed accounting for time to first event for the indicated outcomes; person-time after the first event is excluded from denominators. Weights are computed as the product of inverse probability of treatment/exposure weights (accounting for individuals’ probability of infection with their identified virus) and inverse probability of censoring weights (accounting for the probability of retention through each 30-day period after the index date). Weighting models adjusted for individuals’ age group, sex, highest-acuity care setting for the index acute respiratory illness episode, race/ethnicity, Charlson comorbidity index, history of diagnoses in each PAS disease category, history of depression, cigarette smoking, prior-year healthcare utilization (across ambulatory, emergency department, and inpatient settings), body mass index, COVID-19 and seasonal influenza vaccination status, receipt of antiviral treatment for the index episode, calendar month of the index episode, and neighborhood deprivation index. We present unadjusted frequencies of each outcome, stratified by index infection, in S4 Table.

Based on estimates from doubly-robust Cox proportional hazards models, COVID-19 cases experienced modestly higher risk of PAS in comparison to influenza cases within 31–90 days after index (aHR = 1.04 [95% confidence interval: 0.99, 1.09]), which were attenuated by 91–180 days after index (aHR = 1.01 [0.97, 1.06]; Table 3). However, COVID-19 cases had 31% (7%, 59%) and 24% (3%, 49%) higher risk than influenza cases of receiving PAS diagnoses in inpatient settings 31–90 days and 91–180 days after index in comparison to influenza cases, respectively. Findings were consistent in sensitivity analyses with alternative handling of continuous covariates in weighting and adjustment models (S5 Table).

Point estimates favored higher risk of inpatient PAS diagnoses among COVID-19 cases versus influenza cases in the 31−90 day follow-up period across all categories of PAS diagnoses, spanning 1.23 (0.93, 1.61) for renal PAS to 7.27 (1.55, 34.09) for skin-associated PAS (Table 4). Although likewise elevated across all disease categories in the 91−180 day follow-up period, point estimates were lower in this period relative to the 31−90 day period for all categories except musculoskeletal PAS (aHR = 2.04 [1.29, 3.24]) and neurological PAS (aHR = 2.11 [1.54, 2.90]). For each syndromic category, the association of COVID-19 with increased risk of PAS was weaker for diagnoses within virtual, ambulatory, and emergency department settings than for PAS diagnoses within inpatient settings.

**Table 4:** Adjusted hazard ratios of post-acute sequelae.

Outcome	Acuity of care setting for PAS diagnoses	Adjusted hazard ratio (95% CI), COVID-19 cases compared to influenza cases^a^	
		*Within 31–90 days after index*	*Within 91–180 days after index*
Any PAS			
	Any setting	1.04 (0.99, 1.09)	1.01 (0.97, 1.06)
	Ambulatory or higher-acuity setting	1.06 (1.00, 1.12)	1.00 (0.95, 1.06)
	Emergency department or higher-acuity setting	1.05 (0.96, 1.16)	1.07 (0.98, 1.17)
	Inpatient setting	1.31 (1.07, 1.59)	1.24 (1.03, 1.49)
Cardiopulmonary PAS			
	Any setting	1.06 (0.98, 1.13)	1.06 (0.98, 1.14)
	Ambulatory or higher-acuity setting	1.05 (0.97, 1.13)	1.08 (0.99, 1.17)
	Emergency department or higher-acuity setting	1.13 (1.00, 1.28)	1.12 (1.00, 1.26)
	Inpatient setting	1.33 (1.07, 1.67)	1.24 (1.01, 1.53)
Hemolytic PAS			
	Any setting	1.11 (0.94, 1.30)	1.19 (0.98, 1.45)
	Ambulatory or higher-acuity setting	1.11 (0.94, 1.32)	1.15 (0.94, 1.41)
	Emergency department or higher-acuity setting	1.55 (1.16, 2.07)	1.43 (1.10, 1.85)
	Inpatient setting	1.66 (1.09, 2.54)	1.23 (0.90, 1.68)
Respiratory PAS			
	Any setting	0.91 (0.84, 1.00)	0.95 (0.87, 1.04)
	Ambulatory or higher-acuity setting	0.94 (0.85, 1.03)	0.98 (0.89, 1.08)
	Emergency department or higher-acuity setting	1.01 (0.88, 1.15)	1.01 (0.88, 1.16)
	Inpatient setting	1.43 (1.11, 1.85)	1.03 (0.80, 1.34)
Musculoskeletal PAS			
	Any setting	1.00 (0.90, 1.11)	1.04 (0.96, 1.14)
	Ambulatory or higher-acuity setting	1.00 (0.89, 1.12)	1.07 (0.97, 1.17)
	Emergency department or higher-acuity setting	1.02 (0.82, 1.28)	1.10 (0.92, 1.31)
	Inpatient setting	1.40 (0.83, 2.37)	2.04 (1.29, 3.24)
Renal PAS			
	Any setting	0.96 (0.84, 1.09)	0.96 (0.82, 1.12)
	Ambulatory or higher-acuity setting	0.92 (0.80, 1.05)	0.97 (0.81, 1.15)
	Emergency department or higher-acuity setting	0.98 (0.78, 1.24)	1.13 (0.93, 1.37)
	Inpatient setting	1.23 (0.93, 1.61)	1.17 (0.89, 1.52)
Gastrointestinal PAS			
	Any setting	1.19 (1.07, 1.33)	1.12 (0.99, 1.27)
	Ambulatory or higher-acuity setting	1.19 (1.06, 1.35)	1.15 (1.00, 1.33)
	Emergency department or higher-acuity setting	1.35 (1.11, 1.64)	1.35 (1.10, 1.65)
	Inpatient setting	1.75 (1.28, 2.38)	1.43 (1.05, 1.94)
Neurological PAS			
	Any setting	1.12 (1.00, 1.26)	1.23 (1.11, 1.37)
	Ambulatory or higher-acuity setting	1.09 (0.97, 1.23)	1.17 (0.97, 1.41)
	Emergency department or higher-acuity setting	1.21 (0.97, 1.51)	1.17 (0.97, 1.41)
	Inpatient setting	1.60 (1.15, 2.23)	2.11 (1.54, 2.90)
Skin PAS			
	Any setting	0.93 (0.75, 1.15)	0.91 (0.73, 1.13)
	Ambulatory or higher-acuity setting	1.01 (0.81, 1.25)	0.87 (0.68, 1.11)
	Emergency department or higher-acuity setting	1.54 (1.07, 2.22)	0.96 (0.61, 1.51)
	Inpatient setting	7.27 (1.55, 34.09)	2.82 (0.85, 9.32)
Endocrine PAS			
	Any setting	1.10 (1.01, 1.20)	1.02 (0.93, 1.11)
	Ambulatory or higher-acuity setting	1.10 (1.00, 1.20)	1.01 (0.92, 1.12)
	Emergency department or higher-acuity setting	1.15 (0.98, 1.34)	1.12 (0.96, 1.32)
	Inpatient setting	1.36 (1.04, 1.77)	1.13 (0.87, 1.47)
Mental health PAS			
	Any setting	0.98 (0.90, 1.07)	1.09 (0.99, 1.19)
	Ambulatory or higher-acuity setting	1.06 (0.94, 1.19)	1.13 (1.00, 1.27)
	Emergency department or higher-acuity setting	1.20 (1.00, 1.44)	1.15 (0.95, 1.39)
	Inpatient setting	1.58 (1.20, 2.08)	1.40 (1.06, 1.84)

PAS: post-acute sequelae; CI: Confidence interval.

^a^Estimates are computed as adjusted hazards ratios via doubly-robust Cox proportional hazards models weighted to account for individuals’ inverse probability of infection with their identified virus and the inverse of their probability of retention through each 30-day period after the index date. Covariates used in weighting models are included in the analysis model. We use the sandwich variance estimator to account for repeated observations of individuals across multiple 30-day periods. Weighting models adjusted for individuals’ age group, sex, highest-acuity care setting for the index acute respiratory illness episode, race/ethnicity, Charlson comorbidity index, history of diagnoses in each PAS disease category, history of depression, cigarette smoking, prior-year healthcare utilization (across ambulatory, emergency department, and inpatient settings), body mass index, COVID-19 and seasonal influenza vaccination status, receipt of antiviral treatment for the index episode, calendar month of the index episode, and neighborhood deprivation index. We present unadjusted frequencies of each outcome, stratified by index infection, in S4 Table.

Differences between COVID-19 cases and influenza cases in risk of post-acute exacerbations of pre-existing conditions were also mainly evident for PAS diagnoses in inpatient settings (Table 5). Within 31−90 days after index, point estimates spanned 19%–122% higher risk for inpatient PAS exacerbations of pre-existing conditions across all syndromic categories. Within 91−180 days after index, point estimates were attenuated across all categories except musculoskeletal and neurological conditions (aHR = 3.16 [1.76, 5.69] and 2.61 [1.84, 3.71], respectively). Similarly, point estimates favored greater risk of new-onset PAS conditions necessitating inpatient care among COVID-19 cases, with aHR point estimates spanning 20%–373% greater risk within 31−90 days after cases’ index dates across all disease categories. Within 91−180 days after index, renal PAS (aHR = 2.31 [1.26, 4.24]) and skin-associated PAS (aHR = 3.25 [0.71, 14.85]) showed the greatest elevation among COVID-19 cases in comparison to influenza cases for diagnoses in inpatient settings. Generally, aHR estimates were associated with greater statistical uncertainty for comparisons of new-onset PAS diagnoses than for PAS exacerbations, reflecting the low incidence of new-onset PAS diagnoses compared to PAS exacerbations among both COVID-19 cases and influenza cases (S6 Table).

**Table 5:** Adjusted hazards ratios of post-acute sequelae as new-onset conditions or exacerbations of existing conditions.

Outcome	Acuity of care setting for PAS diagnoses	Adjusted hazard ratio (95% CI), COVID-19 cases compared to influenza cases^a^			
		*Exacerbation of pre-existing conditions after index infection* ^b^		*New-onset PAS conditions after index infection* ^c^	
		Within 31–90 days after index	Within 91–180 days after index	Within 31–90 days after index	Within 91–180 days after index
Cardiopulmonary PAS					
	Any setting	1.05 (0.97, 1.13)	1.02 (0.94, 1.10)	1.02 (0.84, 1.25)	1.25 (1.06, 1.47)
	Ambulatory or higher-acuity setting	1.04 (0.96, 1.12)	1.03 (0.95, 1.13)	1.05 (0.86, 1.30)	1.26 (1.07, 1.49)
	Emergency department or higher-acuity setting	1.10 (0.96, 1.25)	1.11 (0.98, 1.25)	1.33 (0.99, 1.79)	1.08 (0.83, 1.42)
	Inpatient setting	1.32 (1.05, 1.65)	1.20 (0.97, 1.50)	1.23 (0.65, 2.35)	1.56 (0.95, 2.55)
Hemolytic PAS					
	Any setting	1.04 (0.86, 1.26)	1.24 (0.96, 1.59)	1.21 (0.86, 1.72)	1.05 (0.78, 1.41)
	Ambulatory or higher-acuity setting	1.06 (0.87, 1.29)	1.19 (0.92, 1.55)	1.19 (0.82, 1.72)	1.01 (0.75, 1.36)
	Emergency department or higher-acuity setting	1.76 (1.22, 2.52)	1.45 (1.03, 2.05)	1.21 (0.77, 1.89)	1.31 (0.92, 1.87)
	Inpatient setting	1.97 (1.14, 3.42)	1.19 (0.80, 1.78)	1.27 (0.69, 2.35)	1.24 (0.81, 1.90)
Respiratory PAS					
	Any setting	0.96 (0.87, 1.06)	0.96 (0.87, 1.06)	0.84 (0.71, 0.99)	0.96 (0.80, 1.15)
	Ambulatory or higher-acuity setting	1.00 (0.90, 1.12)	0.96 (0.86, 1.07)	0.84 (0.69, 1.01)	1.07 (0.88, 1.31)
	Emergency department or higher-acuity setting	1.07 (0.91, 1.25)	0.99 (0.85, 1.15)	0.90 (0.71, 1.15)	1.07 (0.80, 1.42)
	Inpatient setting	1.33 (0.99, 1.77)	1.09 (0.82, 1.47)	2.12 (1.30, 3.44)	0.77 (0.46, 1.30)
Musculoskeletal PAS					
	Any setting	0.97 (0.85, 1.10)	1.01 (0.90, 1.13)	1.07 (0.90, 1.27)	1.13 (0.97, 1.31)
	Ambulatory or higher-acuity setting	0.97 (0.84, 1.11)	1.05 (0.93, 1.18)	1.08 (0.89, 1.30)	1.11 (0.95, 1.31)
	Emergency department or higher-acuity setting	0.90 (0.68, 1.21)	1.04 (0.82, 1.31)	1.32 (1.05, 1.66)	1.23 (0.97, 1.56)
	Inpatient setting	1.33 (0.72, 2.44)	3.16 (1.76, 5.69)	1.60 (0.50, 5.12)	0.91 (0.42, 1.96)
Renal PAS					
	Any setting	0.95 (0.83, 1.08)	0.95 (0.81, 1.11)	1.09 (0.68, 1.74)	1.18 (0.86, 1.62)
	Ambulatory or higher-acuity setting	0.92 (0.80, 1.05)	0.95 (0.80, 1.12)	1.02 (0.63, 1.65)	1.28 (0.91, 1.79)
	Emergency department or higher-acuity setting	0.95 (0.75, 1.21)	1.11 (0.90, 1.37)	1.31 (0.76, 2.25)	1.28 (0.88, 1.85)
	Inpatient setting	1.20 (0.90, 1.59)	1.06 (0.79, 1.43)	1.51 (0.71, 3.21)	2.31 (1.26, 4.24)
Gastrointestinal PAS					
	Any setting	1.15 (1.00, 1.32)	1.22 (1.05, 1.42)	1.29 (1.09, 1.52)	0.99 (0.79, 1.24)
	Ambulatory or higher-acuity setting	1.15 (0.99, 1.34)	1.24 (1.05, 1.47)	1.28 (1.06, 1.54)	1.01 (0.78, 1.31)
	Emergency department or higher-acuity setting	1.46 (1.14, 1.86)	1.50 (1.16, 1.94)	1.12 (0.82, 1.54)	1.13 (0.81, 1.57)
	Inpatient setting	1.88 (1.32, 2.67)	1.57 (1.06, 2.34)	1.41 (0.77, 2.57)	1.17 (0.74, 1.87)
Neurological PAS					
	Any setting	1.11 (0.97, 1.29)	1.20 (1.04, 1.37)	1.14 (0.95, 1.36)	1.32 (1.14, 1.52)
	Ambulatory or higher-acuity setting	1.09 (0.94, 1.28)	1.19 (1.02, 1.38)	1.08 (0.89, 1.31)	1.31 (1.12, 1.53)
	Emergency department or higher-acuity setting	1.17 (0.88, 1.56)	1.16 (0.90, 1.48)	1.25 (0.93, 1.68)	1.18 (0.92, 1.51)
	Inpatient setting	1.68 (1.20, 2.35)	2.61 (1.84, 3.71)	1.32 (0.54, 3.20)	1.17 (0.64, 2.13)
Skin PAS					
	Any setting	0.88 (0.66, 1.17)	1.12 (0.86, 1.47)	0.98 (0.75, 1.29)	0.82 (0.62, 1.09)
	Ambulatory or higher-acuity setting	0.88 (0.64, 1.19)	1.11 (0.82, 1.50)	1.14 (0.89, 1.45)	0.78 (0.57, 1.06)
	Emergency department or higher-acuity setting	2.22 (0.81, 6.05)	1.77 (0.84, 3.71)	1.36 (0.95, 1.95)	0.80 (0.47, 1.35)
	Inpatient setting	– –	– –	4.73 (0.99, 22.69)	3.25 (0.71, 14.85)
Endocrine PAS					
	Any setting	1.11 (1.02, 1.22)	1.01 (0.92, 1.11)	1.17 (0.82, 1.66)	1.29 (0.95, 1.74)
	Ambulatory or higher-acuity setting	1.10 (1.00, 1.21)	1.01 (0.91, 1.12)	1.22 (0.81, 1.83)	1.29 (0.92, 1.82)
	Emergency department or higher-acuity setting	1.15 (0.98, 1.34)	1.11 (0.94, 1.30)	1.45 (0.69, 3.08)	1.38 (0.80, 2.36)
	Inpatient setting	1.33 (1.02, 1.74)	1.10 (0.84, 1.45)	1.88 (0.36, 9.79)	1.20 (0.50, 2.87)
Mental health PAS					
	Any setting	1.00 (0.91, 1.10)	1.06 (0.96, 1.17)	0.90 (0.72, 1.12)	1.16 (0.98, 1.36)
	Ambulatory or higher-acuity setting	1.13 (1.00, 1.29)	1.11 (0.97, 1.28)	0.84 (0.64, 1.11)	1.13 (0.91, 1.40)
	Emergency department or higher-acuity setting	1.19 (0.98, 1.46)	1.17 (0.95, 1.45)	1.20 (0.76, 1.89)	0.99 (0.65, 1.50)
	Inpatient setting	1.57 (1.17, 2.11)	1.41 (1.03, 1.93)	1.59 (0.80, 3.16)	1.24 (0.72, 2.13)

PAS: post-acute sequelae; CI: Confidence interval.

^a^Estimates are computed as adjusted hazards ratios via doubly-robust Cox proportional hazards models weighted to account for individuals’ inverse probability of infection with their identified virus and the inverse of their probability of retention through each 30-day period after the index date. Covariates used in weighting models are included in the analysis model. We use the sandwich variance estimator to account for repeated observations of individuals across multiple 30-day periods. Weighting models adjusted for individuals’ age group, sex, highest-acuity care setting for the index acute respiratory illness episode, race/ethnicity, Charlson comorbidity index, history of diagnoses in each PAS disease category, history of depression, cigarette smoking, prior-year healthcare utilization (across ambulatory, emergency department, and inpatient settings), body mass index, COVID-19 and seasonal influenza vaccination status, receipt of antiviral treatment for the index episode, calendar month of the index episode, and neighborhood deprivation index. We present unadjusted frequencies of each outcome, stratified by index infection, in S4 Table.

^b^Analyses of exacerbation outcomes are limited to individuals with a history of any preceding diagnosis (prior to their index infection) with conditions included in the indicated PAS category.

^c^Analyses of new-onset PAS outcomes are limited to individuals without a history of any preceding diagnosis (prior to their index infection) with conditions included in the indicated PAS category.

Higher risk of inpatient PAS diagnoses among COVID-19 cases compared to influenza cases was primarily apparent among patients whose index ARI episodes necessitated inpatient admission (Table 6). Compared to influenza cases who received ARI diagnoses in inpatient settings, COVID-19 cases who received ARI diagnoses in inpatient settings had 73% (30%, 130%) and 49% (9%, 106%) higher risk of inpatient PAS diagnoses 31−90 and 91−180 days after index, respectively. Evidence of increased risk of PAS diagnoses among COVID-19 cases was inconsistent among patients whose index ARI episodes were managed in lower-acuity care settings. Differences in risk of inpatient PAS diagnoses among COVID-19 cases and influenza cases were apparent only among cases who did not receive antiviral treatment (aHR = 2.09 [1.45, 3.03] for untreated COVID-19 cases versus untreated influenza cases within 31−90 days after index). We did not identify differences in risk of inpatient PAS diagnoses among COVID-19 cases and influenza cases who were up-to-date with COVID-19 and influenza vaccines, respectively, at the time of their initial illness. Differences in risk of inpatient-diagnosed PAS conditions among COVID-19 cases versus influenza cases were more strongly apparent among female cases than male cases; additionally, differences in PAS risk among COVID-19 cases versus influenza cases appeared within multiple age groups.

**Table 6:** Adjusted hazard ratios of post-acute sequelae across differing subgroups.

Characteristic	Stratum	Adjusted hazards ratio (95% CI), COVID-19 cases compared to influenza cases, according to severity of outcome^a^			
		*Within 31–90 days after index*		*Within 91–180 days after index*	
		PAS of any severity	Inpatient-managed PAS	PAS of any severity	Inpatient-managed PAS
Age group					
	18–29	1.02 (0.90, 1.15)	1.23 (0.78, 1.93)	1.17 (1.04, 1.33)	0.79 (0.49, 1.30)
	30–39	1.07 (0.95, 1.21)	1.08 (0.70, 1.66)	1.01 (0.90, 1.13)	1.50 (0.95, 2.39)
	40–49	0.99 (0.87, 1.12)	0.83 (0.46, 1.53)	1.01 (0.91, 1.12)	0.66 (0.39, 1.10)
	50–59	1.10 (0.96, 1.25)	1.27 (0.68, 2.36)	0.96 (0.85, 1.08)	0.87 (0.51, 1.48)
	60–69	0.99 (0.89, 1.10)	1.04 (0.71, 1.50)	1.00 (0.90, 1.11)	1.56 (1.07, 2.29)
	70–79	1.01 (0.90, 1.13)	1.82 (1.26, 2.63)	0.99 (0.88, 1.12)	1.47 (1.03, 2.08)
	80–89	0.99 (0.85, 1.15)	1.08 (0.71, 1.64)	1.03 (0.89, 1.20)	0.97 (0.65, 1.46)
	≥90	0.90 (0.70, 1.16)	2.25 (1.16, 4.36)	0.88 (0.65, 1.19)	1.09 (0.60, 1.97)
Sex					
	Female	1.03 (0.96, 1.09)	1.47 (1.15, 1.89)	1.01 (0.95, 1.07)	1.31 (1.04, 1.65)
	Male	1.05 (0.97, 1.14)	1.18 (0.89, 1.56)	1.01 (0.93, 1.09)	1.13 (0.87, 1.49)
Antiviral received					
	Not received	1.04 (0.96, 1.12)	2.09 (1.45, 3.03)	1.07 (1.00, 1.14)	1.28 (0.92, 1.78)
	Received	1.05 (0.98, 1.13)	0.76 (0.58, 1.00)	0.99 (0.92, 1.06)	0.96 (0.75, 1.22)
Vaccination status^b^					
	Up-to-date vaccination status with respect to index infection	0.95 (0.73–1.23)	0.85 (0.66–1.09)	0.56 (0.20–1.55)	0.63 (0.29–1.38)
Highest-acuity care setting for index infection					
	Virtual	1.03 (0.92, 1.15)	1.07 (0.59, 1.95)	0.96 (0.86, 1.06)	1.43 (0.82, 2.48)
	Ambulatory	1.10 (1.02, 1.19)	1.06 (0.70, 1.60)	1.02 (0.95, 1.08)	0.92 (0.64, 1.32)
	Emergency department	0.99 (0.92, 1.06)	1.14 (0.84, 1.55)	1.10 (1.02, 1.18)	1.00 (0.76, 1.31)
	Inpatient	0.99 (0.86, 1.14)	1.73 (1.30, 2.30)	0.93 (0.81, 1.06)	1.49 (1.09, 2.06)

PAS: Post-acute sequelae; CI: Confidence interval.

^a^Estimates are computed as adjusted hazards ratios via doubly-robust Cox proportional hazards models weighted to account for individuals’ inverse probability of infection with their identified virus and the inverse of their probability of retention through each 30-day period after the index date. Covariates used in weighting models are included in the analysis model. We use the sandwich variance estimator to account for repeated observations of individuals across multiple 30-day periods. Weighting models adjusted for individuals’ age group, sex, highest-acuity care setting for the index acute respiratory illness episode, race/ethnicity, Charlson comorbidity index, history of diagnoses in each PAS disease category, history of depression, cigarette smoking, prior-year healthcare utilization (across ambulatory, emergency department, and inpatient settings), body mass index, COVID-19 and seasonal influenza vaccination status, receipt of antiviral treatment for the index episode, calendar month of the index episode, and neighborhood deprivation index. We present unadjusted frequencies of each outcome, stratified by index infection, in S4 Table.

^b^We defined up-to-date as: ≥ 3 COVID-19 vaccine doses, with ≥1 dose received in the 6 months preceding index dates, for COVID-19, and ≥1 seasonal influenza vaccine dose within the 6 months preceding index dates for influenza.

Our finding that COVID-19 cases experienced greater risk than influenza cases of severe PAS necessitating hospital admission held within analyses disaggregated by season (2022−23 and 2023−24), SARS-CoV-2 variant period (BA.4/BA.5, XBB/XBB.1.5, and BA.2.86/JN.1), and influenza comparator virus (A or B), although these analyses encountered lower statistical power than primary analyses (S7 Table). Consistent with our primary analyses, we did not identify appreciable differences in risk of PAS outcomes that included diagnoses in lower-acuity settings within most subgroups (Tables 6 and S7).

## Discussion

Within our study, COVID-19 cases experienced only modestly greater risk of PAS diagnoses up to 180 days after their index ARI episode in comparison to influenza cases. The primary distinguishing characteristic of PAS following COVID-19 versus influenza was that COVID-19 cases experienced higher risk of severe PAS necessitating inpatient admission. Further, these differences in risk were dependent on the severity of cases’ index infection. Whereas risk of severe PAS was comparable among COVID-19 cases and influenza cases who received care for their index episode in virtual, ambulatory, or emergency department settings, COVID-19 cases hospitalized for their index infection had higher risk of severe PAS than influenza cases who were hospitalized for their index infection. Differences in PAS risk were attenuated in strata of patients who received antiviral treatment or whose vaccination status was up-to-date against their infecting virus. Our findings demonstrate that although COVID-19 and influenza cases both experience substantial risk of PAS, the spectrum of PAS occurring after COVID-19 includes more severe manifestations than PAS occurring after influenza. This heightened risk of severe PAS is concentrated among patients who experienced severe COVID-19, and may be attenuated by vaccination or antiviral treatment.

Cardiopulmonary manifestations accounted for the greatest share of all PAS outcomes observed among both COVID-19 and influenza cases, and have been widely documented as sequelae of respiratory virus infections, not limited to SARS-CoV-2 and influenza [28–36,58–60]. Prior comparative studies of COVID-19 and influenza have reported greater PAS risk among COVID-19 across nearly all studied disease categories with the exception of respiratory conditions [30], which we likewise found to be more common among influenza cases in analyses that included non-severe PAS outcomes. One prior study which did not identify differences in PAS among hospitalized COVID-19 and influenza cases included further adjustment for disease severity during hospitalization [36]. Differences we report between hospitalized COVID-19 and influenza cases may thus relate to the greater severity of COVID-19 among patients requiring care in inpatient settings, as characterized in previous studies [61].

Among individuals with pre-existing conditions corresponding to each PAS category, COVID-19 cases experienced higher risk than influenza cases of exacerbations leading to inpatient PAS diagnoses. Point estimates of aHRs were consistent with the hypothesis that COVID-19 cases also experienced greater risk of new-onset severe PAS in comparison to influenza cases. However, given the low incidence of severe PAS among individuals without pre-existing conditions, such differences were uncertain from a statistical perspective and may not be clinically meaningful.

Comprehensive capture of healthcare interactions in an integrated system enabled us to represent a broad severity spectrum of index ARI episodes and subsequent PAS in comparison to prior studies, which have primarily addressed hospitalized COVID-19 cases and influenza cases [29,30]. Moreover, access to detailed information on cases’ demographics, comorbidities, and prior patterns of healthcare utilization enabled us to adjust rigorously for differences in characteristics of COVID-19 cases and influenza cases, as well as time-dependent sources of bias not addressed in prior studies.

Limitations of our analyses should also be considered. First, studied PAS outcomes could be more strongly associated with SARS-CoV-2 infection than with influenza infection simply because our PAS code list was assembled in part from findings of prior studies documenting that the studied outcomes occurred at elevated rates among COVID-19 survivors [2,8,41–43]. However, our consideration of a purposefully broad case definition, and our findings of only weak statistical evidence for associations of the studied outcome with COVID-19, together lessen this concern. Gold-standard case definitions for PAS remain to be established, and case definitions based on healthcare utilization recorded in EHR data differ inherently from those based on patient-reported symptoms, physical examination, and laboratory criteria [62,63]. Second, our analysis was limited to adults. Children also experience PAS associated with SARS-CoV-2 [64] and other respiratory viruses [65,66], suggesting similar studies should be undertaken in pediatric cohorts. Third, testing practices for SARS-CoV-2 and influenza differed during the study period. Whereas SARS-CoV-2 testing was widely undertaken for both inpatients and in ambulatory settings, influenza testing was less widely available and may have been reserved for individuals with more severe illness, or those receiving an initial negative SARS-CoV-2 test result. Our analyses adjusted for measured differences between COVID-19 cases and influenza cases; however, bias due to unmeasured variables and other factors may persist under our analytic framework [67]. Fourth, as our study was restricted to individuals who received ARI diagnoses, our findings did not address PAS potentially associated with asymptomatic SARS-CoV-2 and influenza infections. Fifth, our analyses did not address differences in PAS risk beyond 180 days after cases’ index dates, limiting our ability to compare differences in the lengths of time COVID-19 cases and influenza cases may be at risk for PAS. Regardless, attenuated effect estimates by 91−180 days after index suggests longer-term differences are unlikely. Considering only first PAS events over each follow-up interval diminishes our ability to compare the nature of PAS experienced among COVID-19 and influenza cases, including the frequency or persistence of clinical complaints. Sixth, although KPSC members represent the demographic and socioeconomic diversity of the surrounding population, healthcare delivery for KPSC members may not reflect patterns among other providers, impacting external generalizability of our findings. Last, while our study included adjustment and subgroup analyses for individuals receiving common outpatient antiviral therapies, the effects of earlier COVID-19 treatment strategies (including monoclonal antibodies or remdesivir) on PAS risk remain poorly understood.

We found that COVID-19 cases experienced only modestly higher risk of PAS in comparison to influenza cases, although PAS among COVID-19 cases were more likely to require hospital admission than PAS among influenza cases. Whereas PAS are widely known to occur after COVID-19, our results suggest that the risk of PAS associated with influenza may be under-appreciated and worthy of further study. For COVID-19, rigorous estimates of the burden of PAS are necessary for the contemporary context of novel circulating variants and population immunity; such analyses should adjust for counterfactual risk absent infection through control-group comparisons [68]. Aside from influenza, PAS are also known to occur in association with numerous other respiratory viruses [69], including other sarbecoviruses [70], respiratory syncytial virus [71], enteroviruses [72], and Epstein–Barr virus [73]. Improved understanding of this post-acute burden can collectively inform the value of interventions aiming to prevent or mitigate the severity of respiratory virus infections.

### Disclaimer

The findings and conclusions in this report are those of the authors and do not necessarily represent the official position of the Centers for Disease Control and Prevention (CDC). Mention of a product or company name is for identification purposes only and does not constitute endorsement by CDC.

## Supporting information

S1 File Supporting information, including S1 Table (Diagnosis codes (ICD-10-CM) used to define post-acute sequelae); S2 Table (Case counts under alternative categorization of continuous covariates); S3 Table (Individual Charlson comorbidity categories among COVID-19 cases and influenza cases); S4 Table (Cohort retention and frequency of post-acute sequelae managed in any clinical setting); S5 Table (Adjusted hazard ratios of any post-acute sequelae estimated using models with alternative handling of continuous covariates); S6 Table (Cumulative incidence of post-acute sequelae as new-onset conditions or exacerbations of existing conditions, diagnosed in any clinical setting); and S7 Table (Adjusted hazard ratios of post-acute sequelae, subset by infecting virus lineage or period). (PDF)

S2 File Study protocol. (PDF)

S1 Checklist STROBE checklist. (PDF)

S1 Table Diagnosis codes (ICD-10-CM) used to define post-acute sequelae. (PDF)

S2 Table Case counts under alternative categorization of continuous covariates. (PDF)

S3 Table Individual Charlson comorbidity categories among COVID-19 cases and influenza cases. (PDF)

S4 Table Cohort retention and frequency of post-acute sequelae managed in any clinical setting. (PDF)

S5 Table Adjusted hazard ratios of any post-acute sequelae estimated using models with alternative handling of continuous covariates. (PDF)

S6 Table Cumulative incidence of post-acute sequelae as new-onset conditions or exacerbations of existing conditions, diagnosed in any clinical setting. (PDF)

S7 Table Adjusted hazard ratios of post-acute sequelae, subset by infecting virus lineage or period. (PDF)

## Abbreviations

aHR: adjusted hazard ratio
ARI: acute respiratory illness
CDC: Centers for Disease Control
EHR: electronic health record
PAS: post-acute sequelae
