# Supplementary material for: Comparative risk of post-acute sequelae following SARS-CoV-2 or influenza virus infection: A retrospective cohort study among United States adults
Source: PLoS Med. 2025 Oct 9;22(10):e1004777. doi: 10.1371/journal.pmed.1004777 (PMC12551960; doi:10.1371/journal.pmed.1004777)
Supplement: S2 File — (PDF) [file pmed.1004777.s002.pdf]

Kaiser Permanente Southern California (KPSC) Protocol CFA Epidemiological Study: Risk of Long-term Sequelae among Adults with SARS-CoV-2 or Influenza virus infections

Background

Although most patients with COVID-19 recover within a few weeks, some have persistent or recurring symptoms, develop new sequelae, or experience exacerbations of pre-existing chronic conditions after acute-phase SARS-CoV-2 infection. This phenomenon, referred to as post-acute sequelae (PAS), includes signs, symptoms, and conditions that are present after the acute phase of infection (i.e., the first 28 days) and can persist for years [1]. Prior evidence has also suggested that PAS are associated with acute respiratory illnesses caused by other infections, including with influenza viruses [2]. However, the public health burden associated with SARS-CoV-2 infection has appeared considerably greater during early phases of the COVID-19 pandemic. Early studies have estimated that 10-30% of SARS-CoV-2 infections result in PAS [3-6], although differences across studies in design, endpoint definitions, use of comparator populations, and circulating variant makes estimating the true incidence of PAS after SARS-CoV-2 difficult to quantify [7, 8]. Substantial population immunity against SARS-CoV-2 due to infection and vaccination further contributes to difficulty in translating findings from prior studies to the present epidemiologic context.

With endemic co-circulation of SARS-CoV-2 and influenza viruses now occurring, updated understanding of the risk for PAS to occur following infection with SARS-CoV-2 infection and influenza viruses is needed to inform disease burden and preventive interventions. The results will help to inform disease prevention initiatives, including treatment and vaccination recommendations. We will undertake a study comparing risks of PAS following COVID-19 and influenza virus infection among adults aged ≥18 years enrolled in Kaiser Permanente Southern California health plans.

Objective: To determine the relative risk of new onset PAS following ARI with confirmed SARS-CoV-2 or Influenza virus infections among adults.

Study Period: September 2022-April 2024

Deliverables: First interim analyses to be available within 28 days; final analyses to be available within 60 days; unless otherwise agreed

1. Interim analysis complete – around June 30, 2024
2. Final analysis complete – around August 31, 2024

Methods

Overall study design

This will be a retrospective cohort study of all KPSC members aged ≥18 years with a documented positive SARS-CoV-2 or Influenza test between September 1st, 2022 and December 31st, 2023. This is a data-only study. Written participant consent and medical chart reviews are not required.

Data sources

Electronic health records (EHR) data will be used from all KPSC hospitals and medical centers including inpatient, outpatient, and ED settings. External data sources will be used for KP members who received care from external healthcare providers.

Figure 1. Number of positive SARS-CoV-2 tests (per day) and positive Influenza tests (per week) reported to California Department of Public Health, 2022-2024

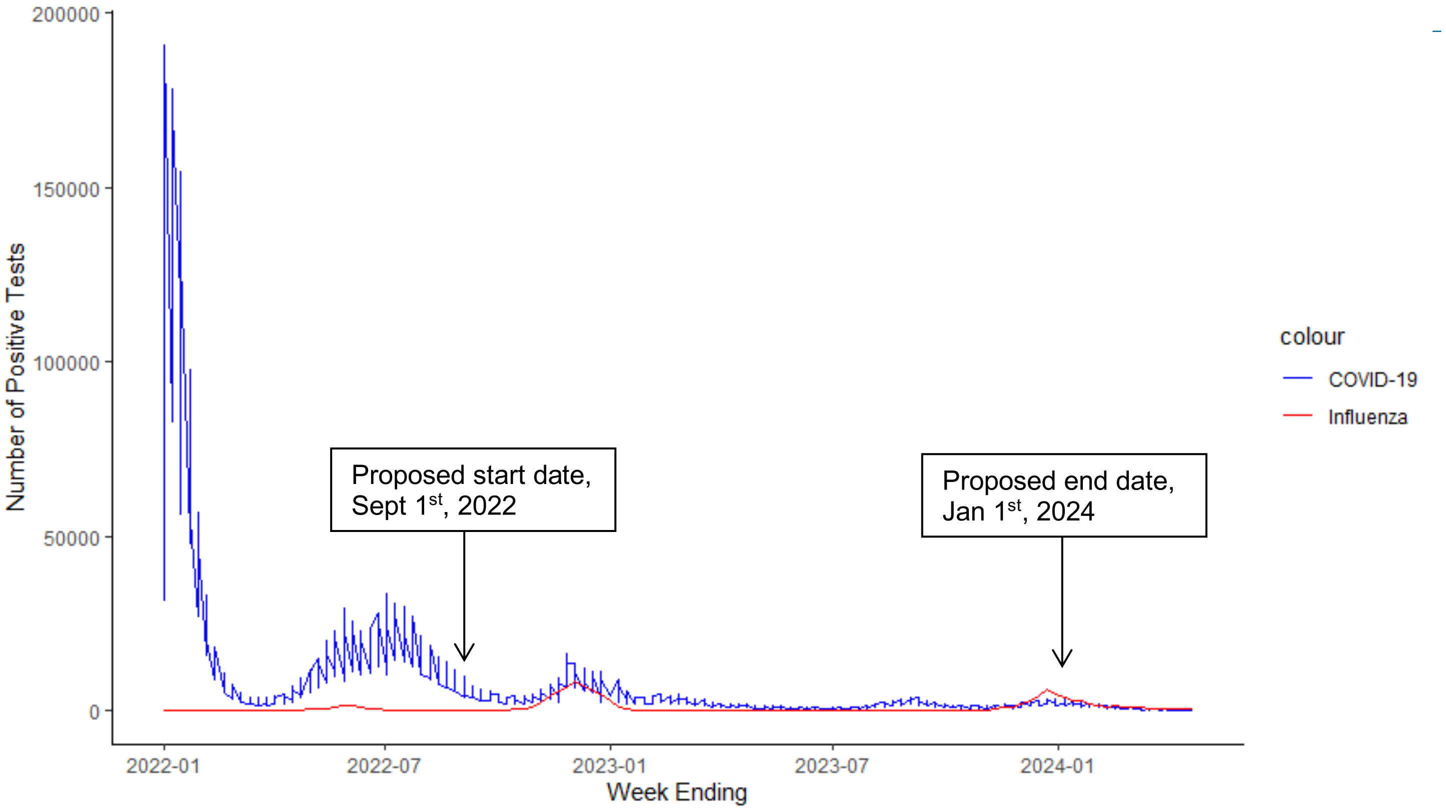

influenza tests over the study period, and different substantially by healthcare setting compared with COVID-19 antigen tests.

Units for observation within our analysis will be follow-up periods (31-180 days in length) after each eligible ARI episode associated with confirmed SARS-CoV-2 or influenza virus infection. We will require ≥1 year membership (allowing 45-day enrollment gap) prior to and including the positive test date.

Exclusion criteria

- Receipt of a positive SARS-CoV-2 or influenza virus test result within 180 days prior to index date: by this approach, the analysis will be limited to patients' first documented instance of ARI associated with SARS-CoV-2 or influenza over each 180 day period.
- SARS-CoV-2 positive tests among persons that received a COVID-19 vaccine within 14 days prior to or within 30 days after their SARS-CoV-2 positive test date. The rationale for this exclusion criteria is as follows: i) for patients that received vaccinations within 14 days prior to the date of infection, insufficient time may have elapsed to mount a full immune response and to consider that individual "vaccinated" prior to infection; ii) since follow-up for PAS outcomes begins 30 days after the SARS-CoV-2 positive test date and we aim to define exposure status prior to the SARS-CoV-2 positive test date, we will exclude individuals who received a COVID-19 vaccine within 30 days after their SARS-CoV-2 positive test date.
- Likewise, positive influenza test results among individuals who received an influenza vaccine within 14 days prior to 30 days after their positive influenza test date.
- Patients with missing information on age or sex, since these are important potential confounders required for stratification.

Analysis

Exposure groups. We will define infections as laboratory-confirmed SARS-CoV-2 diagnoses or laboratory-confirmed Influenza diagnoses via PCR tests with accompanying ARI symptoms and without any positive SARS-CoV-2 or Influenza test result within the preceding 180 days. The presence of ARI symptoms will be determined using an in-house Natural Language Processing (NLP) algorithm developed previously [9]. The index date will be defined as the date of eligible positive SARS-CoV-2 or influenza test results. For the main analyses, we plan to stratify SARS-CoV-2 and Influenza

positive patients into multiple exposure groups (Influenza A, Influenza B, and SARS-CoV-2 variant), according to date ranges during which differing variants were predominant in circulation in the study region as defined previously[10] .

Outcomes and follow-up. The primary analytical cohort will be followed for PAS endpoints in this study through 31–90 and 91–180 days after their index date, date of receipt of additional influenza or COVID-19 vaccine dose, date of re-infection (if re-infection occurs at least 90 days following index date) or secondary infection with SARS-CoV-2 or influenza, or date of disenrollment, whichever occurs earliest. Although there is no universal clinical case definition for PAS, we propose endpoints based on published CDC criteria for PCC [4] augmented with definitions from other published studies [11-14] to help ensure that the outcome is defined based on objective measures of healthcare resource utilization. PAS categories will be assessed separately using distinct and separate models, and overall risk of PAS will also be described. A full list of categories and sub-conditions is included in the appendix (**Appendix A**). Stratified analyses for PAS endpoints 31–90 days and 91-180 days after their index date will be conducted. PCC outcomes that occur during the first 30 days following the index date will still be considered as PAS if they re-occur during later time periods.

Covariates of interest. We will define prior COVID vaccine doses as the number of COVID doses received >14 days before individuals' index date. Influenza vaccination status will be defined as receipt of influenza vaccine >14 days in the year prior to positive Influenza test. Additional characteristics will be obtained from patient EHR and accompanying demographic metadata, including cases' age (categorized in 10-year increments for all analyses), sex, race/ethnicity (categorized as White non-Hispanic, Black non-Hispanic, Hispanic of any race, Asian, Pacific Islander, or other/mixed/unknown race/ethnicity), body mass index (categorized as underweight, normal weight, overweight, or obese if measured in the preceding year), history of cigarette smoking (current, former, or never smokers), prior-year healthcare utilization (categorized across outpatient, emergency department, and inpatient settings), Charlson comorbidity index (0, 1-2, 3-5, or ≥6), and neighborhood deprivation index (NDI). For participants with multiple doses documented within 7 days apart, the latter vaccination records will be treated as erroneous data entry (duplicates), and the earliest dose will be included in the analysis. Receipt of antivirals (e.g., Nirmatrelvir-Ritonavir for SARS-CoV-2 infections) will be categorized as dispenses initiated within 7 days after the index test date and dispenses initiated later than 7 days following the index date. Hospitalization will be defined as hospital admission ≤3 days prior to test or within 14 days after the index date.

Missing data. For variables with <5% missing data, an 'unknown' category will be used in the analyses. For variables with large amounts of missing data (>5%), we will populate 5 complete pseudo-datasets (including outcome variables) via multiple imputation using the Amelia package in R (24). For all analyses, we will pool results from replications across each pseudo-dataset.

Cross-sectional analysis. Characteristics of the study population will be described using mean or median and standard deviation for continuous variables and frequency and percentage for categorical variables.

Incident PCC. The overall and age-specific incidence of PCC per will be calculated separately for persons with each confirmed infection over the study period.

Risk Models. Follow-up time will start at the index date. Cox proportional hazards models will be used to assess the association of PAS diagnosis with SARS-CoV-2 or Influenza A/B infection. Time-varying hazard ratios (HRs) estimates and corresponding 95% confidence intervals will be adjusted for matching variables via regression strata and other confounders via covariate adjustment. We will define interaction terms for influenza virus infection and influenza vaccination, as well as SARS-CoV-2 infection and prior COVID-19 vaccination, time since most recent COVID-19 vaccine dose (<90, 90-180, >180 days), and receipt of antiviral treatment (e.g., molnupiravir or nirmatrelvir-ritonavir for SARS-CoV-2 treatment, and oseltamivir, zanamivir, peramivir, or baloxavir for influenza treatment, respectively). Risk models will be stratified by age (10-year age categories) and sex. Additional covariates for adjustment will include race/ethnicity (Hispanic, Black, Asian, White and Other/Unknown), Body-Mass Index (BMI), Smoking status (current, former, never), NDI, Charlson comorbidities in the year prior to the index date, healthcare utilization in the year prior (number of medical encounters across all settings), presence of any anxiety/depression diagnosis code, pre-existing presence of any PAS (so that exacerbations are compared to exacerbations and new onsets are compared to new onsets), and ARI diagnosis setting (outpatient, inpatient, emergency department or virtual care). The main analysis will assess the association between each viral infection and new-onset PAS or PAS exacerbation. Pre-existing conditions will be defined as documented diagnosis codes for any PAS category in EHR prior 1 calendar year to index date. We will censor observation periods at death, disenrollment, receipt of a positive test result (PCR or Antigen test administered in the healthcare setting) for the other virus (influenza virus infection for individuals in the SARS-CoV-2 exposure group, or

SARS-CoV-2 infection for those in the influenza exposure group), or end of study period (whichever occurs first). Statistical significance will be considered as two-sided p-values <0.05.

Subgroup analysis. We will perform pre-specified subgroup analysis on the primary PAS outcomes by type of infection (i.e., Influenza A or B, and SARS-CoV-2 variant), respiratory season (2022 and 2023). Additional pre-specified subgroup analyses will include the most recent COVID-19 vaccine dose received (1,2, 3+), receipt of antiviral treatment, and highest level of care received for the acute SARS-CoV-2 or influenza infection (ED, outpatient, virtual care, or Hospitalization 3 days prior to test or within 14 days after test). We will perform subgroup analyses by sex and age group, since prior evidence has suggested these factors modify associations with PAS.[15]

Optional aim: exacerbation of PAS following SARS-CoV-2 or Influenza. As an optional aim, we will perform additional analyses to assess the risk of PAS exacerbation, wherein analyses will be restricted to individuals with evidence of pre-existing syndrome-specific PAS within 1 year prior to the index date, and outcomes will be recorded as exacerbation of the same conditions. Syndrome-specific analyses will include only individuals with the specified PAS category of interest as a pre-existing condition.

References

1. *Centers for Disease Control and Prevention. Post-COVID Conditions: Information for Healthcare Providers.* March 1, 2023]; Available from: **https://www.cdc.gov/coronavirus/2019-ncov/hcp/clinical-care/post-covid-conditions.html**.
2. Gandhi, M., *Post-viral sequelae of COVID-19 and influenza.* 2023. **24**(3): p. 218-219.
3. Davis, H.E., L. McCorkell, J.M. Vogel, and E.J. Topol, *Long COVID: major findings, mechanisms and recommendations.* Nature Reviews Microbiology, 2023: p. 1-14.
4. Bull-Otterson, L., et al., *Post–COVID conditions among adult COVID-19 survivors aged 18–64 and≥ 65 years—United States, March 2020–November 2021.* Morbidity and Mortality Weekly Report, 2022. **71**(21): p. 713.
5. Ceban, F., et al., *Fatigue and cognitive impairment in Post-COVID-19 Syndrome: A systematic review and meta-analysis.* Brain Behav Immun, 2022. **101**: p. 93-135.
6. Parums, D.V., *Editorial: Long COVID, or Post-COVID Syndrome, and the Global Impact on Health Care.* Med Sci Monit, 2021. **27**: p. e933446.
7. Khullar, D., et al., *Racial/Ethnic Disparities in Post-acute Sequelae of SARS-CoV-2 Infection in New York: an EHR-Based Cohort Study from the RECOVER Program.* J Gen Intern Med, 2023: p. 1-10.
8. Al-Aly, Z., B. Bowe, and Y. Xie, *Long COVID after breakthrough SARS-CoV-2 infection.* Nature medicine, 2022. **28**(7): p. 1461-1467.
9. Malden, D., et al., *Natural Language Processing for Improved Characterization of COVID-19 Symptoms: Observational Study of 350,000 Patients in a Large Integrated Health Care System.* JMIR Public Health Surveill., 2022. **8**(12): p. e41529.
10. Lewnard, J.A., et al., *Immune escape and attenuated severity associated with the SARS-CoV-2 BA.2.86/JN.1 lineage.* medRxiv, 2024: p. 2024.04.17.24305964.
11. Daugherty, S.E., et al., *Risk of clinical sequelae after the acute phase of SARS-CoV-2 infection: retrospective cohort study.* BMJ, 2021. **373**: p. n1098.
12. Cohen, K., et al., *Risk of persistent and new clinical sequelae among adults aged 65 years and older during the post-acute phase of SARS-CoV-2 infection: retrospective cohort study.* BMJ, 2022. **376**: p. e068414.
13. Taquet, M., Q. Dercon, and P.J. Harrison, *Six-month sequelae of post-vaccination SARS-CoV-2 infection: A retrospective cohort study of 10,024 breakthrough infections.* Brain Behav Immun, 2022. **103**: p. 154-162.
14. Tartof, S.Y., et al., *Health Care Utilization in the 6 Months Following SARS-CoV-2 Infection.* JAMA Netw Open, 2022. **5**(8): p. e2225657.
15. Fischer, H.A.-O., et al., *Development and validation of a prediction algorithm to identify birth in countries with high tuberculosis incidence in two large California health systems.* PLoS One, 2022. **17(8)**(1932-6203).

Appendix A. ICD-10 codes used to define PAS

ICD10 code	Description	Sub-condition
**Cardiovascular system disorders**		
**I200**	Unstable angina	Cardiovascular disease
**I201**	Angina pectoris with documented spasm	Cardiovascular disease
**I208**	Other forms of angina pectoris	Cardiovascular disease
**I209**	Angina pectoris, unspecified	Cardiovascular disease
**I240**	Acute coronary thrombosis not resulting in myocardial infarction	Cardiovascular disease
**I248**	Other forms of acute ischemic heart disease	Cardiovascular disease
**I249**	Acute ischemic heart disease, unspecified	Cardiovascular disease
**I2510**	Atherosclerotic heart disease of native coronary artery without angina pectoris	Cardiovascular disease
**I25110**	Atherosclerotic heart disease of native coronary artery with unstable angina pectoris	Cardiovascular disease
**I25111**	Atherosclerotic heart disease of native coronary artery with angina pectoris with	Cardiovascular disease
**I25118**	Atherosclerotic heart disease of native coronary artery with other forms of	Cardiovascular disease
**I25119**	Atherosclerotic heart disease of native coronary artery with unspecified angina pectoris	Cardiovascular disease
**I256**	Silent myocardial ischemia	Cardiovascular disease
**I25700**	Atherosclerosis of coronary artery bypass graft(s), unspecified, with unstable angina pectoris	Cardiovascular disease
**I25701**	Atherosclerosis of coronary artery bypass graft(s), unspecified, with angina pectoris with	Cardiovascular disease
**I25708**	Atherosclerosis of coronary artery bypass graft(s), unspecified, with other forms of	Cardiovascular disease
**I25709**	Atherosclerosis of coronary artery bypass graft(s), unspecified, with unspecified angina pectoris	Cardiovascular disease
**I25710**	Atherosclerosis of autologous vein coronary artery bypass graft(s) with unstable angina	Cardiovascular disease
**I25711**	Atherosclerosis of autologous vein coronary artery bypass graft(s) with angina pectoris	Cardiovascular disease
**I25718**	Atherosclerosis of autologous vein coronary artery bypass graft(s) with other forms	Cardiovascular disease
**I25719**	Atherosclerosis of autologous vein coronary artery bypass graft(s) with unspecified angina	Cardiovascular disease
**I25720**	Atherosclerosis of autologous artery coronary artery bypass graft(s) with unstable angina	Cardiovascular disease
**I25721**	Atherosclerosis of autologous artery coronary artery bypass graft(s) with angina pectoris	Cardiovascular disease
**I25728**	Atherosclerosis of autologous artery coronary artery bypass graft(s) with other forms	Cardiovascular disease
**I25729**	Atherosclerosis of autologous artery coronary artery bypass graft(s) with unspecified angina	Cardiovascular disease
**I25730**	Atherosclerosis of nonautologous biological coronary artery bypass graft(s) with unstable angina	Cardiovascular disease
**I25731**	Atherosclerosis of nonautologous biological coronary artery bypass graft(s) with angina pectoris	Cardiovascular disease
**I25738**	Atherosclerosis of nonautologous biological coronary artery bypass graft(s) with other forms	Cardiovascular disease
**I25739**	Atherosclerosis of nonautologous biological coronary artery bypass graft(s) with unspecified angina	Cardiovascular disease
**I25750**	Atherosclerosis of native coronary artery of transplanted heart with unstable angina	Cardiovascular disease
**I25751**	Atherosclerosis of native coronary artery of transplanted heart with angina pectoris	Cardiovascular disease
**I25758**	Atherosclerosis of native coronary artery of transplanted heart with other forms	Cardiovascular disease
**I25759**	Atherosclerosis of native coronary artery of transplanted heart with unspecified angina	Cardiovascular disease
**I25760**	Atherosclerosis of bypass graft of coronary artery of transplanted heart with	Cardiovascular disease
**I25761**	Atherosclerosis of bypass graft of coronary artery of transplanted heart with	Cardiovascular disease
**I25768**	Atherosclerosis of bypass graft of coronary artery of transplanted heart with	Cardiovascular disease
**I25769**	Atherosclerosis of bypass graft of coronary artery of transplanted heart with	Cardiovascular disease
**I25790**	Atherosclerosis of other coronary artery bypass graft(s) with unstable angina pectoris	Cardiovascular disease
**I25791**	Atherosclerosis of other coronary artery bypass graft(s) with angina pectoris with	Cardiovascular disease
**I25798**	Atherosclerosis of other coronary artery bypass graft(s) with other forms of	Cardiovascular disease
**I25799**	Atherosclerosis of other coronary artery bypass graft(s) with unspecified angina pectoris	Cardiovascular disease
**I25810**	Atherosclerosis of coronary artery bypass graft(s) without angina pectoris	Cardiovascular disease
**I25811**	Atherosclerosis of native coronary artery of transplanted heart without angina pectoris	Cardiovascular disease
**I25812**	Atherosclerosis of bypass graft of coronary artery of transplanted heart without	Cardiovascular disease
**I2582**	Chronic total occlusion of coronary artery	Cardiovascular disease
**I2583**	Coronary atherosclerosis due to lipid rich plaque	Cardiovascular disease
**I2584**	Coronary atherosclerosis due to calcified coronary lesion	Cardiovascular disease
**I2589**	Other forms of chronic ischemic heart disease	Cardiovascular disease
**I259**	Chronic ischemic heart disease, unspecified	Cardiovascular disease
**Z9861**	Coronary angioplasty status	Cardiovascular disease
**I470**	Re-entry ventricular arrhythmia	Cardiac dysrhythmia
**I471**	Supraventricular tachycardia	Cardiac dysrhythmia
**I472**	Ventricular tachycardia	Cardiac dysrhythmia
**I4720**	Ventricular tachycardia unspecified	Cardiac dysrhythmia
**I4721**	torsades de pointed	Cardiac dysrhythmia
**I4729**	Other ventricular tachycardia	Cardiac dysrhythmia
**I479**	Paroxysmal tachycardia, unspecified	Cardiac dysrhythmia
**I480**	Paroxysmal atrial fibrillation	Cardiac dysrhythmia
**I481**	Persistent atrial fibrillation	Cardiac dysrhythmia
**I4811**	Longstanding persistent atrial fibrillation	Cardiac dysrhythmia
**I4819**	Other persistent atrial fibrillation	Cardiac dysrhythmia
**I4820**	Unspecified chronic atrial fibrillation	Cardiac dysrhythmia

**I4821**	Permanent atrial fibrillation	Cardiac dysrhythmia
**I483**	Typical atrial flutter	Cardiac dysrhythmia
**I484**	Atypical atrial flutter	Cardiac dysrhythmia
**I4891**	Unspecified atrial fibrillation	Cardiac dysrhythmia
**I4892**	Unspecified atrial flutter	Cardiac dysrhythmia
**I4901**	Ventricular fibrillation	Cardiac dysrhythmia
**I4902**	Ventricular flutter	Cardiac dysrhythmia
**I491**	Atrial premature depolarization	Cardiac dysrhythmia
**I492**	Junctional premature depolarization	Cardiac dysrhythmia
**I493**	Ventricular premature depolarization	Cardiac dysrhythmia
**I4940**	Unspecified premature depolarization	Cardiac dysrhythmia
**I4949**	Other premature depolarization	Cardiac dysrhythmia
**I495**	Sick sinus syndrome	Cardiac dysrhythmia
**I498**	Other specified cardiac arrhythmias	Cardiac dysrhythmia
**I499**	Cardiac arrhythmia, unspecified	Cardiac dysrhythmia
**R000**	Tachycardia, unspecified	Cardiac dysrhythmia
**R001**	Bradycardia, unspecified	Cardiac dysrhythmia
**R002**	Palpitations	Cardiac dysrhythmia
**R008**	Other abnormalities of heart beat	Cardiac dysrhythmia
**R009**	Unspecified abnormalities of heart beat	Cardiac dysrhythmia
**R010**	Benign and innocent cardiac murmurs	Cardiac dysrhythmia
**R011**	Cardiac murmur, unspecified	Cardiac dysrhythmia
**R012**	Other cardiac sounds	Cardiac dysrhythmia
**R030**	Elevated blood-pressure reading, without diagnosis of hypertension	Cardiac dysrhythmia
**R0989**	Other specified symptoms and signs involving the circulatory and respiratory systems	CHF
**I050**	Rheumatic mitral stenosis	CHF
**I051**	Rheumatic mitral insufficiency	CHF
**I052**	Rheumatic mitral stenosis with insufficiency	CHF
**I058**	Other rheumatic mitral valve diseases	CHF
**I059**	Rheumatic mitral valve disease, unspecified	CHF
**I060**	Rheumatic aortic stenosis	CHF
**I061**	Rheumatic aortic insufficiency	CHF
**I062**	Rheumatic aortic stenosis with insufficiency	CHF
**I068**	Other rheumatic aortic valve diseases	CHF
**I069**	Rheumatic aortic valve disease, unspecified	CHF
**I070**	Rheumatic tricuspid stenosis	CHF
**I071**	Rheumatic tricuspid insufficiency	CHF
**I072**	Rheumatic tricuspid stenosis and insufficiency	CHF
**I078**	Other rheumatic tricuspid valve diseases	CHF
**I079**	Rheumatic tricuspid valve disease, unspecified	CHF
**I080**	Rheumatic disorders of both mitral and aortic valves	CHF
**I081**	Rheumatic disorders of both mitral and tricuspid valves	CHF
**I082**	Rheumatic disorders of both aortic and tricuspid valves	CHF
**I083**	Combined rheumatic disorders of mitral, aortic and tricuspid valves	CHF
**I088**	Other rheumatic multiple valve diseases	CHF
**I089**	Rheumatic multiple valve disease, unspecified	CHF
**I090**	Rheumatic myocarditis	CHF
**I091**	Rheumatic diseases of endocardium, valve unspecified	CHF
**I092**	Chronic rheumatic pericarditis	CHF
**I0981**	Rheumatic heart failure	CHF
**I0989**	Other specified rheumatic heart diseases	CHF
**I502**	Other CHF	CHF
**I503**	Other CHF	CHF
**I099**	Rheumatic heart disease, unspecified	CHF
**I2101**	ST elevation (STEMI) myocardial infarction involving left main coronary artery	Acute MI
**I2102**	ST elevation (STEMI) myocardial infarction involving left anterior descending coronary artery	Acute MI
**I2109**	ST elevation (STEMI) myocardial infarction involving other coronary artery of anterior	Acute MI
**I2111**	ST elevation (STEMI) myocardial infarction involving right coronary artery	Acute MI
**I2119**	ST elevation (STEMI) myocardial infarction involving other coronary artery of inferior	Acute MI
**I2121**	ST elevation (STEMI) myocardial infarction involving left circumflex coronary artery	Acute MI
**I2129**	ST elevation (STEMI) myocardial infarction involving other sites	Acute MI
**I213**	ST elevation (STEMI) myocardial infarction of unspecified site	Acute MI
**I214**	Non-ST elevation (NSTEMI) myocardial infarction	Acute MI
**I219**	Acute myocardial infarction, unspecified	Acute MI
**I21A1**	Myocardial infarction type 2	Acute MI
**I21A9**	Other myocardial infarction type	Acute MI
**I220**	Subsequent ST elevation (STEMI) myocardial infarction of anterior wall	Acute MI
**I221**	Subsequent ST elevation (STEMI) myocardial infarction of inferior wall	Acute MI

**I222**	Subsequent non-ST elevation (NSTEMI) myocardial infarction	Acute MI
**I228**	Subsequent ST elevation (STEMI) myocardial infarction of other sites	Acute MI
**I229**	Subsequent ST elevation (STEMI) myocardial infarction of unspecified site	Acute MI
**A3681**	Diphtheritic cardiomyopathy	Myocarditis and cardiomyopathy
**B3320**	Viral carditis, unspecified	Myocarditis and cardiomyopathy
**B3322**	Viral myocarditis	Myocarditis and cardiomyopathy
**B3324**	Viral cardiomyopathy	Myocarditis and cardiomyopathy
**B5881**	Toxoplasma myocarditis	Myocarditis and cardiomyopathy
**I255**	Ischemic cardiomyopathy	Myocarditis and cardiomyopathy
**I400**	Infective myocarditis	Myocarditis and cardiomyopathy
**I401**	Isolated myocarditis	Myocarditis and cardiomyopathy
**I408**	Other acute myocarditis	Myocarditis and cardiomyopathy
**I409**	Acute myocarditis, unspecified	Myocarditis and cardiomyopathy
**I41**	Myocarditis in diseases classified elsewhere	Myocarditis and cardiomyopathy
**I420**	Dilated cardiomyopathy	Myocarditis and cardiomyopathy
**I421**	Obstructive hypertrophic cardiomyopathy	Myocarditis and cardiomyopathy
**I422**	Other hypertrophic cardiomyopathy	Myocarditis and cardiomyopathy
**I423**	Endomyocardial (eosinophilic) disease	Myocarditis and cardiomyopathy
**I424**	Endocardial fibroelastosis	Myocarditis and cardiomyopathy
**I425**	Other restrictive cardiomyopathy	Myocarditis and cardiomyopathy
**I428**	Other cardiomyopathies	Myocarditis and cardiomyopathy
**I429**	Cardiomyopathy, unspecified	Myocarditis and cardiomyopathy
**I43**	Cardiomyopathy in diseases classified elsewhere	Myocarditis and cardiomyopathy
**I514**	Myocarditis, unspecified	Myocarditis and cardiomyopathy
**J1082**	Influenza due to other identified influenza virus with myocarditis	Myocarditis and cardiomyopathy
**J1182**	Influenza due to unidentified influenza virus with myocarditis	Myocarditis and cardiomyopathy
**O903**	Peripartum cardiomyopathy	Myocarditis and cardiomyopathy
**B3321**	Viral endocarditis	Myocarditis and cardiomyopathy
**I27**	Pulmonary hypertension (all subcodes)	Pulmonary hypertension
**I10**	Essential (primary) hypertension	Hypertension
**I11**	Hypertensive heart disease	Hypertension
**I12**	Hypertensive chronic kidney disease	Hypertension
**I13**	Hypertensive heart and chronic kidney disease	Hypertension
**I15**	Secondary hypertension	Hypertension
**I16**	Hypertensive crisis	Hypertension
**R570**	Cardiogenic shock	Cardiogenic shock
**Hemolytic and vascular system disorders**		
**D473**	Essential (hemorrhagic) thrombocythemia	Coagulation and hemorrhagic
**D65**	Disseminated intravascular coagulation [defibrination syndrome]	Coagulation and hemorrhagic
**D68311**	Acquired hemophilia	Coagulation and hemorrhagic
**D68312**	Antiphospholipid antibody with hemorrhagic disorder	Coagulation and hemorrhagic
**D68318**	Other hemorrhagic disorder due to intrinsic circulating anticoagulants, antibodies, or inhibitors	Coagulation and hemorrhagic
**D6832**	Hemorrhagic disorder due to extrinsic circulating anticoagulants	Coagulation and hemorrhagic
**D684**	Acquired coagulation factor deficiency	Coagulation and hemorrhagic
**D6851**	Activated protein C resistance	Coagulation and hemorrhagic
**D6852**	Prothrombin gene mutation	Coagulation and hemorrhagic
**D6859**	Other primary thrombophilia	Coagulation and hemorrhagic
**D6861**	Antiphospholipid syndrome	Coagulation and hemorrhagic
**D6862**	Lupus anticoagulant syndrome	Coagulation and hemorrhagic
**D6869**	Other thrombophilia	Coagulation and hemorrhagic
**D688**	Other specified coagulation defects	Coagulation and hemorrhagic
**D689**	Coagulation defect, unspecified	Coagulation and hemorrhagic
**D690**	Allergic purpura	Coagulation and hemorrhagic
**D691**	Qualitative platelet defects	Coagulation and hemorrhagic
**D692**	Other nonthrombocytopenic purpura	Coagulation and hemorrhagic
**D693**	Immune thrombocytopenic purpura	Coagulation and hemorrhagic
**D6941**	Evans syndrome	Coagulation and hemorrhagic
**D6942**	Congenital and hereditary thrombocytopenia purpura	Coagulation and hemorrhagic
**D6949**	Other primary thrombocytopenia	Coagulation and hemorrhagic
**D6951**	Posttransfusion purpura	Coagulation and hemorrhagic
**D6959**	Other secondary thrombocytopenia	Coagulation and hemorrhagic
**D696**	Thrombocytopenia, unspecified	Coagulation and hemorrhagic
**D698**	Other specified hemorrhagic conditions	Coagulation and hemorrhagic
**D699**	Hemorrhagic condition, unspecified	Coagulation and hemorrhagic
**D7582**	Heparin induced thrombocytopenia (HIT)	Coagulation and hemorrhagic
**D75838**	Other thrombocytosis	Coagulation and hemorrhagic
**D75839**	Thrombocytosis, unspecified	Coagulation and hemorrhagic
**M362**	Hemophilic arthropathy	Coagulation and hemorrhagic

**D473**	Essential (hemorrhagic) thrombocythemia	Coagulation and hemorrhagic
**D65**	Disseminated intravascular coagulation [defibrination syndrome]	Coagulation and hemorrhagic
**D68311**	Acquired hemophilia	Coagulation and hemorrhagic
**D68312**	Antiphospholipid antibody with hemorrhagic disorder	Coagulation and hemorrhagic
**D68318**	Other hemorrhagic disorder due to intrinsic circulating anticoagulants, antibodies, or inhibitors	Coagulation and hemorrhagic
**D6832**	Hemorrhagic disorder due to extrinsic circulating anticoagulants	Coagulation and hemorrhagic
**D684**	Acquired coagulation factor deficiency	Coagulation and hemorrhagic
**D6851**	Activated protein C resistance	Coagulation and hemorrhagic
**D6852**	Prothrombin gene mutation	Coagulation and hemorrhagic
**D6859**	Other primary thrombophilia	Coagulation and hemorrhagic
**D6861**	Antiphospholipid syndrome	Coagulation and hemorrhagic
**D6862**	Lupus anticoagulant syndrome	Coagulation and hemorrhagic
**D6869**	Other thrombophilia	Coagulation and hemorrhagic
**D688**	Other specified coagulation defects	Coagulation and hemorrhagic
**D689**	Coagulation defect, unspecified	Coagulation and hemorrhagic
**D690**	Allergic purpura	Coagulation and hemorrhagic
**D691**	Qualitative platelet defects	Coagulation and hemorrhagic
**D692**	Other nonthrombocytopenic purpura	Coagulation and hemorrhagic
**D693**	Immune thrombocytopenic purpura	Coagulation and hemorrhagic
**D6941**	Evans syndrome	Coagulation and hemorrhagic
**D6942**	Congenital and hereditary thrombocytopenia purpura	Coagulation and hemorrhagic
**D6949**	Other primary thrombocytopenia	Coagulation and hemorrhagic
**D6951**	Posttransfusion purpura	Coagulation and hemorrhagic
**D6959**	Other secondary thrombocytopenia	Coagulation and hemorrhagic
**D696**	Thrombocytopenia, unspecified	Coagulation and hemorrhagic
**D698**	Other specified hemorrhagic conditions	Coagulation and hemorrhagic
**D699**	Hemorrhagic condition, unspecified	Coagulation and hemorrhagic
**D7582**	Heparin induced thrombocytopenia (HIT)	Coagulation and hemorrhagic
**D75838**	Other thrombocytosis	Coagulation and hemorrhagic
**D75839**	Thrombocytosis, unspecified	Coagulation and hemorrhagic
**M362**	Hemophilic arthropathy	Coagulation and hemorrhagic
**D686**	Other thrombophilia	Coagulation and hemorrhagic
**D72.819**	Decreased white blood cell count	Coagulation and hemorrhagic
**I82401**	Acute embolism and thrombosis of unspecified deep veins of right lower	Thromboembolic event
**I82402**	Acute embolism and thrombosis of unspecified deep veins of left lower	Thromboembolic event
**I82403**	Acute embolism and thrombosis of unspecified deep veins of lower extremity,	Thromboembolic event
**I82409**	Acute embolism and thrombosis of unspecified deep veins of unspecified lower	Thromboembolic event
**I82491**	Acute embolism and thrombosis of other specified deep vein of right	Thromboembolic event
**I82492**	Acute embolism and thrombosis of other specified deep vein of left	Thromboembolic event
**I82493**	Acute embolism and thrombosis of other specified deep vein of lower	Thromboembolic event
**I82499**	Acute embolism and thrombosis of other specified deep vein of unspecified	Thromboembolic event
**I824Y1**	Acute embolism and thrombosis of unspecified deep veins of right proximal	Thromboembolic event
**I824Y2**	Acute embolism and thrombosis of unspecified deep veins of left proximal	Thromboembolic event
**I824Y3**	Acute embolism and thrombosis of unspecified deep veins of proximal lower	Thromboembolic event
**I824Y9**	Acute embolism and thrombosis of unspecified deep veins of unspecified proximal	Thromboembolic event
**I824Z1**	Acute embolism and thrombosis of unspecified deep veins of right distal	Thromboembolic event
**I824Z2**	Acute embolism and thrombosis of unspecified deep veins of left distal	Thromboembolic event
**I824Z3**	Acute embolism and thrombosis of unspecified deep veins of distal lower	Thromboembolic event
**I824Z9**	Acute embolism and thrombosis of unspecified deep veins of unspecified distal	Thromboembolic event
**I82621**	Acute embolism and thrombosis of deep veins of right upper extremity	Thromboembolic event
**I82622**	Acute embolism and thrombosis of deep veins of left upper extremity	Thromboembolic event
**I82623**	Acute embolism and thrombosis of deep veins of upper extremity, bilateral	Thromboembolic event
**I82629**	Acute embolism and thrombosis of deep veins of unspecified upper extremity	Thromboembolic event
**I82501**	Chronic embolism and thrombosis of unspecified deep veins of right lower	Thromboembolic event
**I82502**	Chronic embolism and thrombosis of unspecified deep veins of left lower	Thromboembolic event
**I82503**	Chronic embolism and thrombosis of unspecified deep veins of lower extremity,	Thromboembolic event
**I82509**	Chronic embolism and thrombosis of unspecified deep veins of unspecified lower	Thromboembolic event
**I82591**	Chronic embolism and thrombosis of other specified deep vein of right	Thromboembolic event
**I82592**	Chronic embolism and thrombosis of other specified deep vein of left	Thromboembolic event
**I82593**	Chronic embolism and thrombosis of other specified deep vein of lower	Thromboembolic event
**I82599**	Chronic embolism and thrombosis of other specified deep vein of unspecified	Thromboembolic event
**I825Y1**	Chronic embolism and thrombosis of unspecified deep veins of right proximal	Thromboembolic event
**I825Y2**	Chronic embolism and thrombosis of unspecified deep veins of left proximal	Thromboembolic event
**I825Y3**	Chronic embolism and thrombosis of unspecified deep veins of proximal lower	Thromboembolic event
**I825Y9**	Chronic embolism and thrombosis of unspecified deep veins of unspecified proximal	Thromboembolic event
**I825Z1**	Chronic embolism and thrombosis of unspecified deep veins of right distal	Thromboembolic event
**I825Z2**	Chronic embolism and thrombosis of unspecified deep veins of left distal	Thromboembolic event
**I825Z3**	Chronic embolism and thrombosis of unspecified deep veins of distal lower	Thromboembolic event

**I825Z9**	Chronic embolism and thrombosis of unspecified deep veins of unspecified distal	Thromboembolic event
**I82721**	Chronic embolism and thrombosis of deep veins of right upper extremity	Thromboembolic event
**I82722**	Chronic embolism and thrombosis of deep veins of left upper extremity	Thromboembolic event
**I82723**	Chronic embolism and thrombosis of deep veins of upper extremity, bilateral	Thromboembolic event
**I82729**	Chronic embolism and thrombosis of deep veins of unspecified upper extremity	Thromboembolic event
**I82**	Other venous embolism and thrombosis (all subcodes)	Thromboembolic event
**G460**	Middle cerebral artery syndrome	Cerebrovascular disease
**G461**	Anterior cerebral artery syndrome	Cerebrovascular disease
**G462**	Posterior cerebral artery syndrome	Cerebrovascular disease
**G463**	Brain stem stroke syndrome	Cerebrovascular disease
**G464**	Cerebellar stroke syndrome	Cerebrovascular disease
**G465**	Pure motor lacunar syndrome	Cerebrovascular disease
**G466**	Pure sensory lacunar syndrome	Cerebrovascular disease
**G467**	Other lacunar syndromes	Cerebrovascular disease
**G468**	Other vascular syndromes of brain in cerebrovascular diseases	Cerebrovascular disease
**I671**	Cerebral aneurysm, nonruptured	Cerebrovascular disease
**I672**	Cerebral atherosclerosis	Cerebrovascular disease
**I673**	Progressive vascular leukoencephalopathy	Cerebrovascular disease
**I675**	Moyamoya disease	Cerebrovascular disease
**I676**	Nonpyogenic thrombosis of intracranial venous system	Cerebrovascular disease
**I677**	Cerebral arteritis, not elsewhere classified	Cerebrovascular disease
**I6781**	Acute cerebrovascular insufficiency	Cerebrovascular disease
**I6782**	Cerebral ischemia	Cerebrovascular disease
**I6783**	Posterior reversible encephalopathy syndrome	Cerebrovascular disease
**I67841**	Reversible cerebrovascular vasoconstriction syndrome	Cerebrovascular disease
**I67848**	Other cerebrovascular vasospasm and vasoconstriction	Cerebrovascular disease
**I67850**	Cerebral autosomal dominant arteriopathy with subcortical infarcts and leukoencephalopathy	Cerebrovascular disease
**I67858**	Other hereditary cerebrovascular disease	Cerebrovascular disease
**I6789**	Other cerebrovascular disease	Cerebrovascular disease
**I679**	Cerebrovascular disease, unspecified	Cerebrovascular disease
**I680**	Cerebral amyloid angiopathy	Cerebrovascular disease
**I682**	Cerebral arteritis in other diseases classified elsewhere	Cerebrovascular disease
**I688**	Other cerebrovascular disorders in diseases classified elsewhere	Cerebrovascular disease
**I60**	Nontraumatic subarachnoid hemorrhage	Cerebrovascular disease
**I61**	Nontraumatic intracerebral hemorrhage	Cerebrovascular disease
**I62**	Other and unspecified nontraumatic intracranial hemorrhage	Cerebrovascular disease
**I63**	Cerebral infarction	Cerebrovascular disease
**I69**	Sequelae of cerebrovascular disease	Cerebrovascular disease
**I679**	Cerebrovascular disease, unspecified	Cerebrovascular disease
**I680**	Cerebral amyloid angiopathy	Cerebrovascular disease
**I682**	Cerebral arteritis in other diseases classified elsewhere	Cerebrovascular disease
**I688**	Other cerebrovascular disorders in diseases classified elsewhere	Cerebrovascular disease
**I60**	Nontraumatic subarachnoid hemorrhage	Cerebrovascular disease
**I61**	Nontraumatic intracerebral hemorrhage	Cerebrovascular disease
**I62**	Other and unspecified nontraumatic intracranial hemorrhage	Cerebrovascular disease
**I63**	Cerebral infarction	Cerebrovascular disease
**I69**	Sequelae of cerebrovascular disease	Cerebrovascular disease
**Respiratory System disorders**		
**J4520**	Mild intermittent asthma, uncomplicated	Asthma
**J4521**	Mild intermittent asthma with (acute) exacerbation	Asthma
**J4522**	Mild intermittent asthma with status asthmaticus	Asthma
**J4530**	Mild persistent asthma, uncomplicated	Asthma
**J4531**	Mild persistent asthma with (acute) exacerbation	Asthma
**J4532**	Mild persistent asthma with status asthmaticus	Asthma
**J4540**	Moderate persistent asthma, uncomplicated	Asthma
**J4541**	Moderate persistent asthma with (acute) exacerbation	Asthma
**J4542**	Moderate persistent asthma with status asthmaticus	Asthma
**J4550**	Severe persistent asthma, uncomplicated	Asthma
**J4551**	Severe persistent asthma with (acute) exacerbation	Asthma
**J4552**	Severe persistent asthma with status asthmaticus	Asthma
**J45901**	Unspecified asthma with (acute) exacerbation	Asthma
**J45902**	Unspecified asthma with status asthmaticus	Asthma
**J45909**	Unspecified asthma, uncomplicated	Asthma
**J45990**	Exercise induced bronchospasm	Asthma
**J45991**	Cough variant asthma	Asthma
**J45998**	Other asthma	Asthma
**R040**	Epistaxis	Respiratory symptoms
**R041**	Hemorrhage from throat	Respiratory symptoms

**R042**	Hemoptysis	Respiratory symptoms
**R0481**	Acute idiopathic pulmonary hemorrhage in infants	Respiratory symptoms
**R0489**	Hemorrhage from other sites in respiratory passages	Respiratory symptoms
**R049**	Hemorrhage from respiratory passages, unspecified	Respiratory symptoms
**R05**	Cough	Respiratory symptoms
**R051**	Acute cough	Respiratory symptoms
**R052**	Subacute cough	Respiratory symptoms
**R053**	Chronic cough	Respiratory symptoms
**R054**	Cough syncope	Respiratory symptoms
**R058**	Other specified cough	Respiratory symptoms
**R059**	Cough, unspecified	Respiratory symptoms
**R0600**	Dyspnea, unspecified	Respiratory symptoms
**R0601**	Orthopnea	Respiratory symptoms
**R0602**	Shortness of breath	Respiratory symptoms
**R0603**	Acute respiratory distress	Respiratory symptoms
**R0609**	Other forms of dyspnea	Respiratory symptoms
**R061**	Stridor	Respiratory symptoms
**R062**	Wheezing	Respiratory symptoms
**R064**	Hyperventilation	Respiratory symptoms
**R066**	Hiccough	Respiratory symptoms
**R067**	Sneezing	Respiratory symptoms
**R0681**	Apnea, not elsewhere classified	Respiratory symptoms
**R0682**	Tachypnea, not elsewhere classified	Respiratory symptoms
**R0689**	Other abnormalities of breathing	Respiratory symptoms
**R069**	Unspecified abnormalities of breathing	Respiratory symptoms
**R070**	Pain in throat	Respiratory symptoms
**R071**	Chest pain on breathing	Respiratory symptoms
**R0781**	Pleurodynia	Respiratory symptoms
**R0782**	Intercostal pain	Respiratory symptoms
**R0901**	Asphyxia	Respiratory symptoms
**R0902**	Hypoxemia	Respiratory symptoms
**R093**	Abnormal sputum	Respiratory symptoms
**R0981**	Nasal congestion	Respiratory symptoms
**R0982**	Postnasal drip	Respiratory symptoms
**R072**	pleurodynia	Respiratory symptoms
**R078**	intercostal	Respiratory symptoms
**R079**	other chest pain	Respiratory symptoms
**I2601**	Septic pulmonary embolism with acute cor pulmonale	Acute pulmonary embolism
**I2602**	Saddle embolus of pulmonary artery with acute cor pulmonale	Acute pulmonary embolism
**I2609**	Other pulmonary embolism with acute cor pulmonale	Acute pulmonary embolism
**I2690**	Septic pulmonary embolism without acute cor pulmonale	Acute pulmonary embolism
**I2692**	Saddle embolus of pulmonary artery without acute cor pulmonale	Acute pulmonary embolism
**I2693**	Single subsegmental pulmonary embolism without acute cor pulmonale	Acute pulmonary embolism
**I2694**	Multiple subsegmental pulmonary emboli without acute cor pulmonale	Acute pulmonary embolism
**I2699**	Other pulmonary embolism without acute cor pulmonale	Acute pulmonary embolism
**J96.10**	Chronic respiratory failure unspecified whether with hypoxia or hypercapnia	COPD and Bronchitis
**J96.11**	Chronic respiratory failure WITH HYPOXIA	COPD and Bronchitis
**J96.12**	Chronic respiratory failure WITH HYPERCAPNIA	COPD and Bronchitis
**J20**	Acute bronchitis	COPD and Bronchitis
**J40**	Bronchitis, not specified as acute or chronic	COPD and Bronchitis
**J41**	Simple and mucopurulent chronic bronchitis	COPD and Bronchitis
**J42**	Unspecified chronic bronchitis	COPD and Bronchitis
**J84**	Interstitial lung disease	Interstitial lung disease
**J960**	Acute respiratory failure	Acute respiratory failure
**J81**	Pulmonary edema	Pulmonary edema
**J4520**	Mild intermittent asthma, uncomplicated	Asthma
**J4521**	Mild intermittent asthma with (acute) exacerbation	Asthma
**J4522**	Mild intermittent asthma with status asthmaticus	Asthma
**J4530**	Mild persistent asthma, uncomplicated	Asthma
**J4531**	Mild persistent asthma with (acute) exacerbation	Asthma
**J4532**	Mild persistent asthma with status asthmaticus	Asthma
**J4540**	Moderate persistent asthma, uncomplicated	Asthma
**J4541**	Moderate persistent asthma with (acute) exacerbation	Asthma
**J4542**	Moderate persistent asthma with status asthmaticus	Asthma
**J4550**	Severe persistent asthma, uncomplicated	Asthma
**J4551**	Severe persistent asthma with (acute) exacerbation	Asthma
**J4552**	Severe persistent asthma with status asthmaticus	Asthma
**J45901**	Unspecified asthma with (acute) exacerbation	Asthma

**J45902**	Unspecified asthma with status asthmaticus	Asthma
**J45909**	Unspecified asthma, uncomplicated	Asthma
**J45990**	Exercise induced bronchospasm	Asthma
**J45991**	Cough variant asthma	Asthma
**J45998**	Other asthma	Asthma
**R040**	Epistaxis	Respiratory symptoms
**R041**	Hemorrhage from throat	Respiratory symptoms
**R042**	Hemoptysis	Respiratory symptoms
**R0481**	Acute idiopathic pulmonary hemorrhage in infants	Respiratory symptoms
**R0489**	Hemorrhage from other sites in respiratory passages	Respiratory symptoms
**R049**	Hemorrhage from respiratory passages, unspecified	Respiratory symptoms
**R05**	Cough	Respiratory symptoms
**R051**	Acute cough	Respiratory symptoms
**R052**	Subacute cough	Respiratory symptoms
**R053**	Chronic cough	Respiratory symptoms
**R054**	Cough syncope	Respiratory symptoms
**R058**	Other specified cough	Respiratory symptoms
**R059**	Cough, unspecified	Respiratory symptoms
**R0600**	Dyspnea, unspecified	Respiratory symptoms
**R0601**	Orthopnea	Respiratory symptoms
**R0602**	Shortness of breath	Respiratory symptoms
**R0603**	Acute respiratory distress	Respiratory symptoms
**R0609**	Other forms of dyspnea	Respiratory symptoms
**R061**	Stridor	Respiratory symptoms
**R062**	Wheezing	Respiratory symptoms
**R064**	Hyperventilation	Respiratory symptoms
**R066**	Hiccough	Respiratory symptoms
**R067**	Sneezing	Respiratory symptoms
**R0681**	Apnea, not elsewhere classified	Respiratory symptoms
**R0682**	Tachypnea, not elsewhere classified	Respiratory symptoms
**R0689**	Other abnormalities of breathing	Respiratory symptoms
**R069**	Unspecified abnormalities of breathing	Respiratory symptoms
**R070**	Pain in throat	Respiratory symptoms
**R071**	Chest pain on breathing	Respiratory symptoms
**R0781**	Pleurodynia	Respiratory symptoms
**R0782**	Intercostal pain	Respiratory symptoms
**R0901**	Asphyxia	Respiratory symptoms
**R0902**	Hypoxemia	Respiratory symptoms
**R093**	Abnormal sputum	Respiratory symptoms
**R0981**	Nasal congestion	Respiratory symptoms
**R0982**	Postnasal drip	Respiratory symptoms
**R072**	pleurodynia	Respiratory symptoms
**R078**	intercostal	Respiratory symptoms
**R079**	other chest pain	Respiratory symptoms
**I2601**	Septic pulmonary embolism with acute cor pulmonale	Acute pulmonary embolism
**I2602**	Saddle embolus of pulmonary artery with acute cor pulmonale	Acute pulmonary embolism
**I2609**	Other pulmonary embolism with acute cor pulmonale	Acute pulmonary embolism
**I2690**	Septic pulmonary embolism without acute cor pulmonale	Acute pulmonary embolism
**I2692**	Saddle embolus of pulmonary artery without acute cor pulmonale	Acute pulmonary embolism
**I2693**	Single subsegmental pulmonary embolism without acute cor pulmonale	Acute pulmonary embolism
**I2694**	Multiple subsegmental pulmonary emboli without acute cor pulmonale	Acute pulmonary embolism
**I2699**	Other pulmonary embolism without acute cor pulmonale	Acute pulmonary embolism
**J96.10**	Chronic respiratory failure unspecified whether with hypoxia or hypercapnia	COPD and Bronchitis
**J96.11**	Chronic respiratory failure WITH HYPOXIA	COPD and Bronchitis
**J96.12**	Chronic respiratory failure WITH HYPERCAPNIA	COPD and Bronchitis
**J20**	Acute bronchitis	COPD and Bronchitis
**J40**	Bronchitis, not specified as acute or chronic	COPD and Bronchitis
**J41**	Simple and mucopurulent chronic bronchitis	COPD and Bronchitis
**J42**	Unspecified chronic bronchitis	COPD and Bronchitis
**J84**	Interstitial lung disease	Interstitial lung disease
**J960**	Acute respiratory failure	Acute respiratory failure
**J81**	Pulmonary edema	Pulmonary edema
**Musculoskeletal system disorders**		
**R531**	Weakness	Malaise and fatigue
**R5381**	Other malaise	Malaise and fatigue
**R5382**	Chronic fatigue, unspecified	Malaise and fatigue
**R5383**	Other fatigue	Malaise and fatigue
**M2550**	Pain in unspecified joint	Musculoskeletal pain

**M25511**	Pain in right shoulder	Musculoskeletal pain
**M25512**	Pain in left shoulder	Musculoskeletal pain
**M25519**	Pain in unspecified shoulder	Musculoskeletal pain
**M25521**	Pain in right elbow	Musculoskeletal pain
**M25522**	Pain in left elbow	Musculoskeletal pain
**M25529**	Pain in unspecified elbow	Musculoskeletal pain
**M25531**	Pain in right wrist	Musculoskeletal pain
**M25532**	Pain in left wrist	Musculoskeletal pain
**M25539**	Pain in unspecified wrist	Musculoskeletal pain
**M25541**	Pain in joints of right hand	Musculoskeletal pain
**M25542**	Pain in joints of left hand	Musculoskeletal pain
**M25549**	Pain in joints of unspecified hand	Musculoskeletal pain
**M25551**	Pain in right hip	Musculoskeletal pain
**M25552**	Pain in left hip	Musculoskeletal pain
**M25559**	Pain in unspecified hip	Musculoskeletal pain
**M25561**	Pain in right knee	Musculoskeletal pain
**M25562**	Pain in left knee	Musculoskeletal pain
**M25569**	Pain in unspecified knee	Musculoskeletal pain
**M25571**	Pain in right ankle and joints of right foot	Musculoskeletal pain
**M25572**	Pain in left ankle and joints of left foot	Musculoskeletal pain
**M25579**	Pain in unspecified ankle and joints of unspecified foot	Musculoskeletal pain
**M2559**	Pain in other specified joint	Musculoskeletal pain
**M2560**	Stiffness of unspecified joint, not elsewhere classified	Musculoskeletal pain
**M25611**	Stiffness of right shoulder, not elsewhere classified	Musculoskeletal pain
**M25612**	Stiffness of left shoulder, not elsewhere classified	Musculoskeletal pain
**M25619**	Stiffness of unspecified shoulder, not elsewhere classified	Musculoskeletal pain
**M25621**	Stiffness of right elbow, not elsewhere classified	Musculoskeletal pain
**M25622**	Stiffness of left elbow, not elsewhere classified	Musculoskeletal pain
**M25629**	Stiffness of unspecified elbow, not elsewhere classified	Musculoskeletal pain
**M25631**	Stiffness of right wrist, not elsewhere classified	Musculoskeletal pain
**M25632**	Stiffness of left wrist, not elsewhere classified	Musculoskeletal pain
**M25639**	Stiffness of unspecified wrist, not elsewhere classified	Musculoskeletal pain
**M25641**	Stiffness of right hand, not elsewhere classified	Musculoskeletal pain
**M25642**	Stiffness of left hand, not elsewhere classified	Musculoskeletal pain
**M25649**	Stiffness of unspecified hand, not elsewhere classified	Musculoskeletal pain
**M25651**	Stiffness of right hip, not elsewhere classified	Musculoskeletal pain
**M25652**	Stiffness of left hip, not elsewhere classified	Musculoskeletal pain
**M25659**	Stiffness of unspecified hip, not elsewhere classified	Musculoskeletal pain
**M25661**	Stiffness of right knee, not elsewhere classified	Musculoskeletal pain
**M25662**	Stiffness of left knee, not elsewhere classified	Musculoskeletal pain
**M25669**	Stiffness of unspecified knee, not elsewhere classified	Musculoskeletal pain
**M25671**	Stiffness of right ankle, not elsewhere classified	Musculoskeletal pain
**M25672**	Stiffness of left ankle, not elsewhere classified	Musculoskeletal pain
**M25673**	Stiffness of unspecified ankle, not elsewhere classified	Musculoskeletal pain
**M25674**	Stiffness of right foot, not elsewhere classified	Musculoskeletal pain
**M25675**	Stiffness of left foot, not elsewhere classified	Musculoskeletal pain
**M25676**	Stiffness of unspecified foot, not elsewhere classified	Musculoskeletal pain
**M2569**	Stiffness of other specified joint, not elsewhere classified	Musculoskeletal pain
**M546**	Pain in thoracic spine	Musculoskeletal pain
**M5481**	Occipital neuralgia	Musculoskeletal pain
**M5489**	Other dorsalgia	Musculoskeletal pain
**M549**	Dorsalgia, unspecified	Musculoskeletal pain
**M791**	Myalgia	Musculoskeletal pain
**M7910**	Myalgia, unspecified site	Musculoskeletal pain
**M7911**	Myalgia of mastication muscle	Musculoskeletal pain
**M7912**	Myalgia of auxiliary muscles, head and neck	Musculoskeletal pain
**M7918**	Myalgia, other site	Musculoskeletal pain
**M79601**	Pain in right arm	Musculoskeletal pain
**M79602**	Pain in left arm	Musculoskeletal pain
**M79603**	Pain in arm, unspecified	Musculoskeletal pain
**M79604**	Pain in right leg	Musculoskeletal pain
**M79605**	Pain in left leg	Musculoskeletal pain
**M79606**	Pain in leg, unspecified	Musculoskeletal pain
**M79609**	Pain in unspecified limb	Musculoskeletal pain
**M79621**	Pain in right upper arm	Musculoskeletal pain
**M79622**	Pain in left upper arm	Musculoskeletal pain
**M79629**	Pain in unspecified upper arm	Musculoskeletal pain
**M79631**	Pain in right forearm	Musculoskeletal pain

**M79632**	Pain in left forearm	Musculoskeletal pain
**M79639**	Pain in unspecified forearm	Musculoskeletal pain
**M79641**	Pain in right hand	Musculoskeletal pain
**M79642**	Pain in left hand	Musculoskeletal pain
**M79643**	Pain in unspecified hand	Musculoskeletal pain
**M79644**	Pain in right finger(s)	Musculoskeletal pain
**M79645**	Pain in left finger(s)	Musculoskeletal pain
**M79646**	Pain in unspecified finger(s)	Musculoskeletal pain
**M79651**	Pain in right thigh	Musculoskeletal pain
**M79652**	Pain in left thigh	Musculoskeletal pain
**M79659**	Pain in unspecified thigh	Musculoskeletal pain
**M79661**	Pain in right lower leg	Musculoskeletal pain
**M79662**	Pain in left lower leg	Musculoskeletal pain
**M79669**	Pain in unspecified lower leg	Musculoskeletal pain
**M79671**	Pain in right foot	Musculoskeletal pain
**M79672**	Pain in left foot	Musculoskeletal pain
**M79673**	Pain in unspecified foot	Musculoskeletal pain
**M79674**	Pain in right toe(s)	Musculoskeletal pain
**M79675**	Pain in left toe(s)	Musculoskeletal pain
**M79676**	Pain in unspecified toe(s)	Musculoskeletal pain
**M60000**	Infective myositis, unspecified right arm	Muscle disorders
**M60001**	Infective myositis, unspecified left arm	Muscle disorders
**M60002**	Infective myositis, unspecified arm	Muscle disorders
**M60003**	Infective myositis, unspecified right leg	Muscle disorders
**M60004**	Infective myositis, unspecified left leg	Muscle disorders
**M60005**	Infective myositis, unspecified leg	Muscle disorders
**M60009**	Infective myositis, unspecified site	Muscle disorders
**M60011**	Infective myositis, right shoulder	Muscle disorders
**M60012**	Infective myositis, left shoulder	Muscle disorders
**M60019**	Infective myositis, unspecified shoulder	Muscle disorders
**M60021**	Infective myositis, right upper arm	Muscle disorders
**M60022**	Infective myositis, left upper arm	Muscle disorders
**M60029**	Infective myositis, unspecified upper arm	Muscle disorders
**M60031**	Infective myositis, right forearm	Muscle disorders
**M60032**	Infective myositis, left forearm	Muscle disorders
**M60039**	Infective myositis, unspecified forearm	Muscle disorders
**M60041**	Infective myositis, right hand	Muscle disorders
**M60042**	Infective myositis, left hand	Muscle disorders
**M60043**	Infective myositis, unspecified hand	Muscle disorders
**M60044**	Infective myositis, right finger(s)	Muscle disorders
**M60045**	Infective myositis, left finger(s)	Muscle disorders
**M60046**	Infective myositis, unspecified finger(s)	Muscle disorders
**M60051**	Infective myositis, right thigh	Muscle disorders
**M60052**	Infective myositis, left thigh	Muscle disorders
**M60059**	Infective myositis, unspecified thigh	Muscle disorders
**M60061**	Infective myositis, right lower leg	Muscle disorders
**M60062**	Infective myositis, left lower leg	Muscle disorders
**M60069**	Infective myositis, unspecified lower leg	Muscle disorders
**M60070**	Infective myositis, right ankle	Muscle disorders
**M60071**	Infective myositis, left ankle	Muscle disorders
**M60072**	Infective myositis, unspecified ankle	Muscle disorders
**M60073**	Infective myositis, right foot	Muscle disorders
**M60074**	Infective myositis, left foot	Muscle disorders
**M60075**	Infective myositis, unspecified foot	Muscle disorders
**M60076**	Infective myositis, right toe(s)	Muscle disorders
**M60077**	Infective myositis, left toe(s)	Muscle disorders
**M60078**	Infective myositis, unspecified toe(s)	Muscle disorders
**M6008**	Infective myositis, other site	Muscle disorders
**M6009**	Infective myositis, multiple sites	Muscle disorders
**M6010**	Interstitial myositis of unspecified site	Muscle disorders
**M60111**	Interstitial myositis, right shoulder	Muscle disorders
**M60112**	Interstitial myositis, left shoulder	Muscle disorders
**M60119**	Interstitial myositis, unspecified shoulder	Muscle disorders
**M60121**	Interstitial myositis, right upper arm	Muscle disorders
**M60122**	Interstitial myositis, left upper arm	Muscle disorders
**M60129**	Interstitial myositis, unspecified upper arm	Muscle disorders
**M60131**	Interstitial myositis, right forearm	Muscle disorders
**M60132**	Interstitial myositis, left forearm	Muscle disorders

**M60139**	Interstitial myositis, unspecified forearm	Muscle disorders
**M60141**	Interstitial myositis, right hand	Muscle disorders
**M60142**	Interstitial myositis, left hand	Muscle disorders
**M60149**	Interstitial myositis, unspecified hand	Muscle disorders
**M60151**	Interstitial myositis, right thigh	Muscle disorders
**M60152**	Interstitial myositis, left thigh	Muscle disorders
**M60159**	Interstitial myositis, unspecified thigh	Muscle disorders
**M60161**	Interstitial myositis, right lower leg	Muscle disorders
**M60162**	Interstitial myositis, left lower leg	Muscle disorders
**M60169**	Interstitial myositis, unspecified lower leg	Muscle disorders
**M60171**	Interstitial myositis, right ankle and foot	Muscle disorders
**M60172**	Interstitial myositis, left ankle and foot	Muscle disorders
**M60179**	Interstitial myositis, unspecified ankle and foot	Muscle disorders
**M6018**	Interstitial myositis, other site	Muscle disorders
**M6019**	Interstitial myositis, multiple sites	Muscle disorders
**M6080**	Other myositis, unspecified site	Muscle disorders
**M60811**	Other myositis, right shoulder	Muscle disorders
**M60812**	Other myositis, left shoulder	Muscle disorders
**M60819**	Other myositis, unspecified shoulder	Muscle disorders
**M60821**	Other myositis, right upper arm	Muscle disorders
**M60822**	Other myositis, left upper arm	Muscle disorders
**M60829**	Other myositis, unspecified upper arm	Muscle disorders
**M60831**	Other myositis, right forearm	Muscle disorders
**M60832**	Other myositis, left forearm	Muscle disorders
**M60839**	Other myositis, unspecified forearm	Muscle disorders
**M60841**	Other myositis, right hand	Muscle disorders
**M60842**	Other myositis, left hand	Muscle disorders
**M60849**	Other myositis, unspecified hand	Muscle disorders
**M60851**	Other myositis, right thigh	Muscle disorders
**M60852**	Other myositis, left thigh	Muscle disorders
**M60859**	Other myositis, unspecified thigh	Muscle disorders
**M60861**	Other myositis, right lower leg	Muscle disorders
**M60862**	Other myositis, left lower leg	Muscle disorders
**M60869**	Other myositis, unspecified lower leg	Muscle disorders
**M60871**	Other myositis, right ankle and foot	Muscle disorders
**M60872**	Other myositis, left ankle and foot	Muscle disorders
**M60879**	Other myositis, unspecified ankle and foot	Muscle disorders
**M6088**	Other myositis, other site	Muscle disorders
**M6089**	Other myositis, multiple sites	Muscle disorders
**M609**	Myositis, unspecified	Muscle disorders
**M6100**	Myositis ossificans traumatica, unspecified site	Muscle disorders
**M61011**	Myositis ossificans traumatica, right shoulder	Muscle disorders
**M61012**	Myositis ossificans traumatica, left shoulder	Muscle disorders
**M61019**	Myositis ossificans traumatica, unspecified shoulder	Muscle disorders
**M61021**	Myositis ossificans traumatica, right upper arm	Muscle disorders
**M61022**	Myositis ossificans traumatica, left upper arm	Muscle disorders
**M61029**	Myositis ossificans traumatica, unspecified upper arm	Muscle disorders
**M61031**	Myositis ossificans traumatica, right forearm	Muscle disorders
**M61032**	Myositis ossificans traumatica, left forearm	Muscle disorders
**M61039**	Myositis ossificans traumatica, unspecified forearm	Muscle disorders
**M61041**	Myositis ossificans traumatica, right hand	Muscle disorders
**M61042**	Myositis ossificans traumatica, left hand	Muscle disorders
**M61049**	Myositis ossificans traumatica, unspecified hand	Muscle disorders
**M61051**	Myositis ossificans traumatica, right thigh	Muscle disorders
**M61052**	Myositis ossificans traumatica, left thigh	Muscle disorders
**M61059**	Myositis ossificans traumatica, unspecified thigh	Muscle disorders
**M61061**	Myositis ossificans traumatica, right lower leg	Muscle disorders
**M61062**	Myositis ossificans traumatica, left lower leg	Muscle disorders
**M61069**	Myositis ossificans traumatica, unspecified lower leg	Muscle disorders
**M61071**	Myositis ossificans traumatica, right ankle and foot	Muscle disorders
**M61072**	Myositis ossificans traumatica, left ankle and foot	Muscle disorders
**M61079**	Myositis ossificans traumatica, unspecified ankle and foot	Muscle disorders
**M6108**	Myositis ossificans traumatica, other site	Muscle disorders
**M6109**	Myositis ossificans traumatica, multiple sites	Muscle disorders
**M6110**	Myositis ossificans progressiva, unspecified site	Muscle disorders
**M61111**	Myositis ossificans progressiva, right shoulder	Muscle disorders
**M61112**	Myositis ossificans progressiva, left shoulder	Muscle disorders
**M61119**	Myositis ossificans progressiva, unspecified shoulder	Muscle disorders

**M61121**	Myositis ossificans progressiva, right upper arm	Muscle disorders
**M61122**	Myositis ossificans progressiva, left upper arm	Muscle disorders
**M61129**	Myositis ossificans progressiva, unspecified arm	Muscle disorders
**M61131**	Myositis ossificans progressiva, right forearm	Muscle disorders
**M61132**	Myositis ossificans progressiva, left forearm	Muscle disorders
**M61139**	Myositis ossificans progressiva, unspecified forearm	Muscle disorders
**M61141**	Myositis ossificans progressiva, right hand	Muscle disorders
**M61142**	Myositis ossificans progressiva, left hand	Muscle disorders
**M61143**	Myositis ossificans progressiva, unspecified hand	Muscle disorders
**M61144**	Myositis ossificans progressiva, right finger(s)	Muscle disorders
**M61145**	Myositis ossificans progressiva, left finger(s)	Muscle disorders
**M61146**	Myositis ossificans progressiva, unspecified finger(s)	Muscle disorders
**M61151**	Myositis ossificans progressiva, right thigh	Muscle disorders
**M61152**	Myositis ossificans progressiva, left thigh	Muscle disorders
**M61159**	Myositis ossificans progressiva, unspecified thigh	Muscle disorders
**M61161**	Myositis ossificans progressiva, right lower leg	Muscle disorders
**M61162**	Myositis ossificans progressiva, left lower leg	Muscle disorders
**M61169**	Myositis ossificans progressiva, unspecified lower leg	Muscle disorders
**M61171**	Myositis ossificans progressiva, right ankle	Muscle disorders
**M61172**	Myositis ossificans progressiva, left ankle	Muscle disorders
**M61173**	Myositis ossificans progressiva, unspecified ankle	Muscle disorders
**M61174**	Myositis ossificans progressiva, right foot	Muscle disorders
**M61175**	Myositis ossificans progressiva, left foot	Muscle disorders
**M61176**	Myositis ossificans progressiva, unspecified foot	Muscle disorders
**M61177**	Myositis ossificans progressiva, right toe(s)	Muscle disorders
**M61178**	Myositis ossificans progressiva, left toe(s)	Muscle disorders
**M61179**	Myositis ossificans progressiva, unspecified toe(s)	Muscle disorders
**M6118**	Myositis ossificans progressiva, other site	Muscle disorders
**M6119**	Myositis ossificans progressiva, multiple sites	Muscle disorders
**M6120**	Paralytic calcification and ossification of muscle, unspecified site	Muscle disorders
**M61211**	Paralytic calcification and ossification of muscle, right shoulder	Muscle disorders
**M61212**	Paralytic calcification and ossification of muscle, left shoulder	Muscle disorders
**M61219**	Paralytic calcification and ossification of muscle, unspecified shoulder	Muscle disorders
**M61221**	Paralytic calcification and ossification of muscle, right upper arm	Muscle disorders
**M61222**	Paralytic calcification and ossification of muscle, left upper arm	Muscle disorders
**M61229**	Paralytic calcification and ossification of muscle, unspecified upper arm	Muscle disorders
**M61231**	Paralytic calcification and ossification of muscle, right forearm	Muscle disorders
**M61232**	Paralytic calcification and ossification of muscle, left forearm	Muscle disorders
**M61239**	Paralytic calcification and ossification of muscle, unspecified forearm	Muscle disorders
**M61241**	Paralytic calcification and ossification of muscle, right hand	Muscle disorders
**M61242**	Paralytic calcification and ossification of muscle, left hand	Muscle disorders
**M61249**	Paralytic calcification and ossification of muscle, unspecified hand	Muscle disorders
**M61251**	Paralytic calcification and ossification of muscle, right thigh	Muscle disorders
**M61252**	Paralytic calcification and ossification of muscle, left thigh	Muscle disorders
**M61259**	Paralytic calcification and ossification of muscle, unspecified thigh	Muscle disorders
**M61261**	Paralytic calcification and ossification of muscle, right lower leg	Muscle disorders
**M61262**	Paralytic calcification and ossification of muscle, left lower leg	Muscle disorders
**M61269**	Paralytic calcification and ossification of muscle, unspecified lower leg	Muscle disorders
**M61271**	Paralytic calcification and ossification of muscle, right ankle and foot	Muscle disorders
**M61272**	Paralytic calcification and ossification of muscle, left ankle and foot	Muscle disorders
**M61279**	Paralytic calcification and ossification of muscle, unspecified ankle and foot	Muscle disorders
**M6128**	Paralytic calcification and ossification of muscle, other site	Muscle disorders
**M6129**	Paralytic calcification and ossification of muscle, multiple sites	Muscle disorders
**M6130**	Calcification and ossification of muscles associated with burns, unspecified site	Muscle disorders
**M61311**	Calcification and ossification of muscles associated with burns, right shoulder	Muscle disorders
**M61312**	Calcification and ossification of muscles associated with burns, left shoulder	Muscle disorders
**M61319**	Calcification and ossification of muscles associated with burns, unspecified shoulder	Muscle disorders
**M61321**	Calcification and ossification of muscles associated with burns, right upper arm	Muscle disorders
**M61322**	Calcification and ossification of muscles associated with burns, left upper arm	Muscle disorders
**M61329**	Calcification and ossification of muscles associated with burns, unspecified upper arm	Muscle disorders
**M61331**	Calcification and ossification of muscles associated with burns, right forearm	Muscle disorders
**M61332**	Calcification and ossification of muscles associated with burns, left forearm	Muscle disorders
**M61339**	Calcification and ossification of muscles associated with burns, unspecified forearm	Muscle disorders
**M61341**	Calcification and ossification of muscles associated with burns, right hand	Muscle disorders
**M61342**	Calcification and ossification of muscles associated with burns, left hand	Muscle disorders
**M61349**	Calcification and ossification of muscles associated with burns, unspecified hand	Muscle disorders
**M61351**	Calcification and ossification of muscles associated with burns, right thigh	Muscle disorders
**M61352**	Calcification and ossification of muscles associated with burns, left thigh	Muscle disorders

**M61359**	Calcification and ossification of muscles associated with burns, unspecified thigh	Muscle disorders
**M61361**	Calcification and ossification of muscles associated with burns, right lower leg	Muscle disorders
**M61362**	Calcification and ossification of muscles associated with burns, left lower leg	Muscle disorders
**M61369**	Calcification and ossification of muscles associated with burns, unspecified lower leg	Muscle disorders
**M61371**	Calcification and ossification of muscles associated with burns, right ankle and	Muscle disorders
**M61372**	Calcification and ossification of muscles associated with burns, left ankle and	Muscle disorders
**M61379**	Calcification and ossification of muscles associated with burns, unspecified ankle and	Muscle disorders
**M6138**	Calcification and ossification of muscles associated with burns, other site	Muscle disorders
**M6139**	Calcification and ossification of muscles associated with burns, multiple sites	Muscle disorders
**M6140**	Other calcification of muscle, unspecified site	Muscle disorders
**M61411**	Other calcification of muscle, right shoulder	Muscle disorders
**M61412**	Other calcification of muscle, left shoulder	Muscle disorders
**M61419**	Other calcification of muscle, unspecified shoulder	Muscle disorders
**M61421**	Other calcification of muscle, right upper arm	Muscle disorders
**M61422**	Other calcification of muscle, left upper arm	Muscle disorders
**M61429**	Other calcification of muscle, unspecified upper arm	Muscle disorders
**M61431**	Other calcification of muscle, right forearm	Muscle disorders
**M61432**	Other calcification of muscle, left forearm	Muscle disorders
**M61439**	Other calcification of muscle, unspecified forearm	Muscle disorders
**M61441**	Other calcification of muscle, right hand	Muscle disorders
**M61442**	Other calcification of muscle, left hand	Muscle disorders
**M61449**	Other calcification of muscle, unspecified hand	Muscle disorders
**M61451**	Other calcification of muscle, right thigh	Muscle disorders
**M61452**	Other calcification of muscle, left thigh	Muscle disorders
**M61459**	Other calcification of muscle, unspecified thigh	Muscle disorders
**M61461**	Other calcification of muscle, right lower leg	Muscle disorders
**M61462**	Other calcification of muscle, left lower leg	Muscle disorders
**M61469**	Other calcification of muscle, unspecified lower leg	Muscle disorders
**M61471**	Other calcification of muscle, right ankle and foot	Muscle disorders
**M61472**	Other calcification of muscle, left ankle and foot	Muscle disorders
**M61479**	Other calcification of muscle, unspecified ankle and foot	Muscle disorders
**M6148**	Other calcification of muscle, other site	Muscle disorders
**M6149**	Other calcification of muscle, multiple sites	Muscle disorders
**M6150**	Other ossification of muscle, unspecified site	Muscle disorders
**M61511**	Other ossification of muscle, right shoulder	Muscle disorders
**M61512**	Other ossification of muscle, left shoulder	Muscle disorders
**M61519**	Other ossification of muscle, unspecified shoulder	Muscle disorders
**M61521**	Other ossification of muscle, right upper arm	Muscle disorders
**M61522**	Other ossification of muscle, left upper arm	Muscle disorders
**M61529**	Other ossification of muscle, unspecified upper arm	Muscle disorders
**M61531**	Other ossification of muscle, right forearm	Muscle disorders
**M61532**	Other ossification of muscle, left forearm	Muscle disorders
**M61539**	Other ossification of muscle, unspecified forearm	Muscle disorders
**M61541**	Other ossification of muscle, right hand	Muscle disorders
**M61542**	Other ossification of muscle, left hand	Muscle disorders
**M61549**	Other ossification of muscle, unspecified hand	Muscle disorders
**M61551**	Other ossification of muscle, right thigh	Muscle disorders
**M61552**	Other ossification of muscle, left thigh	Muscle disorders
**M61559**	Other ossification of muscle, unspecified thigh	Muscle disorders
**M61561**	Other ossification of muscle, right lower leg	Muscle disorders
**M61562**	Other ossification of muscle, left lower leg	Muscle disorders
**M61569**	Other ossification of muscle, unspecified lower leg	Muscle disorders
**M61571**	Other ossification of muscle, right ankle and foot	Muscle disorders
**M61572**	Other ossification of muscle, left ankle and foot	Muscle disorders
**M61579**	Other ossification of muscle, unspecified ankle and foot	Muscle disorders
**M6158**	Other ossification of muscle, other site	Muscle disorders
**M6159**	Other ossification of muscle, multiple sites	Muscle disorders
**M619**	Calcification and ossification of muscle, unspecified	Muscle disorders
**M6200**	Separation of muscle (nontraumatic), unspecified site	Muscle disorders
**M62011**	Separation of muscle (nontraumatic), right shoulder	Muscle disorders
**M62012**	Separation of muscle (nontraumatic), left shoulder	Muscle disorders
**M62019**	Separation of muscle (nontraumatic), unspecified shoulder	Muscle disorders
**M62021**	Separation of muscle (nontraumatic), right upper arm	Muscle disorders
**M62022**	Separation of muscle (nontraumatic), left upper arm	Muscle disorders
**M62029**	Separation of muscle (nontraumatic), unspecified upper arm	Muscle disorders
**M62031**	Separation of muscle (nontraumatic), right forearm	Muscle disorders
**M62032**	Separation of muscle (nontraumatic), left forearm	Muscle disorders
**M62039**	Separation of muscle (nontraumatic), unspecified forearm	Muscle disorders

**M62041**	Separation of muscle (nontraumatic), right hand	Muscle disorders
**M62042**	Separation of muscle (nontraumatic), left hand	Muscle disorders
**M62049**	Separation of muscle (nontraumatic), unspecified hand	Muscle disorders
**M62051**	Separation of muscle (nontraumatic), right thigh	Muscle disorders
**M62052**	Separation of muscle (nontraumatic), left thigh	Muscle disorders
**M62059**	Separation of muscle (nontraumatic), unspecified thigh	Muscle disorders
**M62061**	Separation of muscle (nontraumatic), right lower leg	Muscle disorders
**M62062**	Separation of muscle (nontraumatic), left lower leg	Muscle disorders
**M62069**	Separation of muscle (nontraumatic), unspecified lower leg	Muscle disorders
**M62071**	Separation of muscle (nontraumatic), right ankle and foot	Muscle disorders
**M62072**	Separation of muscle (nontraumatic), left ankle and foot	Muscle disorders
**M62079**	Separation of muscle (nontraumatic), unspecified ankle and foot	Muscle disorders
**M6208**	Separation of muscle (nontraumatic), other site	Muscle disorders
**M6210**	Other rupture of muscle (nontraumatic), unspecified site	Muscle disorders
**M62111**	Other rupture of muscle (nontraumatic), right shoulder	Muscle disorders
**M62112**	Other rupture of muscle (nontraumatic), left shoulder	Muscle disorders
**M62119**	Other rupture of muscle (nontraumatic), unspecified shoulder	Muscle disorders
**M62121**	Other rupture of muscle (nontraumatic), right upper arm	Muscle disorders
**M62122**	Other rupture of muscle (nontraumatic), left upper arm	Muscle disorders
**M62129**	Other rupture of muscle (nontraumatic), unspecified upper arm	Muscle disorders
**M62131**	Other rupture of muscle (nontraumatic), right forearm	Muscle disorders
**M62132**	Other rupture of muscle (nontraumatic), left forearm	Muscle disorders
**M62139**	Other rupture of muscle (nontraumatic), unspecified forearm	Muscle disorders
**M62141**	Other rupture of muscle (nontraumatic), right hand	Muscle disorders
**M62142**	Other rupture of muscle (nontraumatic), left hand	Muscle disorders
**M62149**	Other rupture of muscle (nontraumatic), unspecified hand	Muscle disorders
**M62151**	Other rupture of muscle (nontraumatic), right thigh	Muscle disorders
**M62152**	Other rupture of muscle (nontraumatic), left thigh	Muscle disorders
**M62159**	Other rupture of muscle (nontraumatic), unspecified thigh	Muscle disorders
**M62161**	Other rupture of muscle (nontraumatic), right lower leg	Muscle disorders
**M62162**	Other rupture of muscle (nontraumatic), left lower leg	Muscle disorders
**M62169**	Other rupture of muscle (nontraumatic), unspecified lower leg	Muscle disorders
**M62171**	Other rupture of muscle (nontraumatic), right ankle and foot	Muscle disorders
**M62172**	Other rupture of muscle (nontraumatic), left ankle and foot	Muscle disorders
**M62179**	Other rupture of muscle (nontraumatic), unspecified ankle and foot	Muscle disorders
**M6218**	Other rupture of muscle (nontraumatic), other site	Muscle disorders
**M6220**	Nontraumatic ischemic infarction of muscle, unspecified site	Muscle disorders
**M62211**	Nontraumatic ischemic infarction of muscle, right shoulder	Muscle disorders
**M62212**	Nontraumatic ischemic infarction of muscle, left shoulder	Muscle disorders
**M62219**	Nontraumatic ischemic infarction of muscle, unspecified shoulder	Muscle disorders
**M62221**	Nontraumatic ischemic infarction of muscle, right upper arm	Muscle disorders
**M62222**	Nontraumatic ischemic infarction of muscle, left upper arm	Muscle disorders
**M62229**	Nontraumatic ischemic infarction of muscle, unspecified upper arm	Muscle disorders
**M62231**	Nontraumatic ischemic infarction of muscle, right forearm	Muscle disorders
**M62232**	Nontraumatic ischemic infarction of muscle, left forearm	Muscle disorders
**M62239**	Nontraumatic ischemic infarction of muscle, unspecified forearm	Muscle disorders
**M62241**	Nontraumatic ischemic infarction of muscle, right hand	Muscle disorders
**M62242**	Nontraumatic ischemic infarction of muscle, left hand	Muscle disorders
**M62249**	Nontraumatic ischemic infarction of muscle, unspecified hand	Muscle disorders
**M62251**	Nontraumatic ischemic infarction of muscle, right thigh	Muscle disorders
**M62252**	Nontraumatic ischemic infarction of muscle, left thigh	Muscle disorders
**M62259**	Nontraumatic ischemic infarction of muscle, unspecified thigh	Muscle disorders
**M62261**	Nontraumatic ischemic infarction of muscle, right lower leg	Muscle disorders
**M62262**	Nontraumatic ischemic infarction of muscle, left lower leg	Muscle disorders
**M62269**	Nontraumatic ischemic infarction of muscle, unspecified lower leg	Muscle disorders
**M62271**	Nontraumatic ischemic infarction of muscle, right ankle and foot	Muscle disorders
**M62272**	Nontraumatic ischemic infarction of muscle, left ankle and foot	Muscle disorders
**M62279**	Nontraumatic ischemic infarction of muscle, unspecified ankle and foot	Muscle disorders
**M6228**	Nontraumatic ischemic infarction of muscle, other site	Muscle disorders
**M623**	Immobility syndrome (paraplegic)	Muscle disorders
**M6240**	Contracture of muscle, unspecified site	Muscle disorders
**M62411**	Contracture of muscle, right shoulder	Muscle disorders
**M62412**	Contracture of muscle, left shoulder	Muscle disorders
**M62419**	Contracture of muscle, unspecified shoulder	Muscle disorders
**M62421**	Contracture of muscle, right upper arm	Muscle disorders
**M62422**	Contracture of muscle, left upper arm	Muscle disorders
**M62429**	Contracture of muscle, unspecified upper arm	Muscle disorders
**M62431**	Contracture of muscle, right forearm	Muscle disorders

**M62432**	Contracture of muscle, left forearm	Muscle disorders
**M62439**	Contracture of muscle, unspecified forearm	Muscle disorders
**M62441**	Contracture of muscle, right hand	Muscle disorders
**M62442**	Contracture of muscle, left hand	Muscle disorders
**M62449**	Contracture of muscle, unspecified hand	Muscle disorders
**M62451**	Contracture of muscle, right thigh	Muscle disorders
**M62452**	Contracture of muscle, left thigh	Muscle disorders
**M62459**	Contracture of muscle, unspecified thigh	Muscle disorders
**M62461**	Contracture of muscle, right lower leg	Muscle disorders
**M62462**	Contracture of muscle, left lower leg	Muscle disorders
**M62469**	Contracture of muscle, unspecified lower leg	Muscle disorders
**M62471**	Contracture of muscle, right ankle and foot	Muscle disorders
**M62472**	Contracture of muscle, left ankle and foot	Muscle disorders
**M62479**	Contracture of muscle, unspecified ankle and foot	Muscle disorders
**M6248**	Contracture of muscle, other site	Muscle disorders
**M6249**	Contracture of muscle, multiple sites	Muscle disorders
**M6250**	Muscle wasting and atrophy, not elsewhere classified, unspecified site	Muscle disorders
**M62511**	Muscle wasting and atrophy, not elsewhere classified, right shoulder	Muscle disorders
**M62512**	Muscle wasting and atrophy, not elsewhere classified, left shoulder	Muscle disorders
**M62519**	Muscle wasting and atrophy, not elsewhere classified, unspecified shoulder	Muscle disorders
**M62521**	Muscle wasting and atrophy, not elsewhere classified, right upper arm	Muscle disorders
**M62522**	Muscle wasting and atrophy, not elsewhere classified, left upper arm	Muscle disorders
**M62529**	Muscle wasting and atrophy, not elsewhere classified, unspecified upper arm	Muscle disorders
**M62531**	Muscle wasting and atrophy, not elsewhere classified, right forearm	Muscle disorders
**M62532**	Muscle wasting and atrophy, not elsewhere classified, left forearm	Muscle disorders
**M62539**	Muscle wasting and atrophy, not elsewhere classified, unspecified forearm	Muscle disorders
**M62541**	Muscle wasting and atrophy, not elsewhere classified, right hand	Muscle disorders
**M62542**	Muscle wasting and atrophy, not elsewhere classified, left hand	Muscle disorders
**M62549**	Muscle wasting and atrophy, not elsewhere classified, unspecified hand	Muscle disorders
**M62551**	Muscle wasting and atrophy, not elsewhere classified, right thigh	Muscle disorders
**M62552**	Muscle wasting and atrophy, not elsewhere classified, left thigh	Muscle disorders
**M62559**	Muscle wasting and atrophy, not elsewhere classified, unspecified thigh	Muscle disorders
**M62561**	Muscle wasting and atrophy, not elsewhere classified, right lower leg	Muscle disorders
**M62562**	Muscle wasting and atrophy, not elsewhere classified, left lower leg	Muscle disorders
**M62569**	Muscle wasting and atrophy, not elsewhere classified, unspecified lower leg	Muscle disorders
**M62571**	Muscle wasting and atrophy, not elsewhere classified, right ankle and foot	Muscle disorders
**M62572**	Muscle wasting and atrophy, not elsewhere classified, left ankle and foot	Muscle disorders
**M62579**	Muscle wasting and atrophy, not elsewhere classified, unspecified ankle and foot	Muscle disorders
**M6258**	Muscle wasting and atrophy, not elsewhere classified, other site	Muscle disorders
**M6259**	Muscle wasting and atrophy, not elsewhere classified, multiple sites	Muscle disorders
**M6281**	Muscle weakness (generalized)	Muscle disorders
**M6282**	Rhabdomyolysis	Muscle disorders
**M62831**	Muscle spasm of calf	Muscle disorders
**M62838**	Other muscle spasm	Muscle disorders
**M6284**	Sarcopenia	Muscle disorders
**M6289**	Other specified disorders of muscle	Muscle disorders
**M629**	Disorder of muscle, unspecified	Muscle disorders
**M6380**	Disorders of muscle in diseases classified elsewhere, unspecified site	Muscle disorders
**M63811**	Disorders of muscle in diseases classified elsewhere, right shoulder	Muscle disorders
**M63812**	Disorders of muscle in diseases classified elsewhere, left shoulder	Muscle disorders
**M63819**	Disorders of muscle in diseases classified elsewhere, unspecified shoulder	Muscle disorders
**M63821**	Disorders of muscle in diseases classified elsewhere, right upper arm	Muscle disorders
**M63822**	Disorders of muscle in diseases classified elsewhere, left upper arm	Muscle disorders
**M63829**	Disorders of muscle in diseases classified elsewhere, unspecified upper arm	Muscle disorders
**M63831**	Disorders of muscle in diseases classified elsewhere, right forearm	Muscle disorders
**M63832**	Disorders of muscle in diseases classified elsewhere, left forearm	Muscle disorders
**M63839**	Disorders of muscle in diseases classified elsewhere, unspecified forearm	Muscle disorders
**M63841**	Disorders of muscle in diseases classified elsewhere, right hand	Muscle disorders
**M63842**	Disorders of muscle in diseases classified elsewhere, left hand	Muscle disorders
**M63849**	Disorders of muscle in diseases classified elsewhere, unspecified hand	Muscle disorders
**M63851**	Disorders of muscle in diseases classified elsewhere, right thigh	Muscle disorders
**M63852**	Disorders of muscle in diseases classified elsewhere, left thigh	Muscle disorders
**M63859**	Disorders of muscle in diseases classified elsewhere, unspecified thigh	Muscle disorders
**M63861**	Disorders of muscle in diseases classified elsewhere, right lower leg	Muscle disorders
**M63862**	Disorders of muscle in diseases classified elsewhere, left lower leg	Muscle disorders
**M63869**	Disorders of muscle in diseases classified elsewhere, unspecified lower leg	Muscle disorders
**M63871**	Disorders of muscle in diseases classified elsewhere, right ankle and foot	Muscle disorders
**M63872**	Disorders of muscle in diseases classified elsewhere, left ankle and foot	Muscle disorders

**M63879**	Disorders of muscle in diseases classified elsewhere, unspecified ankle and foot	Muscle disorders
**M6388**	Disorders of muscle in diseases classified elsewhere, other site	Muscle disorders
**M6389**	Disorders of muscle in diseases classified elsewhere, multiple sites	Muscle disorders
Renal system disorders		
**N170**	Acute kidney failure with tubular necrosis	Renal failure
**N171**	Acute kidney failure with acute cortical necrosis	Renal failure
**N172**	Acute kidney failure with medullary necrosis	Renal failure
**N178**	Other acute kidney failure	Renal failure
**N179**	Acute kidney failure, unspecified	Renal failure
**N19**	Unspecified kidney failure	Renal failure
**N181**	Chronic kidney disease, stage 1	Chronic Kidney Disease
**N182**	Chronic kidney disease, stage 2 (mild)	Chronic Kidney Disease
**N183**	Chronic kidney disease, stage 3 (moderate)	Chronic Kidney Disease
**N1830**	Chronic kidney disease, stage 3 unspecified	Chronic Kidney Disease
**N1831**	Chronic kidney disease, stage 3a	Chronic Kidney Disease
**N1832**	Chronic kidney disease, stage 3b	Chronic Kidney Disease
**N184**	Chronic kidney disease, stage 4 (severe)	Chronic Kidney Disease
**N185**	Chronic kidney disease, stage 5	Chronic Kidney Disease
**N186**	End stage renal disease	Chronic Kidney Disease
**N189**	Chronic kidney disease, unspecified	Chronic Kidney Disease
**R880**	Cloudy (hemodialysis) (peritoneal) dialysis effluent	Chronic Kidney Disease
**Gastro-intestinal system disorders**		
**Other**	specified and unspecified gastrointestinal disorders	Gastrointestinal and esophageal
**K580**	Irritable bowel syndrome with diarrhea	Gastrointestinal and esophageal
**K581**	Irritable bowel syndrome with constipation	Gastrointestinal and esophageal
**K582**	Mixed irritable bowel syndrome	Gastrointestinal and esophageal
**K588**	Other irritable bowel syndrome	Gastrointestinal and esophageal
**K589**	Irritable bowel syndrome without diarrhea	Gastrointestinal and esophageal
**K5900**	Constipation, unspecified	Gastrointestinal and esophageal
**K5901**	Slow transit constipation	Gastrointestinal and esophageal
**K5902**	Outlet dysfunction constipation	Gastrointestinal and esophageal
**K5903**	Drug induced constipation	Gastrointestinal and esophageal
**K5904**	Chronic idiopathic constipation	Gastrointestinal and esophageal
**K5909**	Other constipation	Gastrointestinal and esophageal
**K591**	Functional diarrhea	Gastrointestinal and esophageal
**K592**	Neurogenic bowel, not elsewhere classified	Gastrointestinal and esophageal
**K598**	Other specified functional intestinal disorders	Gastrointestinal and esophageal
**K5989**	Other specified functional intestinal disorders	Gastrointestinal and esophageal
**K599**	Functional intestinal disorder, unspecified	Gastrointestinal and esophageal
**K929**	Disease of digestive system, unspecified	Gastrointestinal and esophageal
**K200**	Eosinophilic esophagitis	Gastrointestinal and esophageal
**K208**	Other esophagitis	Gastrointestinal and esophageal
**K2080**	Other esophagitis without bleeding	Gastrointestinal and esophageal
**K2081**	Other esophagitis with bleeding	Gastrointestinal and esophageal
**K209**	Esophagitis, unspecified	Gastrointestinal and esophageal
**K2090**	Esophagitis, unspecified without bleeding	Gastrointestinal and esophageal
**K2091**	Esophagitis, unspecified with bleeding	Gastrointestinal and esophageal
**K210**	Gastro-esophageal reflux disease with esophagitis	Gastrointestinal and esophageal
**K2100**	Gastro-esophageal reflux disease with esophagitis, without bleeding	Gastrointestinal and esophageal
**K2101**	Gastro-esophageal reflux disease with esophagitis, with bleeding	Gastrointestinal and esophageal
**K219**	Gastro-esophageal reflux disease without esophagitis	Gastrointestinal and esophageal
**K220**	Achalasia of cardia	Gastrointestinal and esophageal
**K2210**	Ulcer of esophagus without bleeding	Gastrointestinal and esophageal
**K2211**	Ulcer of esophagus with bleeding	Gastrointestinal and esophageal
**K222**	Esophageal obstruction	Gastrointestinal and esophageal
**K223**	Perforation of esophagus	Gastrointestinal and esophageal
**K224**	Dyskinesia of esophagus	Gastrointestinal and esophageal
**K225**	Diverticulum of esophagus, acquired	Gastrointestinal and esophageal
**K226**	Gastro-esophageal laceration-hemorrhage syndrome	Gastrointestinal and esophageal
**K228**	Other specified diseases of esophagus	Gastrointestinal and esophageal
**K2289**	Other specified disease of esophagus	Gastrointestinal and esophageal
**K229**	Disease of esophagus, unspecified	Gastrointestinal and esophageal
**K23**	Disorders of esophagus in diseases classified elsewhere	Gastrointestinal and esophageal
Neurological disorders		
**G933**	Postviral fatigue syndrome	Neurological conditions
**F05**	Delirium or Encephalopathy	Neurological conditions
**R40.0**	Delirium or Encephalopathy	Neurological conditions

**R41**	Delirium or Encephalopathy	Neurological conditions
**R44**	Delirium or Encephalopathy	Neurological conditions
**F01**	Dementia	Neurological conditions
**F02**	Dementia	Neurological conditions
**F03**	Dementia	Neurological conditions
**G31**	Dementia	Neurological conditions
**A85**	Encephalitis	Neurological conditions
**A86**	Encephalitis	Neurological conditions
**G04**	Encephalitis	Neurological conditions
**G05**	Encephalitis	Neurological conditions
**R29**	Encephalitis	Neurological conditions
**R26**	Ataxia / Trouble walking	Neurological conditions
**R27**	Ataxia / Trouble walking	Neurological conditions
**G26**	Ataxia / Trouble walking	Neurological conditions
**G50**	Peripheral Nerve Disorders	Neurological conditions
**G51**	Peripheral Nerve Disorders	Neurological conditions
**G52**	Peripheral Nerve Disorders	Neurological conditions
**G53**	Peripheral Nerve Disorders	Neurological conditions
**G54**	Peripheral Nerve Disorders	Neurological conditions
**G55**	Peripheral Nerve Disorders	Neurological conditions
**G56**	Peripheral Nerve Disorders	Neurological conditions
**G57**	Peripheral Nerve Disorders	Neurological conditions
**G58**	Peripheral Nerve Disorders	Neurological conditions
**G59**	Peripheral Nerve Disorders	Neurological conditions
**G61**	Peripheral Nerve Disorders	Neurological conditions
**G62**	Peripheral Nerve Disorders	Neurological conditions
**G64**	Peripheral Nerve Disorders	Neurological conditions
**G65**	Peripheral Nerve Disorders	Neurological conditions
**R438**	Other disturbances of smell and taste	Smell and taste disturbances
**R439**	Unspecified disturbances of smell and taste	Smell and taste disturbances
**R43**	Disturbances of smell and taste (All sub-codes)	Smell and taste disturbances
**G40**	Epilepsy and recurrent seizures	Seizures
**H53**	Visual disturbances	Ophthalmologic conditions
**H54**	Visual impairment including blindness (binocular or monocular)	Ophthalmologic conditions
**G21**	Secondary parkinsonism	Parkinsonism
**G24**	Dystonia	Parkinsonism
**G25**	Other extrapyramidal and movement disorders	Parkinsonism
**G90**	Disorders of autonomic nervous system	Autonomic disorders
**G43**	Migraine	Headache
**G44**	Other headache syndromes	Headache
**R51**	Headache	Headache
**G933**	Postviral fatigue syndrome	Neurological conditions
**Skin and subcutaneous system disorders**		
**L209**	Atopic dermatitis	Skin disorders
**L309**	Eczema	Skin disorders
**L501**	Urticaria	Skin disorders
**L508**	Other Urticaria	Skin disorders
**B001**	Herpes viral vesicular dermatitis	Skin disorders
**B09**	Unspecified viral infection characterized by skin and mucous membrane lesions	Skin disorders
**R21**	ash and other nonspecific skin eruption	Skin disorders
**R23**	Cyanosis	Skin disorders
**T691XXA**	chilblains	Skin disorders
**L63**	Alopecia areata	Alopecia
**L65**	Other nonscarring hair loss	Alopecia
**Endocrine system disorders**		
**E1010**	Type 1 diabetes mellitus with ketoacidosis without coma	Type 1 diabetes
**E1011**	Type 1 diabetes mellitus with ketoacidosis with coma	Type 1 diabetes
**E1021**	Type 1 diabetes mellitus with diabetic nephropathy	Type 1 diabetes
**E1022**	Type 1 diabetes mellitus with diabetic chronic kidney disease	Type 1 diabetes
**E1029**	Type 1 diabetes mellitus with other diabetic kidney complication	Type 1 diabetes
**E10311**	Type 1 diabetes mellitus with unspecified diabetic retinopathy with macular edema	Type 1 diabetes
**E10319**	Type 1 diabetes mellitus with unspecified diabetic retinopathy without macular edema	Type 1 diabetes
**E10321**	Type 1 diabetes mellitus with mild nonproliferative diabetic retinopathy with macular	Type 1 diabetes
**E103211**	Type 1 diabetes mellitus with mild nonproliferative diabetic retinopathy with macular	Type 1 diabetes
**E103212**	Type 1 diabetes mellitus with mild nonproliferative diabetic retinopathy with macular	Type 1 diabetes
**E103213**	Type 1 diabetes mellitus with mild nonproliferative diabetic retinopathy with macular	Type 1 diabetes
**E103219**	Type 1 diabetes mellitus with mild nonproliferative diabetic retinopathy with macular	Type 1 diabetes
**E10329**	Type 1 diabetes mellitus with mild nonproliferative diabetic retinopathy without macular	Type 1 diabetes

**E103291**	Type 1 diabetes mellitus with mild nonproliferative diabetic retinopathy without macular	Type 1 diabetes
**E103292**	Type 1 diabetes mellitus with mild nonproliferative diabetic retinopathy without macular	Type 1 diabetes
**E103293**	Type 1 diabetes mellitus with mild nonproliferative diabetic retinopathy without macular	Type 1 diabetes
**E103299**	Type 1 diabetes mellitus with mild nonproliferative diabetic retinopathy without macular	Type 1 diabetes
**E10331**	Type 1 diabetes mellitus with moderate nonproliferative diabetic retinopathy with macular	Type 1 diabetes
**E103311**	Type 1 diabetes mellitus with moderate nonproliferative diabetic retinopathy with macular	Type 1 diabetes
**E103312**	Type 1 diabetes mellitus with moderate nonproliferative diabetic retinopathy with macular	Type 1 diabetes
**E103313**	Type 1 diabetes mellitus with moderate nonproliferative diabetic retinopathy with macular	Type 1 diabetes
**E103319**	Type 1 diabetes mellitus with moderate nonproliferative diabetic retinopathy with macular	Type 1 diabetes
**E10339**	Type 1 diabetes mellitus with moderate nonproliferative diabetic retinopathy without macular	Type 1 diabetes
**E103391**	Type 1 diabetes mellitus with moderate nonproliferative diabetic retinopathy without macular	Type 1 diabetes
**E103392**	Type 1 diabetes mellitus with moderate nonproliferative diabetic retinopathy without macular	Type 1 diabetes
**E103393**	Type 1 diabetes mellitus with moderate nonproliferative diabetic retinopathy without macular	Type 1 diabetes
**E103399**	Type 1 diabetes mellitus with moderate nonproliferative diabetic retinopathy without macular	Type 1 diabetes
**E10341**	Type 1 diabetes mellitus with severe nonproliferative diabetic retinopathy with macular	Type 1 diabetes
**E103411**	Type 1 diabetes mellitus with severe nonproliferative diabetic retinopathy with macular	Type 1 diabetes
**E103412**	Type 1 diabetes mellitus with severe nonproliferative diabetic retinopathy with macular	Type 1 diabetes
**E103413**	Type 1 diabetes mellitus with severe nonproliferative diabetic retinopathy with macular	Type 1 diabetes
**E103419**	Type 1 diabetes mellitus with severe nonproliferative diabetic retinopathy with macular	Type 1 diabetes
**E10349**	Type 1 diabetes mellitus with severe nonproliferative diabetic retinopathy without macular	Type 1 diabetes
**E103491**	Type 1 diabetes mellitus with severe nonproliferative diabetic retinopathy without macular	Type 1 diabetes
**E103492**	Type 1 diabetes mellitus with severe nonproliferative diabetic retinopathy without macular	Type 1 diabetes
**E103493**	Type 1 diabetes mellitus with severe nonproliferative diabetic retinopathy without macular	Type 1 diabetes
**E103499**	Type 1 diabetes mellitus with severe nonproliferative diabetic retinopathy without macular	Type 1 diabetes
**E10351**	Type 1 diabetes mellitus with proliferative diabetic retinopathy with macular edema	Type 1 diabetes
**E103511**	Type 1 diabetes mellitus with proliferative diabetic retinopathy with macular edema,	Type 1 diabetes
**E103512**	Type 1 diabetes mellitus with proliferative diabetic retinopathy with macular edema,	Type 1 diabetes
**E103513**	Type 1 diabetes mellitus with proliferative diabetic retinopathy with macular edema,	Type 1 diabetes
**E103519**	Type 1 diabetes mellitus with proliferative diabetic retinopathy with macular edema,	Type 1 diabetes
**E103521**	Type 1 diabetes mellitus with proliferative diabetic retinopathy with traction retinal	Type 1 diabetes
**E103522**	Type 1 diabetes mellitus with proliferative diabetic retinopathy with traction retinal	Type 1 diabetes
**E103523**	Type 1 diabetes mellitus with proliferative diabetic retinopathy with traction retinal	Type 1 diabetes
**E103529**	Type 1 diabetes mellitus with proliferative diabetic retinopathy with traction retinal	Type 1 diabetes
**E103531**	Type 1 diabetes mellitus with proliferative diabetic retinopathy with traction retinal	Type 1 diabetes
**E103532**	Type 1 diabetes mellitus with proliferative diabetic retinopathy with traction retinal	Type 1 diabetes
**E103533**	Type 1 diabetes mellitus with proliferative diabetic retinopathy with traction retinal	Type 1 diabetes
**E103539**	Type 1 diabetes mellitus with proliferative diabetic retinopathy with traction retinal	Type 1 diabetes
**E103541**	Type 1 diabetes mellitus with proliferative diabetic retinopathy with combined traction	Type 1 diabetes
**E103542**	Type 1 diabetes mellitus with proliferative diabetic retinopathy with combined traction	Type 1 diabetes
**E103543**	Type 1 diabetes mellitus with proliferative diabetic retinopathy with combined traction	Type 1 diabetes
**E103549**	Type 1 diabetes mellitus with proliferative diabetic retinopathy with combined traction	Type 1 diabetes
**E103551**	Type 1 diabetes mellitus with stable proliferative diabetic retinopathy, right eye	Type 1 diabetes
**E103552**	Type 1 diabetes mellitus with stable proliferative diabetic retinopathy, left eye	Type 1 diabetes
**E103553**	Type 1 diabetes mellitus with stable proliferative diabetic retinopathy, bilateral	Type 1 diabetes
**E103559**	Type 1 diabetes mellitus with stable proliferative diabetic retinopathy, unspecified eye	Type 1 diabetes
**E10359**	Type 1 diabetes mellitus with proliferative diabetic retinopathy without macular edema	Type 1 diabetes
**E103591**	Type 1 diabetes mellitus with proliferative diabetic retinopathy without macular edema,	Type 1 diabetes
**E103592**	Type 1 diabetes mellitus with proliferative diabetic retinopathy without macular edema,	Type 1 diabetes
**E103593**	Type 1 diabetes mellitus with proliferative diabetic retinopathy without macular edema,	Type 1 diabetes
**E103599**	Type 1 diabetes mellitus with proliferative diabetic retinopathy without macular edema,	Type 1 diabetes
**E1036**	Type 1 diabetes mellitus with diabetic cataract	Type 1 diabetes
**E1037X1**	Type 1 diabetes mellitus with diabetic macular edema, resolved following treatment,	Type 1 diabetes
**E1037X2**	Type 1 diabetes mellitus with diabetic macular edema, resolved following treatment,	Type 1 diabetes
**E1037X3**	Type 1 diabetes mellitus with diabetic macular edema, resolved following treatment,	Type 1 diabetes
**E1037X9**	Type 1 diabetes mellitus with diabetic macular edema, resolved following treatment,	Type 1 diabetes
**E1039**	Type 1 diabetes mellitus with other diabetic ophthalmic complication	Type 1 diabetes
**E1040**	Type 1 diabetes mellitus with diabetic neuropathy, unspecified	Type 1 diabetes
**E1041**	Type 1 diabetes mellitus with diabetic mononeuropathy	Type 1 diabetes
**E1042**	Type 1 diabetes mellitus with diabetic polyneuropathy	Type 1 diabetes
**E1043**	Type 1 diabetes mellitus with diabetic autonomic (poly)neuropathy	Type 1 diabetes
**E1044**	Type 1 diabetes mellitus with diabetic amyotrophy	Type 1 diabetes
**E1049**	Type 1 diabetes mellitus with other diabetic neurological complication	Type 1 diabetes
**E1051**	Type 1 diabetes mellitus with diabetic peripheral angiopathy without gangrene	Type 1 diabetes
**E1052**	Type 1 diabetes mellitus with diabetic peripheral angiopathy with gangrene	Type 1 diabetes
**E1059**	Type 1 diabetes mellitus with other circulatory complications	Type 1 diabetes
**E10610**	Type 1 diabetes mellitus with diabetic neuropathic arthropathy	Type 1 diabetes
**E10618**	Type 1 diabetes mellitus with other diabetic arthropathy	Type 1 diabetes
**E10620**	Type 1 diabetes mellitus with diabetic dermatitis	Type 1 diabetes

**E10621**	Type 1 diabetes mellitus with foot ulcer	Type 1 diabetes
**E10622**	Type 1 diabetes mellitus with other skin ulcer	Type 1 diabetes
**E10628**	Type 1 diabetes mellitus with other skin complications	Type 1 diabetes
**E10630**	Type 1 diabetes mellitus with periodontal disease	Type 1 diabetes
**E10638**	Type 1 diabetes mellitus with other oral complications	Type 1 diabetes
**E10641**	Type 1 diabetes mellitus with hypoglycemia with coma	Type 1 diabetes
**E10649**	Type 1 diabetes mellitus with hypoglycemia without coma	Type 1 diabetes
**E1065**	Type 1 diabetes mellitus with hyperglycemia	Type 1 diabetes
**E1069**	Type 1 diabetes mellitus with other specified complication	Type 1 diabetes
**E108**	Type 1 diabetes mellitus with unspecified complications	Type 1 diabetes
**E109**	Type 1 diabetes mellitus without complications	Type 1 diabetes
**E1100**	Type 2 diabetes mellitus with hyperosmolarity without nonketotic hyperglycemic-hyperosmolar coma (NKHHC)	Type 2 diabetes
**E1101**	Type 2 diabetes mellitus with hyperosmolarity with coma	Type 2 diabetes
**E1110**	Type 2 diabetes mellitus with ketoacidosis without coma	Type 2 diabetes
**E1111**	Type 2 diabetes mellitus with ketoacidosis with coma	Type 2 diabetes
**E1121**	Type 2 diabetes mellitus with diabetic nephropathy	Type 2 diabetes
**E1122**	Type 2 diabetes mellitus with diabetic chronic kidney disease	Type 2 diabetes
**E1129**	Type 2 diabetes mellitus with other diabetic kidney complication	Type 2 diabetes
**E11311**	Type 2 diabetes mellitus with unspecified diabetic retinopathy with macular edema	Type 2 diabetes
**E11319**	Type 2 diabetes mellitus with unspecified diabetic retinopathy without macular edema	Type 2 diabetes
**E11321**	Type 2 diabetes mellitus with mild nonproliferative diabetic retinopathy with macular	Type 2 diabetes
**E113211**	Type 2 diabetes mellitus with mild nonproliferative diabetic retinopathy with macular	Type 2 diabetes
**E113212**	Type 2 diabetes mellitus with mild nonproliferative diabetic retinopathy with macular	Type 2 diabetes
**E113213**	Type 2 diabetes mellitus with mild nonproliferative diabetic retinopathy with macular	Type 2 diabetes
**E113219**	Type 2 diabetes mellitus with mild nonproliferative diabetic retinopathy with macular	Type 2 diabetes
**E11329**	Type 2 diabetes mellitus with mild nonproliferative diabetic retinopathy without macular	Type 2 diabetes
**E113291**	Type 2 diabetes mellitus with mild nonproliferative diabetic retinopathy without macular	Type 2 diabetes
**E113292**	Type 2 diabetes mellitus with mild nonproliferative diabetic retinopathy without macular	Type 2 diabetes
**E113293**	Type 2 diabetes mellitus with mild nonproliferative diabetic retinopathy without macular	Type 2 diabetes
**E113299**	Type 2 diabetes mellitus with mild nonproliferative diabetic retinopathy without macular	Type 2 diabetes
**E11331**	Type 2 diabetes mellitus with moderate nonproliferative diabetic retinopathy with macular	Type 2 diabetes
**E113311**	Type 2 diabetes mellitus with moderate nonproliferative diabetic retinopathy with macular	Type 2 diabetes
**E113312**	Type 2 diabetes mellitus with moderate nonproliferative diabetic retinopathy with macular	Type 2 diabetes
**E113313**	Type 2 diabetes mellitus with moderate nonproliferative diabetic retinopathy with macular	Type 2 diabetes
**E113319**	Type 2 diabetes mellitus with moderate nonproliferative diabetic retinopathy with macular	Type 2 diabetes
**E11339**	Type 2 diabetes mellitus with moderate nonproliferative diabetic retinopathy without macular	Type 2 diabetes
**E113391**	Type 2 diabetes mellitus with moderate nonproliferative diabetic retinopathy without macular	Type 2 diabetes
**E113392**	Type 2 diabetes mellitus with moderate nonproliferative diabetic retinopathy without macular	Type 2 diabetes
**E113393**	Type 2 diabetes mellitus with moderate nonproliferative diabetic retinopathy without macular	Type 2 diabetes
**E113399**	Type 2 diabetes mellitus with moderate nonproliferative diabetic retinopathy without macular	Type 2 diabetes
**E11341**	Type 2 diabetes mellitus with severe nonproliferative diabetic retinopathy with macular	Type 2 diabetes
**E113411**	Type 2 diabetes mellitus with severe nonproliferative diabetic retinopathy with macular	Type 2 diabetes
**E113412**	Type 2 diabetes mellitus with severe nonproliferative diabetic retinopathy with macular	Type 2 diabetes
**E113413**	Type 2 diabetes mellitus with severe nonproliferative diabetic retinopathy with macular	Type 2 diabetes
**E113419**	Type 2 diabetes mellitus with severe nonproliferative diabetic retinopathy with macular	Type 2 diabetes
**E11349**	Type 2 diabetes mellitus with severe nonproliferative diabetic retinopathy without macular	Type 2 diabetes
**E113491**	Type 2 diabetes mellitus with severe nonproliferative diabetic retinopathy without macular	Type 2 diabetes
**E113492**	Type 2 diabetes mellitus with severe nonproliferative diabetic retinopathy without macular	Type 2 diabetes
**E113493**	Type 2 diabetes mellitus with severe nonproliferative diabetic retinopathy without macular	Type 2 diabetes
**E113499**	Type 2 diabetes mellitus with severe nonproliferative diabetic retinopathy without macular	Type 2 diabetes
**E11351**	Type 2 diabetes mellitus with proliferative diabetic retinopathy with macular edema	Type 2 diabetes
**E113511**	Type 2 diabetes mellitus with proliferative diabetic retinopathy with macular edema,	Type 2 diabetes
**E113512**	Type 2 diabetes mellitus with proliferative diabetic retinopathy with macular edema,	Type 2 diabetes
**E113513**	Type 2 diabetes mellitus with proliferative diabetic retinopathy with macular edema,	Type 2 diabetes
**E113519**	Type 2 diabetes mellitus with proliferative diabetic retinopathy with macular edema,	Type 2 diabetes
**E113521**	Type 2 diabetes mellitus with proliferative diabetic retinopathy with traction retinal	Type 2 diabetes
**E113522**	Type 2 diabetes mellitus with proliferative diabetic retinopathy with traction retinal	Type 2 diabetes
**E113523**	Type 2 diabetes mellitus with proliferative diabetic retinopathy with traction retinal	Type 2 diabetes
**E113529**	Type 2 diabetes mellitus with proliferative diabetic retinopathy with traction retinal	Type 2 diabetes
**E113531**	Type 2 diabetes mellitus with proliferative diabetic retinopathy with traction retinal	Type 2 diabetes
**E113532**	Type 2 diabetes mellitus with proliferative diabetic retinopathy with traction retinal	Type 2 diabetes
**E113533**	Type 2 diabetes mellitus with proliferative diabetic retinopathy with traction retinal	Type 2 diabetes
**E113539**	Type 2 diabetes mellitus with proliferative diabetic retinopathy with traction retinal	Type 2 diabetes
**E113541**	Type 2 diabetes mellitus with proliferative diabetic retinopathy with combined traction	Type 2 diabetes
**E113542**	Type 2 diabetes mellitus with proliferative diabetic retinopathy with combined traction	Type 2 diabetes
**E113543**	Type 2 diabetes mellitus with proliferative diabetic retinopathy with combined traction	Type 2 diabetes
**E113549**	Type 2 diabetes mellitus with proliferative diabetic retinopathy with combined traction	Type 2 diabetes

**E113551**	Type 2 diabetes mellitus with stable proliferative diabetic retinopathy, right eye	Type 2 diabetes
**E113552**	Type 2 diabetes mellitus with stable proliferative diabetic retinopathy, left eye	Type 2 diabetes
**E113553**	Type 2 diabetes mellitus with stable proliferative diabetic retinopathy, bilateral	Type 2 diabetes
**E113559**	Type 2 diabetes mellitus with stable proliferative diabetic retinopathy, unspecified eye	Type 2 diabetes
**E11359**	Type 2 diabetes mellitus with proliferative diabetic retinopathy without macular edema	Type 2 diabetes
**E113591**	Type 2 diabetes mellitus with proliferative diabetic retinopathy without macular edema,	Type 2 diabetes
**E113592**	Type 2 diabetes mellitus with proliferative diabetic retinopathy without macular edema,	Type 2 diabetes
**E113593**	Type 2 diabetes mellitus with proliferative diabetic retinopathy without macular edema,	Type 2 diabetes
**E113599**	Type 2 diabetes mellitus with proliferative diabetic retinopathy without macular edema,	Type 2 diabetes
**E1136**	Type 2 diabetes mellitus with diabetic cataract	Type 2 diabetes
**E1137X1**	Type 2 diabetes mellitus with diabetic macular edema, resolved following treatment,	Type 2 diabetes
**E1137X2**	Type 2 diabetes mellitus with diabetic macular edema, resolved following treatment,	Type 2 diabetes
**E1137X3**	Type 2 diabetes mellitus with diabetic macular edema, resolved following treatment,	Type 2 diabetes
**E1137X9**	Type 2 diabetes mellitus with diabetic macular edema, resolved following treatment,	Type 2 diabetes
**E1139**	Type 2 diabetes mellitus with other diabetic ophthalmic complication	Type 2 diabetes
**E1140**	Type 2 diabetes mellitus with diabetic neuropathy, unspecified	Type 2 diabetes
**E1141**	Type 2 diabetes mellitus with diabetic mononeuropathy	Type 2 diabetes
**E1142**	Type 2 diabetes mellitus with diabetic polyneuropathy	Type 2 diabetes
**E1143**	Type 2 diabetes mellitus with diabetic autonomic (poly)neuropathy	Type 2 diabetes
**E1144**	Type 2 diabetes mellitus with diabetic amyotrophy	Type 2 diabetes
**E1149**	Type 2 diabetes mellitus with other diabetic neurological complication	Type 2 diabetes
**Other**	specified and unspecified circulatory disease	Type 2 diabetes
**E1151**	Type 2 diabetes mellitus with diabetic peripheral angiopathy without gangrene	Type 2 diabetes
**E1152**	Type 2 diabetes mellitus with diabetic peripheral angiopathy with gangrene	Type 2 diabetes
**E1159**	Type 2 diabetes mellitus with other circulatory complications	Type 2 diabetes
**E11610**	Type 2 diabetes mellitus with diabetic neuropathic arthropathy	Type 2 diabetes
**E11618**	Type 2 diabetes mellitus with other diabetic arthropathy	Type 2 diabetes
**E11620**	Type 2 diabetes mellitus with diabetic dermatitis	Type 2 diabetes
**E11621**	Type 2 diabetes mellitus with foot ulcer	Type 2 diabetes
**E11622**	Type 2 diabetes mellitus with other skin ulcer	Type 2 diabetes
**E11628**	Type 2 diabetes mellitus with other skin complications	Type 2 diabetes
**E11630**	Type 2 diabetes mellitus with periodontal disease	Type 2 diabetes
**E11638**	Type 2 diabetes mellitus with other oral complications	Type 2 diabetes
**E11641**	Type 2 diabetes mellitus with hypoglycemia with coma	Type 2 diabetes
**E11649**	Type 2 diabetes mellitus with hypoglycemia without coma	Type 2 diabetes
**E1165**	Type 2 diabetes mellitus with hyperglycemia	Type 2 diabetes
**E1169**	Type 2 diabetes mellitus with other specified complication	Type 2 diabetes
**E118**	Type 2 diabetes mellitus with unspecified complications	Type 2 diabetes
**E119**	Type 2 diabetes mellitus without complications	Type 2 diabetes
**E113292**	Type 2 diabetes mellitus with mild nonproliferative diabetic retinopathy without macular	Type 2 diabetes
**E113293**	Type 2 diabetes mellitus with mild nonproliferative diabetic retinopathy without macular	Type 2 diabetes
**E113299**	Type 2 diabetes mellitus with mild nonproliferative diabetic retinopathy without macular	Type 2 diabetes
**E11331**	Type 2 diabetes mellitus with moderate nonproliferative diabetic retinopathy with macular	Type 2 diabetes
**E113311**	Type 2 diabetes mellitus with moderate nonproliferative diabetic retinopathy with macular	Type 2 diabetes
**E113312**	Type 2 diabetes mellitus with moderate nonproliferative diabetic retinopathy with macular	Type 2 diabetes
**E113313**	Type 2 diabetes mellitus with moderate nonproliferative diabetic retinopathy with macular	Type 2 diabetes
**E113319**	Type 2 diabetes mellitus with moderate nonproliferative diabetic retinopathy with macular	Type 2 diabetes
**E11339**	Type 2 diabetes mellitus with moderate nonproliferative diabetic retinopathy without macular	Type 2 diabetes
**E113391**	Type 2 diabetes mellitus with moderate nonproliferative diabetic retinopathy without macular	Type 2 diabetes
**E113392**	Type 2 diabetes mellitus with moderate nonproliferative diabetic retinopathy without macular	Type 2 diabetes
**E113393**	Type 2 diabetes mellitus with moderate nonproliferative diabetic retinopathy without macular	Type 2 diabetes
**E113399**	Type 2 diabetes mellitus with moderate nonproliferative diabetic retinopathy without macular	Type 2 diabetes
**E11341**	Type 2 diabetes mellitus with severe nonproliferative diabetic retinopathy with macular	Type 2 diabetes
**E113411**	Type 2 diabetes mellitus with severe nonproliferative diabetic retinopathy with macular	Type 2 diabetes
**E113412**	Type 2 diabetes mellitus with severe nonproliferative diabetic retinopathy with macular	Type 2 diabetes
**E113413**	Type 2 diabetes mellitus with severe nonproliferative diabetic retinopathy with macular	Type 2 diabetes
**E113419**	Type 2 diabetes mellitus with severe nonproliferative diabetic retinopathy with macular	Type 2 diabetes
**E11349**	Type 2 diabetes mellitus with severe nonproliferative diabetic retinopathy without macular	Type 2 diabetes
**E113491**	Type 2 diabetes mellitus with severe nonproliferative diabetic retinopathy without macular	Type 2 diabetes
**E113492**	Type 2 diabetes mellitus with severe nonproliferative diabetic retinopathy without macular	Type 2 diabetes
**E113493**	Type 2 diabetes mellitus with severe nonproliferative diabetic retinopathy without macular	Type 2 diabetes
**E113499**	Type 2 diabetes mellitus with severe nonproliferative diabetic retinopathy without macular	Type 2 diabetes
**E11351**	Type 2 diabetes mellitus with proliferative diabetic retinopathy with macular edema	Type 2 diabetes
**E113511**	Type 2 diabetes mellitus with proliferative diabetic retinopathy with macular edema,	Type 2 diabetes
**E113512**	Type 2 diabetes mellitus with proliferative diabetic retinopathy with macular edema,	Type 2 diabetes
**E113513**	Type 2 diabetes mellitus with proliferative diabetic retinopathy with macular edema,	Type 2 diabetes
**E113519**	Type 2 diabetes mellitus with proliferative diabetic retinopathy with macular edema,	Type 2 diabetes
**E113521**	Type 2 diabetes mellitus with proliferative diabetic retinopathy with traction retinal	Type 2 diabetes

**E113522**	Type 2 diabetes mellitus with proliferative diabetic retinopathy with traction retinal	Type 2 diabetes
**E113523**	Type 2 diabetes mellitus with proliferative diabetic retinopathy with traction retinal	Type 2 diabetes
**E113529**	Type 2 diabetes mellitus with proliferative diabetic retinopathy with traction retinal	Type 2 diabetes
**E113531**	Type 2 diabetes mellitus with proliferative diabetic retinopathy with traction retinal	Type 2 diabetes
**E113532**	Type 2 diabetes mellitus with proliferative diabetic retinopathy with traction retinal	Type 2 diabetes
**E113533**	Type 2 diabetes mellitus with proliferative diabetic retinopathy with traction retinal	Type 2 diabetes
**E113539**	Type 2 diabetes mellitus with proliferative diabetic retinopathy with traction retinal	Type 2 diabetes
**E113541**	Type 2 diabetes mellitus with proliferative diabetic retinopathy with combined traction	Type 2 diabetes
**E113542**	Type 2 diabetes mellitus with proliferative diabetic retinopathy with combined traction	Type 2 diabetes
**E113543**	Type 2 diabetes mellitus with proliferative diabetic retinopathy with combined traction	Type 2 diabetes
**E113549**	Type 2 diabetes mellitus with proliferative diabetic retinopathy with combined traction	Type 2 diabetes
**E113551**	Type 2 diabetes mellitus with stable proliferative diabetic retinopathy, right eye	Type 2 diabetes
**E113552**	Type 2 diabetes mellitus with stable proliferative diabetic retinopathy, left eye	Type 2 diabetes
**E113553**	Type 2 diabetes mellitus with stable proliferative diabetic retinopathy, bilateral	Type 2 diabetes
**E113559**	Type 2 diabetes mellitus with stable proliferative diabetic retinopathy, unspecified eye	Type 2 diabetes
**E11359**	Type 2 diabetes mellitus with proliferative diabetic retinopathy without macular edema	Type 2 diabetes
**E113591**	Type 2 diabetes mellitus with proliferative diabetic retinopathy without macular edema,	Type 2 diabetes
**E113592**	Type 2 diabetes mellitus with proliferative diabetic retinopathy without macular edema,	Type 2 diabetes
**E113593**	Type 2 diabetes mellitus with proliferative diabetic retinopathy without macular edema,	Type 2 diabetes
**E113599**	Type 2 diabetes mellitus with proliferative diabetic retinopathy without macular edema,	Type 2 diabetes
**E1136**	Type 2 diabetes mellitus with diabetic cataract	Type 2 diabetes
**E1137X1**	Type 2 diabetes mellitus with diabetic macular edema, resolved following treatment,	Type 2 diabetes
**E1137X2**	Type 2 diabetes mellitus with diabetic macular edema, resolved following treatment,	Type 2 diabetes
**E1137X3**	Type 2 diabetes mellitus with diabetic macular edema, resolved following treatment,	Type 2 diabetes
**E1137X9**	Type 2 diabetes mellitus with diabetic macular edema, resolved following treatment,	Type 2 diabetes
**E1139**	Type 2 diabetes mellitus with other diabetic ophthalmic complication	Type 2 diabetes
**E1140**	Type 2 diabetes mellitus with diabetic neuropathy, unspecified	Type 2 diabetes
**E1141**	Type 2 diabetes mellitus with diabetic mononeuropathy	Type 2 diabetes
**E1142**	Type 2 diabetes mellitus with diabetic polyneuropathy	Type 2 diabetes
**E1143**	Type 2 diabetes mellitus with diabetic autonomic (poly)neuropathy	Type 2 diabetes
**E1144**	Type 2 diabetes mellitus with diabetic amyotrophy	Type 2 diabetes
**E1149**	Type 2 diabetes mellitus with other diabetic neurological complication	Type 2 diabetes
**E1151**	Type 2 diabetes mellitus with diabetic peripheral angiopathy without gangrene	Type 2 diabetes
**E1152**	Type 2 diabetes mellitus with diabetic peripheral angiopathy with gangrene	Type 2 diabetes
**E1159**	Type 2 diabetes mellitus with other circulatory complications	Type 2 diabetes
**E11610**	Type 2 diabetes mellitus with diabetic neuropathic arthropathy	Type 2 diabetes
**E11618**	Type 2 diabetes mellitus with other diabetic arthropathy	Type 2 diabetes
**E11620**	Type 2 diabetes mellitus with diabetic dermatitis	Type 2 diabetes
**E11621**	Type 2 diabetes mellitus with foot ulcer	Type 2 diabetes
**E11622**	Type 2 diabetes mellitus with other skin ulcer	Type 2 diabetes
**E11628**	Type 2 diabetes mellitus with other skin complications	Type 2 diabetes
**E11630**	Type 2 diabetes mellitus with periodontal disease	Type 2 diabetes
**E11638**	Type 2 diabetes mellitus with other oral complications	Type 2 diabetes
**E11641**	Type 2 diabetes mellitus with hypoglycemia with coma	Type 2 diabetes
**E11649**	Type 2 diabetes mellitus with hypoglycemia without coma	Type 2 diabetes
**E1165**	Type 2 diabetes mellitus with hyperglycemia	Type 2 diabetes
**E1169**	Type 2 diabetes mellitus with other specified complication	Type 2 diabetes
**E118**	Type 2 diabetes mellitus with unspecified complications	Type 2 diabetes
**E119**	Type 2 diabetes mellitus without complications	Type 2 diabetes
**E113513**	Type 2 diabetes mellitus with proliferative diabetic retinopathy with macular edema,	Type 2 diabetes
**E113519**	Type 2 diabetes mellitus with proliferative diabetic retinopathy with macular edema,	Type 2 diabetes
**E113521**	Type 2 diabetes mellitus with proliferative diabetic retinopathy with traction retinal	Type 2 diabetes
**E113522**	Type 2 diabetes mellitus with proliferative diabetic retinopathy with traction retinal	Type 2 diabetes
**E113523**	Type 2 diabetes mellitus with proliferative diabetic retinopathy with traction retinal	Type 2 diabetes
**E113529**	Type 2 diabetes mellitus with proliferative diabetic retinopathy with traction retinal	Type 2 diabetes
**E113531**	Type 2 diabetes mellitus with proliferative diabetic retinopathy with traction retinal	Type 2 diabetes
**E113532**	Type 2 diabetes mellitus with proliferative diabetic retinopathy with traction retinal	Type 2 diabetes
**E113533**	Type 2 diabetes mellitus with proliferative diabetic retinopathy with traction retinal	Type 2 diabetes
**E113539**	Type 2 diabetes mellitus with proliferative diabetic retinopathy with traction retinal	Type 2 diabetes
**E113541**	Type 2 diabetes mellitus with proliferative diabetic retinopathy with combined traction	Type 2 diabetes
**E113542**	Type 2 diabetes mellitus with proliferative diabetic retinopathy with combined traction	Type 2 diabetes
**E113543**	Type 2 diabetes mellitus with proliferative diabetic retinopathy with combined traction	Type 2 diabetes
**E113549**	Type 2 diabetes mellitus with proliferative diabetic retinopathy with combined traction	Type 2 diabetes
**E113551**	Type 2 diabetes mellitus with stable proliferative diabetic retinopathy, right eye	Type 2 diabetes
**E113552**	Type 2 diabetes mellitus with stable proliferative diabetic retinopathy, left eye	Type 2 diabetes
**E113553**	Type 2 diabetes mellitus with stable proliferative diabetic retinopathy, bilateral	Type 2 diabetes
**E113559**	Type 2 diabetes mellitus with stable proliferative diabetic retinopathy, unspecified eye	Type 2 diabetes
**E11359**	Type 2 diabetes mellitus with proliferative diabetic retinopathy without macular edema	Type 2 diabetes

**E113591**	Type 2 diabetes mellitus with proliferative diabetic retinopathy without macular edema,	Type 2 diabetes
**E113592**	Type 2 diabetes mellitus with proliferative diabetic retinopathy without macular edema,	Type 2 diabetes
**E113593**	Type 2 diabetes mellitus with proliferative diabetic retinopathy without macular edema,	Type 2 diabetes
**E113599**	Type 2 diabetes mellitus with proliferative diabetic retinopathy without macular edema,	Type 2 diabetes
**E1136**	Type 2 diabetes mellitus with diabetic cataract	Type 2 diabetes
**E1137X1**	Type 2 diabetes mellitus with diabetic macular edema, resolved following treatment,	Type 2 diabetes
**E1137X2**	Type 2 diabetes mellitus with diabetic macular edema, resolved following treatment,	Type 2 diabetes
**E1137X3**	Type 2 diabetes mellitus with diabetic macular edema, resolved following treatment,	Type 2 diabetes
**E1137X9**	Type 2 diabetes mellitus with diabetic macular edema, resolved following treatment,	Type 2 diabetes
**E1139**	Type 2 diabetes mellitus with other diabetic ophthalmic complication	Type 2 diabetes
**E1140**	Type 2 diabetes mellitus with diabetic neuropathy, unspecified	Type 2 diabetes
**E1141**	Type 2 diabetes mellitus with diabetic mononeuropathy	Type 2 diabetes
**E1142**	Type 2 diabetes mellitus with diabetic polyneuropathy	Type 2 diabetes
**E1143**	Type 2 diabetes mellitus with diabetic autonomic (poly)neuropathy	Type 2 diabetes
**E1144**	Type 2 diabetes mellitus with diabetic amyotrophy	Type 2 diabetes
**E1149**	Type 2 diabetes mellitus with other diabetic neurological complication	Type 2 diabetes
**Other**	specified and unspecified circulatory disease	Type 2 diabetes
**E1151**	Type 2 diabetes mellitus with diabetic peripheral angiopathy without gangrene	Type 2 diabetes
**E1152**	Type 2 diabetes mellitus with diabetic peripheral angiopathy with gangrene	Type 2 diabetes
**E1159**	Type 2 diabetes mellitus with other circulatory complications	Type 2 diabetes
**E11610**	Type 2 diabetes mellitus with diabetic neuropathic arthropathy	Type 2 diabetes
**E11618**	Type 2 diabetes mellitus with other diabetic arthropathy	Type 2 diabetes
**E11620**	Type 2 diabetes mellitus with diabetic dermatitis	Type 2 diabetes
**E11621**	Type 2 diabetes mellitus with foot ulcer	Type 2 diabetes
**E11622**	Type 2 diabetes mellitus with other skin ulcer	Type 2 diabetes
**E11628**	Type 2 diabetes mellitus with other skin complications	Type 2 diabetes
**E11630**	Type 2 diabetes mellitus with periodontal disease	Type 2 diabetes
**E11638**	Type 2 diabetes mellitus with other oral complications	Type 2 diabetes
**E11641**	Type 2 diabetes mellitus with hypoglycemia with coma	Type 2 diabetes
**E11649**	Type 2 diabetes mellitus with hypoglycemia without coma	Type 2 diabetes
**E1165**	Type 2 diabetes mellitus with hyperglycemia	Type 2 diabetes
**E1169**	Type 2 diabetes mellitus with other specified complication	Type 2 diabetes
**E118**	Type 2 diabetes mellitus with unspecified complications	Type 2 diabetes
**E119**	Type 2 diabetes mellitus without complications	Type 2 diabetes
**Mental and behavioral disorders**		
**F064**	Anxiety disorder due to known physiological condition	Anxiety and fear-related
**F4000**	Agoraphobia, unspecified	Anxiety and fear-related
**F4001**	Agoraphobia with panic disorder	Anxiety and fear-related
**F4002**	Agoraphobia without panic disorder	Anxiety and fear-related
**F4010**	Social phobia, unspecified	Anxiety and fear-related
**F4011**	Social phobia, generalized	Anxiety and fear-related
**F40228**	Other natural environment type phobia	Anxiety and fear-related
**F40230**	Fear of blood	Anxiety and fear-related
**F40231**	Fear of injections and transfusions	Anxiety and fear-related
**F40232**	Fear of other medical care	Anxiety and fear-related
**F40233**	Fear of injury	Anxiety and fear-related
**F40240**	Claustrophobia	Anxiety and fear-related
**F40248**	Other situational type phobia	Anxiety and fear-related
**F408**	Other phobic anxiety disorders	Anxiety and fear-related
**F409**	Phobic anxiety disorder, unspecified	Anxiety and fear-related
**F410**	Panic disorder [episodic paroxysmal anxiety]	Anxiety and fear-related
**F411**	Generalized anxiety disorder	Anxiety and fear-related
**F413**	Other mixed anxiety disorders	Anxiety and fear-related
**F418**	Other specified anxiety disorders	Anxiety and fear-related
**F419**	Anxiety disorder, unspecified	Anxiety and fear-related
**F930**	Separation anxiety disorder of childhood	Anxiety and fear-related
**F431**	PTSD	Anxiety and fear-related
**F304**	Manic episode in full remission	Mood disorders
**F3170**	Bipolar disorder, currently in remission, most recent episode unspecified	Mood disorders
**F3172**	Bipolar disorder, in full remission, most recent episode hypomanic	Mood disorders
**F3174**	Bipolar disorder, in full remission, most recent episode manic	Mood disorders
**F3176**	Bipolar disorder, in full remission, most recent episode depressed	Mood disorders
**F3178**	Bipolar disorder, in full remission, most recent episode mixed	Mood disorders
**F325**	Major depressive disorder, single episode, in full remission	Mood disorders
**F3340**	Major depressive disorder, recurrent, in remission, unspecified	Mood disorders
**F3342**	Major depressive disorder, recurrent, in full remission	Mood disorders
**Other**	mental conditions and symptoms	Mood disorders
**R450**	Nervousness	Mood disorders

**R451**	Restlessness and agitation	Mood disorders
**R452**	Unhappiness	Mood disorders
**R453**	Demoralization and apathy	Mood disorders
**R454**	Irritability and anger	Mood disorders
**R455**	Hostility	Mood disorders
**R456**	Violent behavior	Mood disorders
**R457**	State of emotional shock and stress, unspecified	Mood disorders
**R4581**	Low self-esteem	Mood disorders
**R4582**	Worries	Mood disorders
**R4583**	Excessive crying of child, adolescent or adult	Mood disorders
**R4584**	Anhedonia	Mood disorders
**R45850**	Homicidal ideations	Mood disorders
**R4586**	Emotional lability	Mood disorders
**R4587**	Impulsiveness	Mood disorders
**R4589**	Other symptoms and signs involving emotional state	Mood disorders
**R460**	Very low level of personal hygiene	Mood disorders
**R461**	Bizarre personal appearance	Mood disorders
**R462**	Strange and inexplicable behavior	Mood disorders
**R463**	Overactivity	Mood disorders
**R464**	Slowness and poor responsiveness	Mood disorders
**R465**	Suspiciousness and marked evasiveness	Mood disorders
**R466**	Undue concern and preoccupation with stressful events	Mood disorders
**R467**	Verbosity and circumstantial detail obscuring reason for contact	Mood disorders
**R4681**	Obsessive-compulsive behavior	Mood disorders
**R4689**	Other symptoms and signs involving appearance and behavior	Mood disorders
**Other**	specified and unspecified mood disorders	Mood disorders
**F0630**	Mood disorder due to known physiological condition, unspecified	Mood disorders
**F348**	Other persistent mood [affective] disorders	Mood disorders
**F3481**	Disruptive mood dysregulation disorder	Mood disorders
**F3489**	Other specified persistent mood disorders	Mood disorders
**F349**	Persistent mood [affective] disorder, unspecified	Mood disorders
**F39**	Unspecified mood [affective] disorder	Mood disorders
**F32**	depressive episode	Mood disorders
**F33**	major depressive disorder	Mood disorders
**F34**	persistent mood disorder	Mood disorders
**G4700**	Insomnia, unspecified	Sleep disorders
**G4701**	Insomnia due to medical condition	Sleep disorders
**G4709**	Other insomnia	Sleep disorders
**G4710**	Hypersomnia, unspecified	Sleep disorders
**G4711**	Idiopathic hypersomnia with long sleep time	Sleep disorders
**G4712**	Idiopathic hypersomnia without long sleep time	Sleep disorders
**G4713**	Recurrent hypersomnia	Sleep disorders
**G4714**	Hypersomnia due to medical condition	Sleep disorders
**G4719**	Other hypersomnia	Sleep disorders
**G4720**	Circadian rhythm sleep disorder, unspecified type	Sleep disorders
**G4721**	Circadian rhythm sleep disorder, delayed sleep phase type	Sleep disorders
**G4722**	Circadian rhythm sleep disorder, advanced sleep phase type	Sleep disorders
**G4723**	Circadian rhythm sleep disorder, irregular sleep wake type	Sleep disorders
**G4724**	Circadian rhythm sleep disorder, free running type	Sleep disorders
**G4725**	Circadian rhythm sleep disorder, jet lag type	Sleep disorders
**G4726**	Circadian rhythm sleep disorder, shift work type	Sleep disorders
**G4727**	Circadian rhythm sleep disorder in conditions classified elsewhere	Sleep disorders
**G4729**	Other circadian rhythm sleep disorder	Sleep disorders
**G4730**	Sleep apnea, unspecified	Sleep disorders
**G4731**	Primary central sleep apnea	Sleep disorders
**G4732**	High altitude periodic breathing	Sleep disorders
**G4733**	Obstructive sleep apnea (adult) (pediatric)	Sleep disorders
**G4734**	Idiopathic sleep related nonobstructive alveolar hypoventilation	Sleep disorders
**G4735**	Congenital central alveolar hypoventilation syndrome	Sleep disorders
**G4736**	Sleep related hypoventilation in conditions classified elsewhere	Sleep disorders
**G4737**	Central sleep apnea in conditions classified elsewhere	Sleep disorders
**G4739**	Other sleep apnea	Sleep disorders
**G47411**	Narcolepsy with cataplexy	Sleep disorders
**G47419**	Narcolepsy without cataplexy	Sleep disorders
**G47421**	Narcolepsy in conditions classified elsewhere with cataplexy	Sleep disorders
**G47429**	Narcolepsy in conditions classified elsewhere without cataplexy	Sleep disorders
**G4750**	Parasomnia, unspecified	Sleep disorders
**G4751**	Confusional arousals	Sleep disorders

**G4752**	REM sleep behavior disorder	Sleep disorders
**G4753**	Recurrent isolated sleep paralysis	Sleep disorders
**G4754**	Parasomnia in conditions classified elsewhere	Sleep disorders
**G4759**	Other parasomnia	Sleep disorders
**G4761**	Periodic limb movement disorder	Sleep disorders
**G4762**	Sleep related leg cramps	Sleep disorders
**G4763**	Sleep related bruxism	Sleep disorders
**G4769**	Other sleep related movement disorders	Sleep disorders
**G478**	Other sleep disorders	Sleep disorders
**G479**	Sleep disorder, unspecified	Sleep disorders
**R063**	Periodic breathing	Sleep disorders
**F1011**	Alcohol abuse, in remission	substance use disorders
**F1021**	Alcohol dependence, in remission	substance use disorders
**F1111**	Opioid abuse, in remission	substance use disorders
**F1121**	Opioid dependence, in remission	substance use disorders
**F1211**	Cannabis abuse, in remission	substance use disorders
**F1221**	Cannabis dependence, in remission	substance use disorders
**F1311**	Sedative, hypnotic or anxiolytic abuse, in remission	substance use disorders
**F1321**	Sedative, hypnotic or anxiolytic dependence, in remission	substance use disorders
**F1411**	Cocaine abuse, in remission	substance use disorders
**F1421**	Cocaine dependence, in remission	substance use disorders
**F1511**	Other stimulant abuse, in remission	substance use disorders
**F1521**	Other stimulant dependence, in remission	substance use disorders
**F1611**	Hallucinogen abuse, in remission	substance use disorders
**F1621**	Hallucinogen dependence, in remission	substance use disorders
**F17201**	Nicotine dependence, unspecified, in remission	substance use disorders
**F17211**	Nicotine dependence, cigarettes, in remission	substance use disorders
**F17221**	Nicotine dependence, chewing tobacco, in remission	substance use disorders
**F17291**	Nicotine dependence, other tobacco product, in remission	substance use disorders
**F1811**	Inhalant abuse, in remission	substance use disorders
**F1821**	Inhalant dependence, in remission	substance use disorders
**F1910**	Other psychoactive substance abuse, uncomplicated	substance use disorders
**F1911**	Other psychoactive substance abuse, in remission	substance use disorders
**F19120**	Other psychoactive substance abuse with intoxication, uncomplicated	substance use disorders
**F19121**	Other psychoactive substance abuse with intoxication delirium	substance use disorders
**F19122**	Other psychoactive substance abuse with intoxication with perceptual disturbances	substance use disorders
**F19129**	Other psychoactive substance abuse with intoxication, unspecified	substance use disorders
**F19130**	Other psychoactive substance abuse with withdrawal, uncomplicated	substance use disorders
**F19131**	Other psychoactive substance abuse with withdrawal delirium	substance use disorders
**F19132**	Other psychoactive substance abuse with withdrawal with perceptual disturbance	substance use disorders
**F19139**	Other psychoactive substance abuse with withdrawal, unspecified	substance use disorders
**F1914**	Other psychoactive substance abuse with psychoactive substance-induced mood disorder	substance use disorders
**F1916**	Other psychoactive substance abuse with psychoactive substance-induced persisting amnestic disorder	substance use disorders
**F1917**	Other psychoactive substance abuse with psychoactive substance-induced persisting dementia	substance use disorders
**F19181**	Other psychoactive substance abuse with psychoactive substance-induced sexual dysfunction	substance use disorders
**F19182**	Other psychoactive substance abuse with psychoactive substance-induced sleep disorder	substance use disorders
**F19188**	Other psychoactive substance abuse with other psychoactive substance-induced disorder	substance use disorders
**F1919**	Other psychoactive substance abuse with unspecified psychoactive substance-induced disorder	substance use disorders
**F1920**	Other psychoactive substance dependence, uncomplicated	substance use disorders
**F1921**	Other psychoactive substance dependence, in remission	substance use disorders
**F19220**	Other psychoactive substance dependence with intoxication, uncomplicated	substance use disorders
**F19221**	Other psychoactive substance dependence with intoxication delirium	substance use disorders
**F19222**	Other psychoactive substance dependence with intoxication with perceptual disturbance	substance use disorders
**F19229**	Other psychoactive substance dependence with intoxication, unspecified	substance use disorders
**F19230**	Other psychoactive substance dependence with withdrawal, uncomplicated	substance use disorders
**F19231**	Other psychoactive substance dependence with withdrawal delirium	substance use disorders
**F19232**	Other psychoactive substance dependence with withdrawal with perceptual disturbance	substance use disorders
**F19239**	Other psychoactive substance dependence with withdrawal, unspecified	substance use disorders
**F1924**	Other psychoactive substance dependence with psychoactive substance-induced mood disorder	substance use disorders
**F1926**	Other psychoactive substance dependence with psychoactive substance-induced persisting amnestic disorder	substance use disorders
**F1927**	Other psychoactive substance dependence with psychoactive substance-induced persisting dementia	substance use disorders
**F19281**	Other psychoactive substance dependence with psychoactive substance-induced sexual dysfunction	substance use disorders
**F19282**	Other psychoactive substance dependence with psychoactive substance-induced sleep disorder	substance use disorders
**F19288**	Other psychoactive substance dependence with other psychoactive substance-induced disorder	substance use disorders

**F1929**	Other psychoactive substance dependence with unspecified psychoactive substance-induced disorder	substance use disorders
**F1990**	Other psychoactive substance use, unspecified, uncomplicated	substance use disorders
**F19920**	Other psychoactive substance use, unspecified with intoxication, uncomplicated	substance use disorders
**F19921**	Other psychoactive substance use, unspecified with intoxication with delirium	substance use disorders
**F19922**	Other psychoactive substance use, unspecified with intoxication with perceptual disturbance	substance use disorders
**F19929**	Other psychoactive substance use, unspecified with intoxication, unspecified	substance use disorders
**F19930**	Other psychoactive substance use, unspecified with withdrawal, uncomplicated	substance use disorders
**F19931**	Other psychoactive substance use, unspecified with withdrawal delirium	substance use disorders
**F19932**	Other psychoactive substance use, unspecified with withdrawal with perceptual disturbance	substance use disorders
**F19939**	Other psychoactive substance use, unspecified with withdrawal, unspecified	substance use disorders
**F1994**	Other psychoactive substance use, unspecified with psychoactive substance-induced mood disorder	substance use disorders
**F1996**	Other psychoactive substance use, unspecified with psychoactive substance-induced persisting amnestic disorder	substance use disorders
**F1997**	Other psychoactive substance use, unspecified with psychoactive substance-induced persisting dementia	substance use disorders
**F19981**	Other psychoactive substance use, unspecified with psychoactive substance-induced sexual dysfunction	substance use disorders
**F19982**	Other psychoactive substance use, unspecified with psychoactive substance-induced sleep disorder	substance use disorders
**F19988**	Other psychoactive substance use, unspecified with other psychoactive substance-induced disorder	substance use disorders
**F1999**	Other psychoactive substance use, unspecified with unspecified psychoactive substance-induced disorder	substance use disorders
