# Supplementary material for: Comparative risk of post-acute sequelae following SARS-CoV-2 or influenza virus infection: A retrospective cohort study among United States adults
Source: PLoS Med. 2025 Oct 9;22(10):e1004777. doi: 10.1371/journal.pmed.1004777 (PMC12551960; doi:10.1371/journal.pmed.1004777)
Supplement: S1 Checklist — (PDF) [file pmed.1004777.s003.pdf]

STROBE Statement—checklist of items that should be included in reports of observational studies

	Item No.	Recommendation	Page No.	Relevant text from manuscript
**Title and abstract**	1	(*a*) Indicate the study's design with a commonly used term in the title or the abstract	Title page, title line	Comparative risk of post-acute sequelae among adults following SARS-CoV-2 or influenza virus infection: a retrospective cohort study in a large, integrated healthcare system
		(*b*) Provide in the abstract an informative and balanced summary of what was done and what was found	Abstract, paragraph 2	We undertook a retrospective cohort study of 74,738 COVID-19 cases and 18,790 influenza cases within the Kaiser Permanente Southern California healthcare system diagnosed between 1 September, 2022 and 31 December, 2023. Cases received care for index infections across a spectrum of clinical settings, spanning virtual (*n*=35,835; 38.3%), ambulatory (*n*=26,579; 28.4%), emergency department (*n*=23,388; 25.0%) and inpatient (*n*=7,726; 8.3%) facilities. We compared 180-day risk of PAS-related healthcare utilization among COVID-19 cases and influenza via adjusted hazard ratios (aHRs) weighted to account for cases' index infection type and follow-up retention. Risk of PAS diagnoses in any clinical setting was only modestly higher among COVID-19 cases in comparison to influenza cases within 31-90 days after cases' initial illness (aHR=1.04 [95%

				confidence interval: 0.99-1.09]; risk difference = 0.6 [–0.1-1.2] cases per 100 person-months). This difference was attenuated by 91-180 days (aHR=1.01 [0.97-1.06]; risk difference = 0.4 [–0.1-0.9] cases per 100 person-months). However, COVID-19 cases faced higher risk of severe PAS conditions requiring hospitalization (aHR=1.31 [1.07-1.59] and 1.24 [1.03-1.49] within 31-90 and 91-180 days, respectively). This excess risk of severe PAS was concentrated among COVID-19 cases hospitalized during acute-phase illness, and was attenuated among cases who received antiviral treatment, who had up-to-date vaccination status prior to infection, or who did not require inpatient admission for acute-phase illness.
**Introduction**				
Background/rationale	2	Explain the scientific background and rationale for the investigation being reported	Introduction, paragraph 2	…it remains unclear whether SARS-CoV-2 and influenza infections pose comparable risk for PAS, and whether the clinical phenotypes of PAS associated with SARS-CoV-2 and influenza are distinct.
Objectives	3	State specific objectives, including any prespecified hypotheses	Introduction, paragraph 3	We therefore assessed PAS-associated healthcare utilization among a large cohort of COVID-19 and influenza cases whose acute illnesses were managed across a spectrum of clinical settings, spanning

				virtual to inpatient care. We quantified differences in risk of PAS according to both infecting virus and severity of acute-phase illness.
**Methods**				
Study design	4	Present key elements of study design early in the paper	Methods, paragraph 2	We conducted an observational cohort study of all KPSC members aged ≥18 years with a positive molecular test for SARS-CoV-2 or influenza between September 1, 2022 and December 31, 2023.
Setting	5	Describe the setting, locations, and relevant dates, including periods of recruitment, exposure, follow-up, and data collection	Methods, paragraphs 1, 2, 5	Setting/locations: We analyzed data collected from members of Kaiser Permanente Southern California (KPSC), an integrated health-care organization comprised of 16 hospitals and 226 medical offices providing care to >4.8 million members across Southern California.<br><br>Relevant dates: We conducted an observational cohort study of all KPSC members aged ≥18 years with a positive molecular test for SARS-CoV-2 or influenza between September 1, 2022 and December 31, 2023.<br><br>Follow-up: We defined PAS as diagnoses occurring within distinct proximal (31-90 days) and distal (91-180 days) risk periods after cases' index date.
Participants	6	(*a*) *Cohort study*—Give the eligibility criteria, and the sources and methods of selection of participants. Describe methods of follow-up<br>*Case-control study*—Give the eligibility criteria, and the sources and methods of case ascertainment and control selection. Give the rationale for the choice of cases and controls	Methods, paragraphs 1-3	Eligibility: We restricted analyses to individuals with accompanying acute respiratory illness (ARI) diagnoses in any

	Cross-sectional study—Give the eligibility criteria, and the sources and methods of selection of participants	clinical setting between 7 days before or after the positive test, and defined index dates as the date of the first ARI diagnosis. This study is reported as per the Strengthening the Reporting of Observational Studies in Epidemiology (STROBE) guideline (**S1 Checklist**). While the study was retrospective in nature, the design and variable definitions followed a pre-specified protocol (**S2 File**).<br><br>Eligible participants had ≥1 year of continuous KPSC membership (allowing 45-day enrollment gap) before the positive test date to enable accurate capture of comorbid conditions and healthcare utilization. We limited analyses to the first documented ARI diagnosis associated with a positive SARS-CoV-2 or influenza test result in any 180-day period for each individual (index ARI episode). Multiple episodes were included from the same individual if these episodes occurred ≥180 days apart, and each studied episode could be associated with either SARS-CoV-2 or influenza. We excluded SARS-CoV-2 infections identified between 30 days before and 14 days after individuals received any COVID-19 vaccine dose, and influenza infections identified between 30 days before and 14

				days after individuals received any influenza vaccine, so that participants' recorded vaccination status at time of infection reflected doses from which they could be expected to have mounted a response. All individuals meeting eligibility criteria were included in analyses.<br><br>Follow-up: All aspects of clinical care received at KPSC facilities, including diagnoses, provider notes, laboratory tests, vaccinations, and prescriptions, are linked by a unique identifier in patients' electronic health records (EHRs). Insurance claims submitted for reimbursement capture care received from external healthcare providers, enabling near-complete capture of healthcare receipt among members.
		(*b*) *Cohort study*—For matched studies, give matching criteria and number of exposed and unexposed<br>*Case-control study*—For matched studies, give matching criteria and the number of controls per case		N/A (no matching)
Variables	7	Clearly define all outcomes, exposures, predictors, potential confounders, and effect modifiers. Give diagnostic criteria, if applicable	Methods, paragraphs 4-5, 7	**Exposures.** The primary exposure was the virus detected at each index ARI episode; we defined exposure groups as COVID-19 cases and influenza cases. We further stratified exposure groups based on respiratory viral season (October, 2022–September, 2023, and October–December, 2023) and by viral lineages

(influenza type A or B, where available from testing data, and putative SARS-CoV-2 variant based on dominant lineages circulating at the time of individuals' index date [40]). Additionally, we distinguished index episodes according to the highest-acuity care setting in which individuals received ARI diagnoses during the acute stage of their illness (within 7 days before to 30 days after the index date). In order from lowest to highest acuity, strata included virtual (online/telehealth) settings, ambulatory (ambulatory/urgent care) settings, emergency departments, and inpatient (hospital) facilities.

Outcomes. We defined PAS as diagnoses occurring within distinct proximal (31-90 days) and distal (91-180 days) risk periods after cases' index date. We used prespecified ICD-10 diagnoses codes categorized into 10 disease categories with similar mechanisms and body systems affected (cardiopulmonary, hemolytic, respiratory, musculoskeletal, renal, gastrointestinal, neurological, skin, endocrine, and mental health conditions; **S1 Table**). Outcomes encompassed both new-onset PAS and exacerbations of pre-existing conditions resulting in

post-acute healthcare utilization. Although there is no universal case definition for PAS, the study outcome encompassed all diagnosis codes included in US Centers for Disease Control (CDC) criteria for Post-COVID Conditions [2], augmented with definitions from other EHR-based studies [8,41–43]. This approach facilitates alignment with prior studies [2,16] and ongoing surveillance [44] employing CDC's EHR-based case definition. The list of codes used by CDC to define PAS is purposefully broad to avoid missing conditions potentially occurring as PAS, and prior studies have demonstrated the included codes occur over twice as often among COVID-19 cases in the year following infection as among matched controls not known to have experienced SARS-CoV-2 infection [2]. We conducted separate analyses for PAS within each disease category, and for a composite outcome of any PAS. Consistent with our classification for index ARI diagnoses, we took the highest-acuity care setting where PAS diagnoses were assigned in each follow-up period as an indicator of severity.

Confounders: All weighting models accounted for pre-specified covariates including

individuals' age group, sex, highest-acuity care setting for the index ARI episode, race/ethnicity, Charlson comorbidity index, history of diagnoses in each PAS disease category, history of depression, cigarette smoking, prior-year healthcare utilization (across ambulatory, emergency department, and inpatient settings), body mass index, COVID-19 and seasonal influenza vaccination status, receipt of antiviral treatment for the index ARI episode (as described below), calendar month of the index episode, and neighborhood deprivation index [50]. We included Charlson comorbidity index as a quantitative variable representing individuals' overall health status due to collinearity of individual Charlson conditions with individuals' history of diagnoses within the PAS disease categories. We categorized continuous variables (according to pre-specified breakpoints listed in **Table 1**) to avoid estimation of extreme weights in IPTW and IPCW models. We estimated risk differences for each outcome by subtracting weighted incidence rate estimates among influenza cases from weighted incidence rate estimates among COVID-19 cases.

Data sources/ measurement	8*	For each variable of interest, give sources of data and details of methods of assessment (measurement). Describe comparability of assessment methods if there is more than one group	Methods, paragraph 1	All aspects of clinical care received at KPSC facilities, including diagnoses, clinical notes, laboratory tests, and prescriptions, are linked by a unique identifier in patients' electronic health records (EHRs). Insurance claims submitted for reimbursement capture care received from external healthcare providers, enabling near-complete capture of healthcare receipt among members.
Bias	9	Describe any efforts to address potential sources of bias	Methods, paragraphs 7, 8	We used a two-stage weighting approach to mitigate selection bias (due to differential depletion of susceptibles prior to follow-up initiation) and confounding [45–47]. First, to address differential censoring prior to the beginning of each follow-up interval among COVID-19 cases and influenza cases, we generated inverse probability of censoring weights (IPCWs) addressing each individual's probability of remaining enrolled through the beginning of each 30-day period after index [48]. We estimated IPCWs via the Breslow estimator for survival curves from fitted Cox proportional hazards models with death or disenrollment 0-180 days after cases' index date as the outcome. We fit these models including data from all individuals meeting eligibility

criteria for analyses, including those censored before the post-acute follow-up period. Second, to account for differences in characteristics of COVID-19 cases and influenza cases, we also generated inverse probability of treatment (exposure) weights (IPTWs), addressing each individual's probability of an index infection with SARS-CoV-2 or influenza. We computed stabilized IPTWs via logistic regression models defining infecting virus as the outcome [49]… We estimated aHRs using Cox proportional hazards models, defining distinct observations for each 30-day period (31-60, 61-90, 91-120, 121-150, 151-180 days) after the index date to update IPCWs. Weights for each follow-up interval were the product of the individual's (time-varying) IPCW and the individual's (time-invariant) IPTW for infection with the identified virus [51]. We generated doubly-robust aHR estimates by including covariates used in the weighting models in analysis models, and used the sandwich variance estimator to account for repeated observations across multiple periods for each individual. We fit separate models for follow-up periods 31-90 days and 91-180 days after index. We verified the

				proportional hazards assumption within each follow-up period by testing for non-zero slopes of Schoenfeld residuals from fitted Cox proportional hazards models [52]. We repeated analyses defining alternative interval breaks for continuous covariates, and defining these covariates with continuous values, to verify findings were not sensitive to categorization schemes for continuous variables (**S2 Table**).
Study size	10	Explain how the study size was arrived at	Methods, paragraph 3	All individuals meeting eligibility criteria were included in analyses. As our study was retrospective in nature, there was no prespecified sample size or enrollment target.

Continued on next page

Quantitative variables	11	Explain how quantitative variables were handled in the analyses. If applicable, describe which groupings were chosen and why	Methods, paragraph 7	We categorized continuous variables (according to pre-specified breakpoints listed in **Table 1**) to avoid estimation of extreme weights in IPTW and IPCW models.
Statistical methods	12	(*a*) Describe all statistical methods, including those used to control for confounding	Methods, paragraphs 7, 8	We used a two-stage weighting approach to mitigate selection bias (due to differential depletion of susceptibles prior to follow-up initiation) and confounding [45–47]. First, to address differential censoring prior to the beginning of each follow-up interval among COVID-19 cases and influenza cases, we generated inverse probability of censoring weights (IPCWs) addressing each individual's probability of remaining enrolled through the beginning of each 30-day period after index [48]. We estimated IPCWs via the Breslow estimator for survival curves from fitted Cox proportional hazards models with death or disenrollment 0-180 days after cases' index date as the outcome. We fit these models including data from all individuals meeting eligibility criteria for analyses, including those censored before the post-acute follow-up period. Second, to account for differences in characteristics of COVID-19 cases and influenza cases, we also generated inverse probability of treatment (exposure) weights (IPTWs), addressing each individual's probability of an index infection with SARS-CoV-2 or influenza. We computed stabilized

			IPTWs via logistic regression models defining infecting virus as the outcome [49]… We estimated aHRs using Cox proportional hazards models, defining distinct observations for each 30-day period (31-60, 61-90, 91-120, 121-150, 151-180 days) after the index date to update IPCWs. Weights for each follow-up interval were the product of the individual's (time-varying) IPCW and the individual's (time-invariant) IPTW for infection with the identified virus [51]. We generated doubly-robust aHR estimates by including covariates used in the weighting models in analysis models, and used the sandwich variance estimator to account for repeated observations across multiple periods for each individual. We fit separate models for follow-up periods 31-90 days and 91-180 days after index. We verified the proportional hazards assumption within each follow-up period by testing for non-zero slopes of Schoenfeld residuals from fitted Cox proportional hazards models [52]. We repeated analyses defining alternative interval breaks for continuous covariates, and defining these covariates with continuous values, to verify findings were not sensitive to categorization schemes for continuous variables (**S2 Table**).
	(*b*) Describe any methods used to examine subgroups and interactions	Methods, paragraphs 9, 10	We conducted subgroup analyses restricted to individuals with or without history of prior diagnoses

corresponding to PAS conditions within each organ system category in the preceding year. Among persons with such history, we interpreted PAS outcomes as exacerbations of pre-existing conditions, such that aHR estimates conveyed the differences in risk of post-viral exacerbations among COVID-19 cases and influenza cases. Among persons without history of such diagnoses, we interpreted PAS outcomes as new-onset illness, such that aHR estimates conveyed the differential risk of new-onset PAS among COVID-19 cases and influenza cases.

Last, to explore whether other factors modified the relationship between infecting virus and PAS risk, we conducted subgroup analyses stratified by age, sex, severity of the index ARI episode, and receipt of antivirals and vaccines. We defined antiviral receipt for the index infection as dispenses of molnupiravir or nirmatrelvir-ritonavir (for COVID-19 cases) and oseltamivir, zanamivir, peramivir, or baloxavir (for influenza cases), occurring within 7 days before or after index. We defined up-to-date influenza vaccination status as receipt of seasonal influenza vaccination within 15-180 days before index. Based on US vaccination recommendations during the study period [53] and estimated durations

				of protection against SARS-CoV-2 infection after vaccination [54–56], we defined up-to-date COVID-19 vaccination as receipt of ≥3 COVID-19 vaccine doses, including one dose within 15-180 days before index.
		(*c*) Explain how missing data were addressed		N/A (no exclusions due to missing data; data were abstracted from electronic health records comprising all care utilization in the preceding year); for demographic characteristics, **Table 1** outlines the categorization and counts of "Unknown" values.
		(*d*) *Cohort study*—If applicable, explain how loss to follow-up was addressed<br>*Case-control study*—If applicable, explain how matching of cases and controls was addressed<br>*Cross-sectional study*—If applicable, describe analytical methods taking account of sampling strategy	Methods, paragraph 6	We censored observations at death, disenrollment, end of study period, or ARI associated with a distinct virus, whichever occurred first.
		(*e*) Describe any sensitivity analyses	Methods, paragraph 8	We repeated analyses defining alternative interval breaks for continuous covariates, and defining these covariates with continuous values, to verify findings were not sensitive to categorization schemes for continuous variables (**S2 Table**).
**Results**				
Participants	13*	(a) Report numbers of individuals at each stage of study—eg numbers potentially eligible, examined for eligibility, confirmed eligible, included in the study, completing follow-up, and analysed	Results, paragraph 1 and Flowchart (Figure 1)	Analyses included 74,738 eligible COVID-19 cases and 18,790 eligible influenza cases (**Figure 1**).
		(b) Give reasons for non-participation at each stage	N/A	N/A (retrospective study, no non-participation)
		(c) Consider use of a flow diagram	Figure 1	Figure 1
Descriptive data	14*	(a) Give characteristics of study participants (eg demographic, clinical, social) and information on exposures and potential confounders	Results, paragraph 1 and Table 1, Table S2, Table S3	In comparison to influenza cases, COVID-19 cases were older, had higher Charlson comorbidity index scores, and had higher ambulatory healthcare utilization in the year

				prior to index date as well as greater likelihood of hospital admission in the prior year (**Table 1**; **S3 Table**). Additionally, COVID-19 cases had higher likelihood of receiving care in either inpatient settings or in virtual settings only for their initial infection.
		(b) Indicate number of participants with missing data for each variable of interest	Table 1	N/A (no exclusions due to missing data; data were abstracted from electronic health records comprising all care utilization in the preceding year); for demographic characteristics, **Table 1** outlines the categorization and counts of "Unknown" values.
		(c) *Cohort study*—Summarise follow-up time (eg, average and total amount)	Results, paragraph 2	Overall, 97.3% and 95.3% of COVID-19 cases were retained in follow-up through 31 and 91 days after their index date, respectively (72,745 and 71,238 of 74,738, respectively), as were 97.9% and 95.7% of influenza cases (18,395 and 17,990 of 18,790, respectively; **Table S2**).
Outcome data	15*	*Cohort study*—Report numbers of outcome events or summary measures over time	Results, paragraph 3	Within 31-90 days after index, weighted incidence rates of PAS diagnoses per 100 person-months at risk were 26.5 (95% confidence interval: 26.2-26.8) among COVID-19 cases and 25.9 (25.4-26.5) among influenza cases. Within 91-180 days after index, weighted incidence rates were 23.0 (22.7-23.2) and 22.6 (22.1-23.0) per 100 person-months among COVID-19 and influenza cases, respectively.
		Case-control study—Report numbers in each exposure category, or summary measures of exposure		
		Cross-sectional study—Report numbers of outcome events or summary measures		

Main results	16	(*a*) Give unadjusted estimates and, if applicable, confounder-adjusted estimates and their precision (eg, 95% confidence interval). Make clear which confounders were adjusted for and why they were included	Results, paragraphs 3-5	Risk differences totaled 0.6 (–0.1-1.2) additional COVID-19 cases experiencing PAS per 100 person-months within 31-90 days after index, and 0.4 (–0.1-0.9) additional COVID-19 cases experiencing PAS per 100 person-months within 91-180 days after index… Risk differences corresponded to 0.2 (0.1-0.4) and 0.1 (0.0-0.2) additional COVID-19 cases experiencing PAS requiring inpatient admission per 100 person-months within 31-90 and 91-180 days after index, respectively. These differences increased with severity of cases' initial ARI (**Table 3**). Among cases whose initial ARI necessitated inpatient care, risk differences corresponded to 3.2 (2.2-4.1) and 1.5 (0.8-2.2) additional COVID-19 cases receiving PAS diagnoses in inpatient settings per 100 person-months within 31-90 and 91-180 days after index, respectively.<br><br>Based on estimates from doubly-robust Cox proportional hazards models, COVID-19 cases experienced modestly higher risk of PAS in comparison to influenza cases within 31-90 days after index (aHR=1.04 [95% confidence interval: 0.99-1.09]), which were attenuated by 91-180 days after index (aHR=1.01 [0.97-1.06]; **Table 3**). However, COVID-19 cases had 31% (7-59%) and 24% (3-49%) higher risk than influenza cases of receiving PAS diagnoses in

			inpatient settings 31-90 days and 91-180 days after index in comparison to influenza cases, respectively. Findings were consistent in sensitivity analyses with alternative handling of continuous covariates in weighting and adjustment models (**S5 Table**).<br><br>Point estimates favored higher risk of inpatient PAS diagnoses among COVID-19 cases versus influenza cases in the 31-90 day follow-up period across all categories of PAS diagnoses, spanning 1.23 (0.93-1.61) for renal PAS to 7.27 (1.55-34.09) for skin-associated PAS (**Table 4**). Although likewise elevated across all disease categories in the 91-180 day follow-up period, point estimates were lower in this period relative to the 31-90 day period for all categories except musculoskeletal PAS (aHR=2.04 [1.29-3.24]) and neurological PAS (aHR=2.11 [1.54-2.90]). For each syndromic category, the association of COVID-19 with increased risk of PAS was weaker for diagnoses within virtual, ambulatory, and emergency department settings than for PAS diagnoses within inpatient settings.
	(*b*) Report category boundaries when continuous variables were categorized		
	(*c*) If relevant, consider translating estimates of relative risk into absolute risk for a meaningful time period	Results, paragraph 3; Table 2, Table 3	Risk differences totaled 0.6 (–0.1-1.2) additional COVID-19 cases experiencing PAS per 100 person-months within 31-90 days after index, and 0.4 (–0.1-0.9) additional COVID-19 cases experiencing PAS

	per 100 person-months within 91-180 days after index… Risk differences corresponded to 0.2 (0.1-0.4) and 0.1 (0.0-0.2) additional COVID-19 cases experiencing PAS requiring inpatient admission per 100 person-months within 31-90 and 91-180 days after index, respectively. These differences increased with severity of cases' initial ARI (**Table 3**). Among cases whose initial ARI necessitated inpatient care, risk differences corresponded to 3.2 (2.2-4.1) and 1.5 (0.8-2.2) additional COVID-19 cases receiving PAS diagnoses in inpatient settings per 100 person-months within 31-90 and 91-180 days after index, respectively.

Continued on next page

Other analyses	17	Report other analyses done—eg analyses of subgroups and interactions, and sensitivity analyses	Results, paragraphs 6-9	Differences between COVID-19 cases and influenza cases in risk of post-acute exacerbations of pre-existing conditions were also mainly evident for PAS diagnoses in inpatient settings (**Table 5**). Within 31-90 days after index, point estimates spanned 19-122% higher risk for inpatient PAS exacerbations of pre-existing conditions across all syndromic categories. Within 91-180 days after index, point estimates were attenuated across all categories except musculoskeletal and neurological conditions (aHR=3.16 [1.76-5.69] and 2.61 [1.84-3.71], respectively). Similarly, point estimates favored greater risk of new-onset PAS conditions necessitating inpatient care among COVID-19 cases, with aHR point estimates spanning 20-373% greater risk within 31-90 days after cases' index dates across all disease categories. Within 91-180 days after index, renal PAS (aHR=2.31 [1.26-4.24]) and skin-associated PAS (aHR=3.25 [0.71-14.85]) showed the greatest elevation among COVID-19 cases in comparison to influenza cases for diagnoses in inpatient settings. Generally, aHR estimates were associated with greater statistical uncertainty for comparisons of new-onset PAS diagnoses than for PAS exacerbations, reflecting the low incidence of new-onset PAS diagnoses compared to PAS

exacerbations among both COVID-19 cases and influenza cases (**S6 Table**).

Higher risk of inpatient PAS diagnoses among COVID-19 cases compared to influenza cases was primarily apparent among patients whose index ARI episodes necessitated inpatient admission (**Table 6**). Compared to influenza cases who received ARI diagnoses in inpatient settings, COVID-19 cases who received ARI diagnoses in inpatient settings had 73% (30-130%) and 49% (9-106%) higher risk of inpatient PAS diagnoses 31-90 and 91-180 days after index, respectively. Evidence of increased risk of PAS diagnoses among COVID-19 cases was inconsistent among patients whose index ARI episodes were managed in lower-acuity care settings. Differences in risk of inpatient PAS diagnoses among COVID-19 cases and influenza cases were apparent only among cases who did not receive antiviral treatment (aHR=2.09 [1.45-3.03] for untreated COVID-19 cases versus untreated influenza cases within 31-90 days after index). We did not identify differences in risk of inpatient PAS diagnoses among COVID-19 cases and influenza cases who were up-to-date with COVID-19 and influenza vaccines, respectively, at the time of their initial illness. Differences in risk of inpatient-diagnosed PAS

				conditions among COVID-19 cases versus influenza cases were more strongly apparent among female cases than male cases; additionally, differences in PAS risk among COVID-19 cases versus influenza cases appeared within multiple age groups.<br><br>Our finding that COVID-19 cases experienced greater risk than influenza cases of severe PAS necessitating hospital admission held within analyses disaggregated by season (2022-23 and 2023-24), SARS-CoV-2 variant period (BA.4/BA.5, XBB/XBB.1.5, and BA.2.86/JN.1), and influenza comparator virus (A or B), although these analyses encountered lower statistical power than primary analyses (**S7 Table**). Consistent with our primary analyses, we did not identify appreciable differences in risk of PAS outcomes that included diagnoses in lower-acuity settings within most subgroups (**Table 6**; **S7 Table**).
**Discussion**				
Key results	18	Summarise key results with reference to study objectives	Discussion, paragraph 1	Within our study, COVID-19 cases experienced only modestly greater risk of PAS diagnoses up to 180 days after their index ARI episode in comparison to influenza cases. The primary distinguishing characteristic of PAS following COVID-19 versus influenza was that COVID-19 cases experienced higher risk of severe PAS necessitating inpatient admission.

				Further, these differences in risk were dependent on the severity of cases' index infection. Whereas risk of severe PAS was comparable among COVID-19 cases and influenza cases who received care for their index episode in virtual, ambulatory, or emergency department settings, COVID-19 cases hospitalized for their index infection had higher risk of severe PAS than influenza cases who were hospitalized for their index infection. Differences in PAS risk were attenuated in strata of patients who received antiviral treatment or whose vaccination status was up-to-date against their infecting virus. Our findings demonstrate that although COVID-19 and influenza cases both experience substantial risk of PAS, the spectrum of PAS occurring after COVID-19 includes more severe manifestations than PAS occurring after influenza. This heightened risk of severe PAS is concentrated among patients who experienced severe COVID-19, and may be attenuated by vaccination or antiviral treatment.
Limitations	19	Discuss limitations of the study, taking into account sources of potential bias or imprecision. Discuss both direction and magnitude of any potential bias	Discussion, paragraph 5	Limitations of our analyses should also be considered. First, studied PAS outcomes could be more strongly associated with SARS-CoV-2 infection than with influenza infection simply because our PAS code list was assembled in part from findings of prior studies documenting that the

studied outcomes occurred at elevated rates among COVID-19 survivors [2,8,41–43]. However, our consideration of a purposefully broad case definition, and our findings of only weak statistical evidence for associations of the studied outcome with COVID-19, together lessen this concern. Gold-standard case definitions for PAS remain to be established, and case definitions based on healthcare utilization recorded in EHR data differ inherently from those based on patient-reported symptoms, physical examination, and laboratory criteria [62,63]. Second, our analysis was limited to adults. Children also experience PAS associated with SARS-CoV-2 [64] and other respiratory viruses [65,66], suggesting similar studies should be undertaken in pediatric cohorts. Third, testing practices for SARS-CoV-2 and influenza differed during the study period. Whereas SARS-CoV-2 testing was widely undertaken for both inpatients and in ambulatory settings, influenza testing was less widely available and may have been reserved for individuals with more severe illness, or those receiving an initial negative SARS-CoV-2 test result. Our analyses adjusted for measured differences between COVID-19 cases and influenza cases; however, bias due to unmeasured variables and other factors may persist under our analytic

framework [67]. Fourth, as our study was restricted to individuals who received ARI diagnoses, our findings did not address PAS potentially associated with asymptomatic SARS-CoV-2 and influenza infections. Fifth, our analyses did not address differences in PAS risk beyond 180 days after cases' index dates, limiting our ability to compare differences in the lengths of time COVID-19 cases and influenza cases may be at risk for PAS. Regardless, attenuated effect estimates by 91-180 days after index suggests longer-term differences are unlikely. Considering only first PAS events over each follow-up interval diminishes our ability to compare the nature of PAS experienced among COVID-19 and influenza cases, including the frequency or persistence of clinical complaints. Sixth, although KPSC members represent the demographic and socioeconomic diversity of the surrounding population, healthcare delivery for KPSC members may not reflect patterns among other providers, impacting external generalizability of our findings. Last, while our study included adjustment and subgroup analyses for individuals receiving common outpatient antiviral therapies, the effects of earlier COVID-19 treatment strategies (including monoclonal antibodies or

				remdesivir) on PAS risk remain poorly understood.
Interpretation	20	Give a cautious overall interpretation of results considering objectives, limitations, multiplicity of analyses, results from similar studies, and other relevant evidence	Discussion, paragraph 6	We found that COVID-19 cases experienced only modestly higher risk of PAS in comparison to influenza cases, although PAS among COVID-19 cases were more likely to require hospital admission than PAS among influenza cases. Whereas PAS are widely known to occur after COVID-19, our results suggest that the risk of PAS associated with influenza may be under-appreciated and worthy of further study. For COVID-19, rigorous estimates of the burden of PAS are necessary for the contemporary context of novel circulating variants and population immunity; such analyses should adjust for counterfactual risk absent infection through control-group comparisons [68]. Aside from influenza, PAS are also known to occur in association with numerous other respiratory viruses [69], including other sarbecoviruses [70], RSV [71], enteroviruses [72], and Epstein-Barr virus [73]. Improved understanding of this post-acute burden can collectively inform the value of interventions aiming to prevent or mitigate the severity of respiratory virus infections.
Generalisability	21	Discuss the generalisability (external validity) of the study results	Discussion, paragraph 5	Although KPSC members represent the demographic and socioeconomic diversity of the surrounding population, healthcare delivery for KPSC members may

				not reflect patterns among other providers, impacting external generalizability of our findings.
**Other information**				
Funding	22	Give the source of funding and the role of the funders for the present study and, if applicable, for the original study on which the present article is based	Acknowledgments, paragraph 3	**Funding:** This work was supported by the US Centers for Disease Control and Prevention (grant 75D30123-C-18129 to SYT). Two co-authors are employed by the funder of the study (LRF and SS are employees of the US Centers for Disease Control and Prevention) and contributed to the design of the study in addition to providing critical intellectual feedback and revisions on drafts of the manuscript. The funder was not involved in the collection or analysis of data. Clearance of the manuscript by the funder of the study was required prior to submission for publication.

*Give information separately for cases and controls in case-control studies and, if applicable, for exposed and unexposed groups in cohort and cross-sectional studies.

Note: An Explanation and Elaboration article discusses each checklist item and gives methodological background and published examples of transparent reporting. The STROBE checklist is best used in conjunction with this article (freely available on the Web sites of PLoS Medicine at http://www.plosmedicine.org/, Annals of Internal Medicine at http://www.annals.org/, and Epidemiology at http://www.epidem.com/). Information on the STROBE Initiative is available at www.strobe-statement.org.
