# Supplementary material for: Comparative risk of post-acute sequelae following SARS-CoV-2 or influenza virus infection: A retrospective cohort study among United States adults
Source: PLoS Med. 2025 Oct 9;22(10):e1004777. doi: 10.1371/journal.pmed.1004777 (PMC12551960; doi:10.1371/journal.pmed.1004777)
Supplement: S1 Table — (PDF) [file pmed.1004777.s004.pdf]

Table S1: Diagnosis codes (ICD-10-CM) used to define post-acute sequelae.

ICD10 code	Description	Sub-condition
**Cardiovascular system disorders**		
I200	Unstable angina	Cardiovascular disease
I201	Angina pectoris with documented spasm	Cardiovascular disease
I208	Other forms of angina pectoris	Cardiovascular disease
I209	Angina pectoris, unspecified	Cardiovascular disease
I240	Acute coronary thrombosis not resulting in myocardial infarction	Cardiovascular disease
I248	Other forms of acute ischemic heart disease	Cardiovascular disease
I249	Acute ischemic heart disease, unspecified	Cardiovascular disease
I2510	Atherosclerotic heart disease of native coronary artery without angina pectoris	Cardiovascular disease
I25110	Atherosclerotic heart disease of native coronary artery with unstable angina pectoris	Cardiovascular disease
I25111	Atherosclerotic heart disease of native coronary artery with angina pectoris with	Cardiovascular disease
I25118	Atherosclerotic heart disease of native coronary artery with other forms of	Cardiovascular disease
I25119	Atherosclerotic heart disease of native coronary artery with unspecified angina pectoris	Cardiovascular disease
I256	Silent myocardial ischemia	Cardiovascular disease
I25700	Atherosclerosis of coronary artery bypass graft(s), unspecified, with unstable angina pectoris	Cardiovascular disease
I25701	Atherosclerosis of coronary artery bypass graft(s), unspecified, with angina pectoris with	Cardiovascular disease
I25708	Atherosclerosis of coronary artery bypass graft(s), unspecified, with other forms of	Cardiovascular disease
I25709	Atherosclerosis of coronary artery bypass graft(s), unspecified, with unspecified angina pectoris	Cardiovascular disease
I25710	Atherosclerosis of autologous vein coronary artery bypass graft(s) with unstable angina	Cardiovascular disease
I25711	Atherosclerosis of autologous vein coronary artery bypass graft(s) with angina pectoris	Cardiovascular disease
I25718	Atherosclerosis of autologous vein coronary artery bypass graft(s) with other forms	Cardiovascular disease
I25719	Atherosclerosis of autologous vein coronary artery bypass graft(s) with unspecified angina	Cardiovascular disease
I25720	Atherosclerosis of autologous artery coronary artery bypass graft(s) with unstable angina	Cardiovascular disease
I25721	Atherosclerosis of autologous artery coronary artery bypass graft(s) with angina pectoris	Cardiovascular disease
I25728	Atherosclerosis of autologous artery coronary artery bypass graft(s) with other forms	Cardiovascular disease
I25729	Atherosclerosis of autologous artery coronary artery bypass graft(s) with unspecified angina	Cardiovascular disease
I25730	Atherosclerosis of nonautologous biological coronary artery bypass graft(s) with unstable angina	Cardiovascular disease
I25731	Atherosclerosis of nonautologous biological coronary artery bypass graft(s) with angina pectoris	Cardiovascular disease
I25738	Atherosclerosis of nonautologous biological coronary artery bypass graft(s) with other forms	Cardiovascular disease
I25739	Atherosclerosis of nonautologous biological coronary artery bypass graft(s) with unspecified angina	Cardiovascular disease
I25750	Atherosclerosis of native coronary artery of transplanted heart with unstable angina	Cardiovascular disease
I25751	Atherosclerosis of native coronary artery of transplanted heart with angina pectoris	Cardiovascular disease
I25758	Atherosclerosis of native coronary artery of transplanted heart with other forms	Cardiovascular disease
I25759	Atherosclerosis of native coronary artery of transplanted heart with unspecified angina	Cardiovascular disease
I25760	Atherosclerosis of bypass graft of coronary artery of transplanted heart with	Cardiovascular disease
I25761	Atherosclerosis of bypass graft of coronary artery of transplanted heart with	Cardiovascular disease
I25768	Atherosclerosis of bypass graft of coronary artery of transplanted heart with	Cardiovascular disease
I25769	Atherosclerosis of bypass graft of coronary artery of transplanted heart with	Cardiovascular disease
I25790	Atherosclerosis of other coronary artery bypass graft(s) with unstable angina pectoris	Cardiovascular disease
I25791	Atherosclerosis of other coronary artery bypass graft(s) with angina pectoris with	Cardiovascular disease
I25798	Atherosclerosis of other coronary artery bypass graft(s) with other forms of	Cardiovascular disease
I25799	Atherosclerosis of other coronary artery bypass graft(s) with unspecified angina pectoris	Cardiovascular disease
I25810	Atherosclerosis of coronary artery bypass graft(s) without angina pectoris	Cardiovascular disease
I25811	Atherosclerosis of native coronary artery of transplanted heart without angina pectoris	Cardiovascular disease
I25812	Atherosclerosis of bypass graft of coronary artery of transplanted heart without	Cardiovascular disease
I2582	Chronic total occlusion of coronary artery	Cardiovascular disease
I2583	Coronary atherosclerosis due to lipid rich plaque	Cardiovascular disease
I2584	Coronary atherosclerosis due to calcified coronary lesion	Cardiovascular disease
I2589	Other forms of chronic ischemic heart disease	Cardiovascular disease
I259	Chronic ischemic heart disease, unspecified	Cardiovascular disease
Z9861	Coronary angioplasty status	Cardiovascular disease
I470	Re-entry ventricular arrhythmia	Cardiac dysrhythmia
I471	Supraventricular tachycardia	Cardiac dysrhythmia
I472	Ventricular tachycardia	Cardiac dysrhythmia
I4720	Ventricular tachycardia unspecified	Cardiac dysrhythmia
I4721	torsades de pointed	Cardiac dysrhythmia
I4729	Other ventricular tachycardia	Cardiac dysrhythmia
I479	Paroxysmal tachycardia, unspecified	Cardiac dysrhythmia
I480	Paroxysmal atrial fibrillation	Cardiac dysrhythmia
I481	Persistent atrial fibrillation	Cardiac dysrhythmia
I4811	Longstanding persistent atrial fibrillation	Cardiac dysrhythmia
I4819	Other persistent atrial fibrillation	Cardiac dysrhythmia
I4820	Unspecified chronic atrial fibrillation	Cardiac dysrhythmia
I4821	Permanent atrial fibrillation	Cardiac dysrhythmia
I483	Typical atrial flutter	Cardiac dysrhythmia
I484	Atypical atrial flutter	Cardiac dysrhythmia

I4891	Unspecified atrial fibrillation	Cardiac dysrhythmia
I4892	Unspecified atrial flutter	Cardiac dysrhythmia
I4901	Ventricular fibrillation	Cardiac dysrhythmia
I4902	Ventricular flutter	Cardiac dysrhythmia
I491	Atrial premature depolarization	Cardiac dysrhythmia
I492	Junctional premature depolarization	Cardiac dysrhythmia
I493	Ventricular premature depolarization	Cardiac dysrhythmia
I4940	Unspecified premature depolarization	Cardiac dysrhythmia
I4949	Other premature depolarization	Cardiac dysrhythmia
I495	Sick sinus syndrome	Cardiac dysrhythmia
I498	Other specified cardiac arrhythmias	Cardiac dysrhythmia
I499	Cardiac arrhythmia, unspecified	Cardiac dysrhythmia
R000	Tachycardia, unspecified	Cardiac dysrhythmia
R001	Bradycardia, unspecified	Cardiac dysrhythmia
R002	Palpitations	Cardiac dysrhythmia
R008	Other abnormalities of heart beat	Cardiac dysrhythmia
R009	Unspecified abnormalities of heart beat	Cardiac dysrhythmia
R010	Benign and innocent cardiac murmurs	Cardiac dysrhythmia
R011	Cardiac murmur, unspecified	Cardiac dysrhythmia
R012	Other cardiac sounds	Cardiac dysrhythmia
R030	Elevated blood-pressure reading, without diagnosis of hypertension	Cardiac dysrhythmia
R0989	Other specified symptoms and signs involving the circulatory and respiratory systems	CHF
I050	Rheumatic mitral stenosis	CHF
I051	Rheumatic mitral insufficiency	CHF
I052	Rheumatic mitral stenosis with insufficiency	CHF
I058	Other rheumatic mitral valve diseases	CHF
I059	Rheumatic mitral valve disease, unspecified	CHF
I060	Rheumatic aortic stenosis	CHF
I061	Rheumatic aortic insufficiency	CHF
I062	Rheumatic aortic stenosis with insufficiency	CHF
I068	Other rheumatic aortic valve diseases	CHF
I069	Rheumatic aortic valve disease, unspecified	CHF
I070	Rheumatic tricuspid stenosis	CHF
I071	Rheumatic tricuspid insufficiency	CHF
I072	Rheumatic tricuspid stenosis and insufficiency	CHF
I078	Other rheumatic tricuspid valve diseases	CHF
I079	Rheumatic tricuspid valve disease, unspecified	CHF
I080	Rheumatic disorders of both mitral and aortic valves	CHF
I081	Rheumatic disorders of both mitral and tricuspid valves	CHF
I082	Rheumatic disorders of both aortic and tricuspid valves	CHF
I083	Combined rheumatic disorders of mitral, aortic and tricuspid valves	CHF
I088	Other rheumatic multiple valve diseases	CHF
I089	Rheumatic multiple valve disease, unspecified	CHF
I090	Rheumatic myocarditis	CHF
I091	Rheumatic diseases of endocardium, valve unspecified	CHF
I092	Chronic rheumatic pericarditis	CHF
I0981	Rheumatic heart failure	CHF
I0989	Other specified rheumatic heart diseases	CHF
I502	Other CHF	CHF
I503	Other CHF	CHF
I099	Rheumatic heart disease, unspecified	CHF
I2101	ST elevation (STEMI) myocardial infarction involving left main coronary artery	Acute MI
I2102	ST elevation (STEMI) myocardial infarction involving left anterior descending coronary artery	Acute MI
I2109	ST elevation (STEMI) myocardial infarction involving other coronary artery of anterior	Acute MI
I2111	ST elevation (STEMI) myocardial infarction involving right coronary artery	Acute MI
I2119	ST elevation (STEMI) myocardial infarction involving other coronary artery of inferior	Acute MI
I2121	ST elevation (STEMI) myocardial infarction involving left circumflex coronary artery	Acute MI
I2129	ST elevation (STEMI) myocardial infarction involving other sites	Acute MI
I213	ST elevation (STEMI) myocardial infarction of unspecified site	Acute MI
I214	Non-ST elevation (NSTEMI) myocardial infarction	Acute MI
I219	Acute myocardial infarction, unspecified	Acute MI
I21A1	Myocardial infarction type 2	Acute MI
I21A9	Other myocardial infarction type	Acute MI
I220	Subsequent ST elevation (STEMI) myocardial infarction of anterior wall	Acute MI
I221	Subsequent ST elevation (STEMI) myocardial infarction of inferior wall	Acute MI
I222	Subsequent non-ST elevation (NSTEMI) myocardial infarction	Acute MI
I228	Subsequent ST elevation (STEMI) myocardial infarction of other sites	Acute MI
I229	Subsequent ST elevation (STEMI) myocardial infarction of unspecified site	Acute MI
A3681	Diphtheritic cardiomyopathy	Myocarditis and cardiomyopathy

B3320	Viral carditis, unspecified	Myocarditis and cardiomyopathy
B3322	Viral myocarditis	Myocarditis and cardiomyopathy
B3324	Viral cardiomyopathy	Myocarditis and cardiomyopathy
B5881	Toxoplasma myocarditis	Myocarditis and cardiomyopathy
I255	Ischemic cardiomyopathy	Myocarditis and cardiomyopathy
I400	Infective myocarditis	Myocarditis and cardiomyopathy
I401	Isolated myocarditis	Myocarditis and cardiomyopathy
I408	Other acute myocarditis	Myocarditis and cardiomyopathy
I409	Acute myocarditis, unspecified	Myocarditis and cardiomyopathy
I41	Myocarditis in diseases classified elsewhere	Myocarditis and cardiomyopathy
I420	Dilated cardiomyopathy	Myocarditis and cardiomyopathy
I421	Obstructive hypertrophic cardiomyopathy	Myocarditis and cardiomyopathy
I422	Other hypertrophic cardiomyopathy	Myocarditis and cardiomyopathy
I423	Endomyocardial (eosinophilic) disease	Myocarditis and cardiomyopathy
I424	Endocardial fibroelastosis	Myocarditis and cardiomyopathy
I425	Other restrictive cardiomyopathy	Myocarditis and cardiomyopathy
I428	Other cardiomyopathies	Myocarditis and cardiomyopathy
I429	Cardiomyopathy, unspecified	Myocarditis and cardiomyopathy
I43	Cardiomyopathy in diseases classified elsewhere	Myocarditis and cardiomyopathy
I514	Myocarditis, unspecified	Myocarditis and cardiomyopathy
J1082	Influenza due to other identified influenza virus with myocarditis	Myocarditis and cardiomyopathy
J1182	Influenza due to unidentified influenza virus with myocarditis	Myocarditis and cardiomyopathy
O903	Peripartum cardiomyopathy	Myocarditis and cardiomyopathy
B3321	Viral endocarditis	Myocarditis and cardiomyopathy
I27	Pulmonary hypertension (all subcodes)	Pulmonary hypertension
I10	Essential (primary) hypertension	Hypertension
I11	Hypertensive heart disease	Hypertension
I12	Hypertensive chronic kidney disease	Hypertension
I13	Hypertensive heart and chronic kidney disease	Hypertension
I15	Secondary hypertension	Hypertension
I16	Hypertensive crisis	Hypertension
R570	Cardiogenic shock	Cardiogenic shock
**Hemolytic and vascular system disorders**		
D473	Essential (hemorrhagic) thrombocythemia	Coagulation and hemorrhagic
D65	Disseminated intravascular coagulation [defibrination syndrome]	Coagulation and hemorrhagic
D68311	Acquired hemophilia	Coagulation and hemorrhagic
D68312	Antiphospholipid antibody with hemorrhagic disorder	Coagulation and hemorrhagic
D68318	Other hemorrhagic disorder due to intrinsic circulating anticoagulants, antibodies, or inhibitors	Coagulation and hemorrhagic
D6832	Hemorrhagic disorder due to extrinsic circulating anticoagulants	Coagulation and hemorrhagic
D684	Acquired coagulation factor deficiency	Coagulation and hemorrhagic
D6851	Activated protein C resistance	Coagulation and hemorrhagic
D6852	Prothrombin gene mutation	Coagulation and hemorrhagic
D6859	Other primary thrombophilia	Coagulation and hemorrhagic
D6861	Antiphospholipid syndrome	Coagulation and hemorrhagic
D6862	Lupus anticoagulant syndrome	Coagulation and hemorrhagic
D6869	Other thrombophilia	Coagulation and hemorrhagic
D688	Other specified coagulation defects	Coagulation and hemorrhagic
D689	Coagulation defect, unspecified	Coagulation and hemorrhagic
D690	Allergic purpura	Coagulation and hemorrhagic
D691	Qualitative platelet defects	Coagulation and hemorrhagic
D692	Other nonthrombocytopenic purpura	Coagulation and hemorrhagic
D693	Immune thrombocytopenic purpura	Coagulation and hemorrhagic
D6941	Evans syndrome	Coagulation and hemorrhagic
D6942	Congenital and hereditary thrombocytopenia purpura	Coagulation and hemorrhagic
D6949	Other primary thrombocytopenia	Coagulation and hemorrhagic
D6951	Posttransfusion purpura	Coagulation and hemorrhagic
D6959	Other secondary thrombocytopenia	Coagulation and hemorrhagic
D696	Thrombocytopenia, unspecified	Coagulation and hemorrhagic
D698	Other specified hemorrhagic conditions	Coagulation and hemorrhagic
D699	Hemorrhagic condition, unspecified	Coagulation and hemorrhagic
D7582	Heparin induced thrombocytopenia (HIT)	Coagulation and hemorrhagic
D75838	Other thrombocytosis	Coagulation and hemorrhagic
D75839	Thrombocytosis, unspecified	Coagulation and hemorrhagic
M362	Hemophilic arthropathy	Coagulation and hemorrhagic
D473	Essential (hemorrhagic) thrombocythemia	Coagulation and hemorrhagic
D65	Disseminated intravascular coagulation [defibrination syndrome]	Coagulation and hemorrhagic
D68311	Acquired hemophilia	Coagulation and hemorrhagic
D68312	Antiphospholipid antibody with hemorrhagic disorder	Coagulation and hemorrhagic
D68318	Other hemorrhagic disorder due to intrinsic circulating anticoagulants, antibodies, or inhibitors	Coagulation and hemorrhagic

D6832	Hemorrhagic disorder due to extrinsic circulating anticoagulants	Coagulation and hemorrhagic
D684	Acquired coagulation factor deficiency	Coagulation and hemorrhagic
D6851	Activated protein C resistance	Coagulation and hemorrhagic
D6852	Prothrombin gene mutation	Coagulation and hemorrhagic
D6859	Other primary thrombophilia	Coagulation and hemorrhagic
D6861	Antiphospholipid syndrome	Coagulation and hemorrhagic
D6862	Lupus anticoagulant syndrome	Coagulation and hemorrhagic
D6869	Other thrombophilia	Coagulation and hemorrhagic
D688	Other specified coagulation defects	Coagulation and hemorrhagic
D689	Coagulation defect, unspecified	Coagulation and hemorrhagic
D690	Allergic purpura	Coagulation and hemorrhagic
D691	Qualitative platelet defects	Coagulation and hemorrhagic
D692	Other nonthrombocytopenic purpura	Coagulation and hemorrhagic
D693	Immune thrombocytopenic purpura	Coagulation and hemorrhagic
D6941	Evans syndrome	Coagulation and hemorrhagic
D6942	Congenital and hereditary thrombocytopenia purpura	Coagulation and hemorrhagic
D6949	Other primary thrombocytopenia	Coagulation and hemorrhagic
D6951	Posttransfusion purpura	Coagulation and hemorrhagic
D6959	Other secondary thrombocytopenia	Coagulation and hemorrhagic
D696	Thrombocytopenia, unspecified	Coagulation and hemorrhagic
D698	Other specified hemorrhagic conditions	Coagulation and hemorrhagic
D699	Hemorrhagic condition, unspecified	Coagulation and hemorrhagic
D7582	Heparin induced thrombocytopenia (HIT)	Coagulation and hemorrhagic
D75838	Other thrombocytosis	Coagulation and hemorrhagic
D75839	Thrombocytosis, unspecified	Coagulation and hemorrhagic
M362	Hemophilic arthropathy	Coagulation and hemorrhagic
D686	Other thrombophilia	Coagulation and hemorrhagic
D72819	Decreased white blood cell count	Coagulation and hemorrhagic
I82401	Acute embolism and thrombosis of unspecified deep veins of right lower	Thromboembolic event
I82402	Acute embolism and thrombosis of unspecified deep veins of left lower	Thromboembolic event
I82403	Acute embolism and thrombosis of unspecified deep veins of lower extremity,	Thromboembolic event
I82409	Acute embolism and thrombosis of unspecified deep veins of unspecified lower	Thromboembolic event
I82491	Acute embolism and thrombosis of other specified deep vein of right	Thromboembolic event
I82492	Acute embolism and thrombosis of other specified deep vein of left	Thromboembolic event
I82493	Acute embolism and thrombosis of other specified deep vein of lower	Thromboembolic event
I82499	Acute embolism and thrombosis of other specified deep vein of unspecified	Thromboembolic event
I824Y1	Acute embolism and thrombosis of unspecified deep veins of right proximal	Thromboembolic event
I824Y2	Acute embolism and thrombosis of unspecified deep veins of left proximal	Thromboembolic event
I824Y3	Acute embolism and thrombosis of unspecified deep veins of proximal lower	Thromboembolic event
I824Y9	Acute embolism and thrombosis of unspecified deep veins of unspecified proximal	Thromboembolic event
I824Z1	Acute embolism and thrombosis of unspecified deep veins of right distal	Thromboembolic event
I824Z2	Acute embolism and thrombosis of unspecified deep veins of left distal	Thromboembolic event
I824Z3	Acute embolism and thrombosis of unspecified deep veins of distal lower	Thromboembolic event
I824Z9	Acute embolism and thrombosis of unspecified deep veins of unspecified distal	Thromboembolic event
I82621	Acute embolism and thrombosis of deep veins of right upper extremity	Thromboembolic event
I82622	Acute embolism and thrombosis of deep veins of left upper extremity	Thromboembolic event
I82623	Acute embolism and thrombosis of deep veins of upper extremity, bilateral	Thromboembolic event
I82629	Acute embolism and thrombosis of deep veins of unspecified upper extremity	Thromboembolic event
I82501	Chronic embolism and thrombosis of unspecified deep veins of right lower	Thromboembolic event
I82502	Chronic embolism and thrombosis of unspecified deep veins of left lower	Thromboembolic event
I82503	Chronic embolism and thrombosis of unspecified deep veins of lower extremity,	Thromboembolic event
I82509	Chronic embolism and thrombosis of unspecified deep veins of unspecified lower	Thromboembolic event
I82591	Chronic embolism and thrombosis of other specified deep vein of right	Thromboembolic event
I82592	Chronic embolism and thrombosis of other specified deep vein of left	Thromboembolic event
I82593	Chronic embolism and thrombosis of other specified deep vein of lower	Thromboembolic event
I82599	Chronic embolism and thrombosis of other specified deep vein of unspecified	Thromboembolic event
I825Y1	Chronic embolism and thrombosis of unspecified deep veins of right proximal	Thromboembolic event
I825Y2	Chronic embolism and thrombosis of unspecified deep veins of left proximal	Thromboembolic event
I825Y3	Chronic embolism and thrombosis of unspecified deep veins of proximal lower	Thromboembolic event
I825Y9	Chronic embolism and thrombosis of unspecified deep veins of unspecified proximal	Thromboembolic event
I825Z1	Chronic embolism and thrombosis of unspecified deep veins of right distal	Thromboembolic event
I825Z2	Chronic embolism and thrombosis of unspecified deep veins of left distal	Thromboembolic event
I825Z3	Chronic embolism and thrombosis of unspecified deep veins of distal lower	Thromboembolic event
I825Z9	Chronic embolism and thrombosis of unspecified deep veins of unspecified distal	Thromboembolic event
I82721	Chronic embolism and thrombosis of deep veins of right upper extremity	Thromboembolic event
I82722	Chronic embolism and thrombosis of deep veins of left upper extremity	Thromboembolic event
I82723	Chronic embolism and thrombosis of deep veins of upper extremity, bilateral	Thromboembolic event
I82729	Chronic embolism and thrombosis of deep veins of unspecified upper extremity	Thromboembolic event
I82	Other venous embolism and thrombosis (all subcodes)	Thromboembolic event

J40	Middle cerebral artery syndrome	Cerebrovascular disease
G461	Anterior cerebral artery syndrome	Cerebrovascular disease
G462	Posterior cerebral artery syndrome	Cerebrovascular disease
G463	Brain stem stroke syndrome	Cerebrovascular disease
G464	Cerebellar stroke syndrome	Cerebrovascular disease
G465	Pure motor lacunar syndrome	Cerebrovascular disease
G466	Pure sensory lacunar syndrome	Cerebrovascular disease
G467	Other lacunar syndromes	Cerebrovascular disease
G468	Other vascular syndromes of brain in cerebrovascular diseases	Cerebrovascular disease
I671	Cerebral aneurysm, nonruptured	Cerebrovascular disease
I672	Cerebral atherosclerosis	Cerebrovascular disease
I673	Progressive vascular leukoencephalopathy	Cerebrovascular disease
I675	Moyamoya disease	Cerebrovascular disease
I676	Nonpyogenic thrombosis of intracranial venous system	Cerebrovascular disease
I677	Cerebral arteritis, not elsewhere classified	Cerebrovascular disease
I6781	Acute cerebrovascular insufficiency	Cerebrovascular disease
I6782	Cerebral ischemia	Cerebrovascular disease
I6783	Posterior reversible encephalopathy syndrome	Cerebrovascular disease
I67841	Reversible cerebrovascular vasoconstriction syndrome	Cerebrovascular disease
I67848	Other cerebrovascular vasospasm and vasoconstriction	Cerebrovascular disease
I67850	Cerebral autosomal dominant arteriopathy with subcortical infarcts and leukoencephalopathy	Cerebrovascular disease
I67858	Other hereditary cerebrovascular disease	Cerebrovascular disease
I6789	Other cerebrovascular disease	Cerebrovascular disease
I679	Cerebrovascular disease, unspecified	Cerebrovascular disease
I680	Cerebral amyloid angiopathy	Cerebrovascular disease
I682	Cerebral arteritis in other diseases classified elsewhere	Cerebrovascular disease
I688	Other cerebrovascular disorders in diseases classified elsewhere	Cerebrovascular disease
I60	Nontraumatic subarachnoid hemorrhage	Cerebrovascular disease
I61	Nontraumatic intracerebral hemorrhage	Cerebrovascular disease
I62	Other and unspecified nontraumatic intracranial hemorrhage	Cerebrovascular disease
I63	Cerebral infarction	Cerebrovascular disease
I69	Sequelae of cerebrovascular disease	Cerebrovascular disease
I679	Cerebrovascular disease, unspecified	Cerebrovascular disease
I680	Cerebral amyloid angiopathy	Cerebrovascular disease
I682	Cerebral arteritis in other diseases classified elsewhere	Cerebrovascular disease
I688	Other cerebrovascular disorders in diseases classified elsewhere	Cerebrovascular disease
I60	Nontraumatic subarachnoid hemorrhage	Cerebrovascular disease
I61	Nontraumatic intracerebral hemorrhage	Cerebrovascular disease
I62	Other and unspecified nontraumatic intracranial hemorrhage	Cerebrovascular disease
I63	Cerebral infarction	Cerebrovascular disease
I69	Sequelae of cerebrovascular disease	Cerebrovascular disease
**Respiratory System disorders**		
J4520	Mild intermittent asthma, uncomplicated	Asthma
J4521	Mild intermittent asthma with (acute) exacerbation	Asthma
J4522	Mild intermittent asthma with status asthmaticus	Asthma
J4530	Mild persistent asthma, uncomplicated	Asthma
J4531	Mild persistent asthma with (acute) exacerbation	Asthma
J4532	Mild persistent asthma with status asthmaticus	Asthma
J4540	Moderate persistent asthma, uncomplicated	Asthma
J4541	Moderate persistent asthma with (acute) exacerbation	Asthma
J4542	Moderate persistent asthma with status asthmaticus	Asthma
J4550	Severe persistent asthma, uncomplicated	Asthma
J4551	Severe persistent asthma with (acute) exacerbation	Asthma
J4552	Severe persistent asthma with status asthmaticus	Asthma
J45901	Unspecified asthma with (acute) exacerbation	Asthma
J45902	Unspecified asthma with status asthmaticus	Asthma
J45909	Unspecified asthma, uncomplicated	Asthma
J45990	Exercise induced bronchospasm	Asthma
J45991	Cough variant asthma	Asthma
J45998	Other asthma	Asthma
R040	Epistaxis	Respiratory symptoms
R041	Hemorrhage from throat	Respiratory symptoms
R042	Hemoptysis	Respiratory symptoms
R0481	Acute idiopathic pulmonary hemorrhage in infants	Respiratory symptoms
R0489	Hemorrhage from other sites in respiratory passages	Respiratory symptoms
R049	Hemorrhage from respiratory passages, unspecified	Respiratory symptoms
R05	Cough	Respiratory symptoms
R051	Acute cough	Respiratory symptoms
R052	Subacute cough	Respiratory symptoms

R053	Chronic cough	Respiratory symptoms
R054	Cough syncope	Respiratory symptoms
R058	Other specified cough	Respiratory symptoms
R059	Cough, unspecified	Respiratory symptoms
R0600	Dyspnea, unspecified	Respiratory symptoms
R0601	Orthopnea	Respiratory symptoms
R0602	Shortness of breath	Respiratory symptoms
R0603	Acute respiratory distress	Respiratory symptoms
R0609	Other forms of dyspnea	Respiratory symptoms
R061	Stridor	Respiratory symptoms
R062	Wheezing	Respiratory symptoms
R064	Hyperventilation	Respiratory symptoms
R066	Hiccough	Respiratory symptoms
R067	Sneezing	Respiratory symptoms
R0681	Apnea, not elsewhere classified	Respiratory symptoms
R0682	Tachypnea, not elsewhere classified	Respiratory symptoms
R0689	Other abnormalities of breathing	Respiratory symptoms
R069	Unspecified abnormalities of breathing	Respiratory symptoms
R070	Pain in throat	Respiratory symptoms
R071	Chest pain on breathing	Respiratory symptoms
R0781	Pleurodynia	Respiratory symptoms
R0782	Intercostal pain	Respiratory symptoms
R0901	Asphyxia	Respiratory symptoms
R0902	Hypoxemia	Respiratory symptoms
R093	Abnormal sputum	Respiratory symptoms
R0981	Nasal congestion	Respiratory symptoms
R0982	Postnasal drip	Respiratory symptoms
R072	pleurodynia	Respiratory symptoms
R078	intercostal	Respiratory symptoms
R079	other chest pain	Respiratory symptoms
I2601	Septic pulmonary embolism with acute cor pulmonale	Acute pulmonary embolism
I2602	Saddle embolus of pulmonary artery with acute cor pulmonale	Acute pulmonary embolism
I2609	Other pulmonary embolism with acute cor pulmonale	Acute pulmonary embolism
I2690	Septic pulmonary embolism without acute cor pulmonale	Acute pulmonary embolism
I2692	Saddle embolus of pulmonary artery without acute cor pulmonale	Acute pulmonary embolism
I2693	Single subsegmental pulmonary embolism without acute cor pulmonale	Acute pulmonary embolism
I2694	Multiple subsegmental pulmonary emboli without acute cor pulmonale	Acute pulmonary embolism
I2699	Other pulmonary embolism without acute cor pulmonale	Acute pulmonary embolism
J9610	Chronic respiratory failure unspecified whether with hypoxia or hypercapnia	COPD and Bronchitis
J9611	Chronic respiratory failure WITH HYPOXIA	COPD and Bronchitis
J9612	Chronic respiratory failure WITH HYPERCAPNIA	COPD and Bronchitis
J20	Acute bronchitis	COPD and Bronchitis
J40	Bronchitis, not specified as acute or chronic	COPD and Bronchitis
J41	Simple and mucopurulent chronic bronchitis	COPD and Bronchitis
J42	Unspecified chronic bronchitis	COPD and Bronchitis
J84	Interstitial lung disease	Interstitial lung disease
J960	Acute respiratory failure	Acute respiratory failure
J81	Pulmonary edema	Pulmonary edema
**Musculoskeletal system disorders**		
R531	Weakness	Malaise and fatigue
R5381	Other malaise	Malaise and fatigue
R5382	Chronic fatigue, unspecified	Malaise and fatigue
R5383	Other fatigue	Malaise and fatigue
M2550	Pain in unspecified joint	Musculoskeletal pain
M25511	Pain in right shoulder	Musculoskeletal pain
M25512	Pain in left shoulder	Musculoskeletal pain
M25519	Pain in unspecified shoulder	Musculoskeletal pain
M25521	Pain in right elbow	Musculoskeletal pain
M25522	Pain in left elbow	Musculoskeletal pain
M25529	Pain in unspecified elbow	Musculoskeletal pain
M25531	Pain in right wrist	Musculoskeletal pain
M25532	Pain in left wrist	Musculoskeletal pain
M25539	Pain in unspecified wrist	Musculoskeletal pain
M25541	Pain in joints of right hand	Musculoskeletal pain
M25542	Pain in joints of left hand	Musculoskeletal pain
M25549	Pain in joints of unspecified hand	Musculoskeletal pain
M25551	Pain in right hip	Musculoskeletal pain
M25552	Pain in left hip	Musculoskeletal pain
M25559	Pain in unspecified hip	Musculoskeletal pain

M25561	Pain in right knee	Musculoskeletal pain
M25562	Pain in left knee	Musculoskeletal pain
M25569	Pain in unspecified knee	Musculoskeletal pain
M25571	Pain in right ankle and joints of right foot	Musculoskeletal pain
M25572	Pain in left ankle and joints of left foot	Musculoskeletal pain
M25579	Pain in unspecified ankle and joints of unspecified foot	Musculoskeletal pain
M2559	Pain in other specified joint	Musculoskeletal pain
M2560	Stiffness of unspecified joint, not elsewhere classified	Musculoskeletal pain
M25611	Stiffness of right shoulder, not elsewhere classified	Musculoskeletal pain
M25612	Stiffness of left shoulder, not elsewhere classified	Musculoskeletal pain
M25619	Stiffness of unspecified shoulder, not elsewhere classified	Musculoskeletal pain
M25621	Stiffness of right elbow, not elsewhere classified	Musculoskeletal pain
M25622	Stiffness of left elbow, not elsewhere classified	Musculoskeletal pain
M25629	Stiffness of unspecified elbow, not elsewhere classified	Musculoskeletal pain
M25631	Stiffness of right wrist, not elsewhere classified	Musculoskeletal pain
M25632	Stiffness of left wrist, not elsewhere classified	Musculoskeletal pain
M25639	Stiffness of unspecified wrist, not elsewhere classified	Musculoskeletal pain
M25641	Stiffness of right hand, not elsewhere classified	Musculoskeletal pain
M25642	Stiffness of left hand, not elsewhere classified	Musculoskeletal pain
M25649	Stiffness of unspecified hand, not elsewhere classified	Musculoskeletal pain
M25651	Stiffness of right hip, not elsewhere classified	Musculoskeletal pain
M25652	Stiffness of left hip, not elsewhere classified	Musculoskeletal pain
M25659	Stiffness of unspecified hip, not elsewhere classified	Musculoskeletal pain
M25661	Stiffness of right knee, not elsewhere classified	Musculoskeletal pain
M25662	Stiffness of left knee, not elsewhere classified	Musculoskeletal pain
M25669	Stiffness of unspecified knee, not elsewhere classified	Musculoskeletal pain
M25671	Stiffness of right ankle, not elsewhere classified	Musculoskeletal pain
M25672	Stiffness of left ankle, not elsewhere classified	Musculoskeletal pain
M25673	Stiffness of unspecified ankle, not elsewhere classified	Musculoskeletal pain
M25674	Stiffness of right foot, not elsewhere classified	Musculoskeletal pain
M25675	Stiffness of left foot, not elsewhere classified	Musculoskeletal pain
M25676	Stiffness of unspecified foot, not elsewhere classified	Musculoskeletal pain
M2569	Stiffness of other specified joint, not elsewhere classified	Musculoskeletal pain
M546	Pain in thoracic spine	Musculoskeletal pain
M5481	Occipital neuralgia	Musculoskeletal pain
M5489	Other dorsalgia	Musculoskeletal pain
M549	Dorsalgia, unspecified	Musculoskeletal pain
M791	Myalgia	Musculoskeletal pain
M7910	Myalgia, unspecified site	Musculoskeletal pain
M7911	Myalgia of mastication muscle	Musculoskeletal pain
M7912	Myalgia of auxiliary muscles, head and neck	Musculoskeletal pain
M7918	Myalgia, other site	Musculoskeletal pain
M79601	Pain in right arm	Musculoskeletal pain
M79602	Pain in left arm	Musculoskeletal pain
M79603	Pain in arm, unspecified	Musculoskeletal pain
M79604	Pain in right leg	Musculoskeletal pain
M79605	Pain in left leg	Musculoskeletal pain
M79606	Pain in leg, unspecified	Musculoskeletal pain
M79609	Pain in unspecified limb	Musculoskeletal pain
M79621	Pain in right upper arm	Musculoskeletal pain
M79622	Pain in left upper arm	Musculoskeletal pain
M79629	Pain in unspecified upper arm	Musculoskeletal pain
M79631	Pain in right forearm	Musculoskeletal pain
M79632	Pain in left forearm	Musculoskeletal pain
M79639	Pain in unspecified forearm	Musculoskeletal pain
M79641	Pain in right hand	Musculoskeletal pain
M79642	Pain in left hand	Musculoskeletal pain
M79643	Pain in unspecified hand	Musculoskeletal pain
M79644	Pain in right finger(s)	Musculoskeletal pain
M79645	Pain in left finger(s)	Musculoskeletal pain
M79646	Pain in unspecified finger(s)	Musculoskeletal pain
M79651	Pain in right thigh	Musculoskeletal pain
M79652	Pain in left thigh	Musculoskeletal pain
M79659	Pain in unspecified thigh	Musculoskeletal pain
M79661	Pain in right lower leg	Musculoskeletal pain
M79662	Pain in left lower leg	Musculoskeletal pain
M79669	Pain in unspecified lower leg	Musculoskeletal pain
M79671	Pain in right foot	Musculoskeletal pain
M79672	Pain in left foot	Musculoskeletal pain

M79673	Pain in unspecified foot	Musculoskeletal pain
M79674	Pain in right toe(s)	Musculoskeletal pain
M79675	Pain in left toe(s)	Musculoskeletal pain
M79676	Pain in unspecified toe(s)	Musculoskeletal pain
M60000	Infective myositis, unspecified right arm	Muscle disorders
M60001	Infective myositis, unspecified left arm	Muscle disorders
M60002	Infective myositis, unspecified arm	Muscle disorders
M60003	Infective myositis, unspecified right leg	Muscle disorders
M60004	Infective myositis, unspecified left leg	Muscle disorders
M60005	Infective myositis, unspecified leg	Muscle disorders
M60009	Infective myositis, unspecified site	Muscle disorders
M60011	Infective myositis, right shoulder	Muscle disorders
M60012	Infective myositis, left shoulder	Muscle disorders
M60019	Infective myositis, unspecified shoulder	Muscle disorders
M60021	Infective myositis, right upper arm	Muscle disorders
M60022	Infective myositis, left upper arm	Muscle disorders
M60029	Infective myositis, unspecified upper arm	Muscle disorders
M60031	Infective myositis, right forearm	Muscle disorders
M60032	Infective myositis, left forearm	Muscle disorders
M60039	Infective myositis, unspecified forearm	Muscle disorders
M60041	Infective myositis, right hand	Muscle disorders
M60042	Infective myositis, left hand	Muscle disorders
M60043	Infective myositis, unspecified hand	Muscle disorders
M60044	Infective myositis, right finger(s)	Muscle disorders
M60045	Infective myositis, left finger(s)	Muscle disorders
M60046	Infective myositis, unspecified finger(s)	Muscle disorders
M60051	Infective myositis, right thigh	Muscle disorders
M60052	Infective myositis, left thigh	Muscle disorders
M60059	Infective myositis, unspecified thigh	Muscle disorders
M60061	Infective myositis, right lower leg	Muscle disorders
M60062	Infective myositis, left lower leg	Muscle disorders
M60069	Infective myositis, unspecified lower leg	Muscle disorders
M60070	Infective myositis, right ankle	Muscle disorders
M60071	Infective myositis, left ankle	Muscle disorders
M60072	Infective myositis, unspecified ankle	Muscle disorders
M60073	Infective myositis, right foot	Muscle disorders
M60074	Infective myositis, left foot	Muscle disorders
M60075	Infective myositis, unspecified foot	Muscle disorders
M60076	Infective myositis, right toe(s)	Muscle disorders
M60077	Infective myositis, left toe(s)	Muscle disorders
M60078	Infective myositis, unspecified toe(s)	Muscle disorders
M6008	Infective myositis, other site	Muscle disorders
M6009	Infective myositis, multiple sites	Muscle disorders
M6010	Interstitial myositis of unspecified site	Muscle disorders
M60111	Interstitial myositis, right shoulder	Muscle disorders
M60112	Interstitial myositis, left shoulder	Muscle disorders
M60119	Interstitial myositis, unspecified shoulder	Muscle disorders
M60121	Interstitial myositis, right upper arm	Muscle disorders
M60122	Interstitial myositis, left upper arm	Muscle disorders
M60129	Interstitial myositis, unspecified upper arm	Muscle disorders
M60131	Interstitial myositis, right forearm	Muscle disorders
M60132	Interstitial myositis, left forearm	Muscle disorders
M60139	Interstitial myositis, unspecified forearm	Muscle disorders
M60141	Interstitial myositis, right hand	Muscle disorders
M60142	Interstitial myositis, left hand	Muscle disorders
M60149	Interstitial myositis, unspecified hand	Muscle disorders
M60151	Interstitial myositis, right thigh	Muscle disorders
M60152	Interstitial myositis, left thigh	Muscle disorders
M60159	Interstitial myositis, unspecified thigh	Muscle disorders
M60161	Interstitial myositis, right lower leg	Muscle disorders
M60162	Interstitial myositis, left lower leg	Muscle disorders
M60169	Interstitial myositis, unspecified lower leg	Muscle disorders
M60171	Interstitial myositis, right ankle and foot	Muscle disorders
M60172	Interstitial myositis, left ankle and foot	Muscle disorders
M60179	Interstitial myositis, unspecified ankle and foot	Muscle disorders
M6018	Interstitial myositis, other site	Muscle disorders
M6019	Interstitial myositis, multiple sites	Muscle disorders
M6080	Other myositis, unspecified site	Muscle disorders
M60811	Other myositis, right shoulder	Muscle disorders

M60812	Other myositis, left shoulder	Muscle disorders
M60819	Other myositis, unspecified shoulder	Muscle disorders
M60821	Other myositis, right upper arm	Muscle disorders
M60822	Other myositis, left upper arm	Muscle disorders
M60829	Other myositis, unspecified upper arm	Muscle disorders
M60831	Other myositis, right forearm	Muscle disorders
M60832	Other myositis, left forearm	Muscle disorders
M60839	Other myositis, unspecified forearm	Muscle disorders
M60841	Other myositis, right hand	Muscle disorders
M60842	Other myositis, left hand	Muscle disorders
M60849	Other myositis, unspecified hand	Muscle disorders
M60851	Other myositis, right thigh	Muscle disorders
M60852	Other myositis, left thigh	Muscle disorders
M60859	Other myositis, unspecified thigh	Muscle disorders
M60861	Other myositis, right lower leg	Muscle disorders
M60862	Other myositis, left lower leg	Muscle disorders
M60869	Other myositis, unspecified lower leg	Muscle disorders
M60871	Other myositis, right ankle and foot	Muscle disorders
M60872	Other myositis, left ankle and foot	Muscle disorders
M60879	Other myositis, unspecified ankle and foot	Muscle disorders
M6088	Other myositis, other site	Muscle disorders
M6089	Other myositis, multiple sites	Muscle disorders
M609	Myositis, unspecified	Muscle disorders
M6100	Myositis ossificans traumatica, unspecified site	Muscle disorders
M61011	Myositis ossificans traumatica, right shoulder	Muscle disorders
M61012	Myositis ossificans traumatica, left shoulder	Muscle disorders
M61019	Myositis ossificans traumatica, unspecified shoulder	Muscle disorders
M61021	Myositis ossificans traumatica, right upper arm	Muscle disorders
M61022	Myositis ossificans traumatica, left upper arm	Muscle disorders
M61029	Myositis ossificans traumatica, unspecified upper arm	Muscle disorders
M61031	Myositis ossificans traumatica, right forearm	Muscle disorders
M61032	Myositis ossificans traumatica, left forearm	Muscle disorders
M61039	Myositis ossificans traumatica, unspecified forearm	Muscle disorders
M61041	Myositis ossificans traumatica, right hand	Muscle disorders
M61042	Myositis ossificans traumatica, left hand	Muscle disorders
M61049	Myositis ossificans traumatica, unspecified hand	Muscle disorders
M61051	Myositis ossificans traumatica, right thigh	Muscle disorders
M61052	Myositis ossificans traumatica, left thigh	Muscle disorders
M61059	Myositis ossificans traumatica, unspecified thigh	Muscle disorders
M61061	Myositis ossificans traumatica, right lower leg	Muscle disorders
M61062	Myositis ossificans traumatica, left lower leg	Muscle disorders
M61069	Myositis ossificans traumatica, unspecified lower leg	Muscle disorders
M61071	Myositis ossificans traumatica, right ankle and foot	Muscle disorders
M61072	Myositis ossificans traumatica, left ankle and foot	Muscle disorders
M61079	Myositis ossificans traumatica, unspecified ankle and foot	Muscle disorders
M6108	Myositis ossificans traumatica, other site	Muscle disorders
M6109	Myositis ossificans traumatica, multiple sites	Muscle disorders
M6110	Myositis ossificans progressiva, unspecified site	Muscle disorders
M61111	Myositis ossificans progressiva, right shoulder	Muscle disorders
M61112	Myositis ossificans progressiva, left shoulder	Muscle disorders
M61119	Myositis ossificans progressiva, unspecified shoulder	Muscle disorders
M61121	Myositis ossificans progressiva, right upper arm	Muscle disorders
M61122	Myositis ossificans progressiva, left upper arm	Muscle disorders
M61129	Myositis ossificans progressiva, unspecified arm	Muscle disorders
M61131	Myositis ossificans progressiva, right forearm	Muscle disorders
M61132	Myositis ossificans progressiva, left forearm	Muscle disorders
M61139	Myositis ossificans progressiva, unspecified forearm	Muscle disorders
M61141	Myositis ossificans progressiva, right hand	Muscle disorders
M61142	Myositis ossificans progressiva, left hand	Muscle disorders
M61143	Myositis ossificans progressiva, unspecified hand	Muscle disorders
M61144	Myositis ossificans progressiva, right finger(s)	Muscle disorders
M61145	Myositis ossificans progressiva, left finger(s)	Muscle disorders
M61146	Myositis ossificans progressiva, unspecified finger(s)	Muscle disorders
M61151	Myositis ossificans progressiva, right thigh	Muscle disorders
M61152	Myositis ossificans progressiva, left thigh	Muscle disorders
M61159	Myositis ossificans progressiva, unspecified thigh	Muscle disorders
M61161	Myositis ossificans progressiva, right lower leg	Muscle disorders
M61162	Myositis ossificans progressiva, left lower leg	Muscle disorders
M61169	Myositis ossificans progressiva, unspecified lower leg	Muscle disorders

M61171	Myositis ossificans progressiva, right ankle	Muscle disorders
M61172	Myositis ossificans progressiva, left ankle	Muscle disorders
M61173	Myositis ossificans progressiva, unspecified ankle	Muscle disorders
M61174	Myositis ossificans progressiva, right foot	Muscle disorders
M61175	Myositis ossificans progressiva, left foot	Muscle disorders
M61176	Myositis ossificans progressiva, unspecified foot	Muscle disorders
M61177	Myositis ossificans progressiva, right toe(s)	Muscle disorders
M61178	Myositis ossificans progressiva, left toe(s)	Muscle disorders
M61179	Myositis ossificans progressiva, unspecified toe(s)	Muscle disorders
M6118	Myositis ossificans progressiva, other site	Muscle disorders
M6119	Myositis ossificans progressiva, multiple sites	Muscle disorders
M6120	Paralytic calcification and ossification of muscle, unspecified site	Muscle disorders
M61211	Paralytic calcification and ossification of muscle, right shoulder	Muscle disorders
M61212	Paralytic calcification and ossification of muscle, left shoulder	Muscle disorders
M61219	Paralytic calcification and ossification of muscle, unspecified shoulder	Muscle disorders
M61221	Paralytic calcification and ossification of muscle, right upper arm	Muscle disorders
M61222	Paralytic calcification and ossification of muscle, left upper arm	Muscle disorders
M61229	Paralytic calcification and ossification of muscle, unspecified upper arm	Muscle disorders
M61231	Paralytic calcification and ossification of muscle, right forearm	Muscle disorders
M61232	Paralytic calcification and ossification of muscle, left forearm	Muscle disorders
M61239	Paralytic calcification and ossification of muscle, unspecified forearm	Muscle disorders
M61241	Paralytic calcification and ossification of muscle, right hand	Muscle disorders
M61242	Paralytic calcification and ossification of muscle, left hand	Muscle disorders
M61249	Paralytic calcification and ossification of muscle, unspecified hand	Muscle disorders
M61251	Paralytic calcification and ossification of muscle, right thigh	Muscle disorders
M61252	Paralytic calcification and ossification of muscle, left thigh	Muscle disorders
M61259	Paralytic calcification and ossification of muscle, unspecified thigh	Muscle disorders
M61261	Paralytic calcification and ossification of muscle, right lower leg	Muscle disorders
M61262	Paralytic calcification and ossification of muscle, left lower leg	Muscle disorders
M61269	Paralytic calcification and ossification of muscle, unspecified lower leg	Muscle disorders
M61271	Paralytic calcification and ossification of muscle, right ankle and foot	Muscle disorders
M61272	Paralytic calcification and ossification of muscle, left ankle and foot	Muscle disorders
M61279	Paralytic calcification and ossification of muscle, unspecified ankle and foot	Muscle disorders
M6128	Paralytic calcification and ossification of muscle, other site	Muscle disorders
M6129	Paralytic calcification and ossification of muscle, multiple sites	Muscle disorders
M6130	Calcification and ossification of muscles associated with burns, unspecified site	Muscle disorders
M61311	Calcification and ossification of muscles associated with burns, right shoulder	Muscle disorders
M61312	Calcification and ossification of muscles associated with burns, left shoulder	Muscle disorders
M61319	Calcification and ossification of muscles associated with burns, unspecified shoulder	Muscle disorders
M61321	Calcification and ossification of muscles associated with burns, right upper arm	Muscle disorders
M61322	Calcification and ossification of muscles associated with burns, left upper arm	Muscle disorders
M61329	Calcification and ossification of muscles associated with burns, unspecified upper arm	Muscle disorders
M61331	Calcification and ossification of muscles associated with burns, right forearm	Muscle disorders
M61332	Calcification and ossification of muscles associated with burns, left forearm	Muscle disorders
M61339	Calcification and ossification of muscles associated with burns, unspecified forearm	Muscle disorders
M61341	Calcification and ossification of muscles associated with burns, right hand	Muscle disorders
M61342	Calcification and ossification of muscles associated with burns, left hand	Muscle disorders
M61349	Calcification and ossification of muscles associated with burns, unspecified hand	Muscle disorders
M61351	Calcification and ossification of muscles associated with burns, right thigh	Muscle disorders
M61352	Calcification and ossification of muscles associated with burns, left thigh	Muscle disorders
M61359	Calcification and ossification of muscles associated with burns, unspecified thigh	Muscle disorders
M61361	Calcification and ossification of muscles associated with burns, right lower leg	Muscle disorders
M61362	Calcification and ossification of muscles associated with burns, left lower leg	Muscle disorders
M61369	Calcification and ossification of muscles associated with burns, unspecified lower leg	Muscle disorders
M61371	Calcification and ossification of muscles associated with burns, right ankle and	Muscle disorders
M61372	Calcification and ossification of muscles associated with burns, left ankle and	Muscle disorders
M61379	Calcification and ossification of muscles associated with burns, unspecified ankle and	Muscle disorders
M6138	Calcification and ossification of muscles associated with burns, other site	Muscle disorders
M6139	Calcification and ossification of muscles associated with burns, multiple sites	Muscle disorders
M6140	Other calcification of muscle, unspecified site	Muscle disorders
M61411	Other calcification of muscle, right shoulder	Muscle disorders
M61412	Other calcification of muscle, left shoulder	Muscle disorders
M61419	Other calcification of muscle, unspecified shoulder	Muscle disorders
M61421	Other calcification of muscle, right upper arm	Muscle disorders
M61422	Other calcification of muscle, left upper arm	Muscle disorders
M61429	Other calcification of muscle, unspecified upper arm	Muscle disorders
M61431	Other calcification of muscle, right forearm	Muscle disorders
M61432	Other calcification of muscle, left forearm	Muscle disorders
M61439	Other calcification of muscle, unspecified forearm	Muscle disorders

M61441	Other calcification of muscle, right hand	Muscle disorders
M61442	Other calcification of muscle, left hand	Muscle disorders
M61449	Other calcification of muscle, unspecified hand	Muscle disorders
M61451	Other calcification of muscle, right thigh	Muscle disorders
M61452	Other calcification of muscle, left thigh	Muscle disorders
M61459	Other calcification of muscle, unspecified thigh	Muscle disorders
M61461	Other calcification of muscle, right lower leg	Muscle disorders
M61462	Other calcification of muscle, left lower leg	Muscle disorders
M61469	Other calcification of muscle, unspecified lower leg	Muscle disorders
M61471	Other calcification of muscle, right ankle and foot	Muscle disorders
M61472	Other calcification of muscle, left ankle and foot	Muscle disorders
M61479	Other calcification of muscle, unspecified ankle and foot	Muscle disorders
M6148	Other calcification of muscle, other site	Muscle disorders
M6149	Other calcification of muscle, multiple sites	Muscle disorders
M6150	Other ossification of muscle, unspecified site	Muscle disorders
M61511	Other ossification of muscle, right shoulder	Muscle disorders
M61512	Other ossification of muscle, left shoulder	Muscle disorders
M61519	Other ossification of muscle, unspecified shoulder	Muscle disorders
M61521	Other ossification of muscle, right upper arm	Muscle disorders
M61522	Other ossification of muscle, left upper arm	Muscle disorders
M61529	Other ossification of muscle, unspecified upper arm	Muscle disorders
M61531	Other ossification of muscle, right forearm	Muscle disorders
M61532	Other ossification of muscle, left forearm	Muscle disorders
M61539	Other ossification of muscle, unspecified forearm	Muscle disorders
M61541	Other ossification of muscle, right hand	Muscle disorders
M61542	Other ossification of muscle, left hand	Muscle disorders
M61549	Other ossification of muscle, unspecified hand	Muscle disorders
M61551	Other ossification of muscle, right thigh	Muscle disorders
M61552	Other ossification of muscle, left thigh	Muscle disorders
M61559	Other ossification of muscle, unspecified thigh	Muscle disorders
M61561	Other ossification of muscle, right lower leg	Muscle disorders
M61562	Other ossification of muscle, left lower leg	Muscle disorders
M61569	Other ossification of muscle, unspecified lower leg	Muscle disorders
M61571	Other ossification of muscle, right ankle and foot	Muscle disorders
M61572	Other ossification of muscle, left ankle and foot	Muscle disorders
M61579	Other ossification of muscle, unspecified ankle and foot	Muscle disorders
M6158	Other ossification of muscle, other site	Muscle disorders
M6159	Other ossification of muscle, multiple sites	Muscle disorders
M619	Calcification and ossification of muscle, unspecified	Muscle disorders
M6200	Separation of muscle (nontraumatic), unspecified site	Muscle disorders
M62011	Separation of muscle (nontraumatic), right shoulder	Muscle disorders
M62012	Separation of muscle (nontraumatic), left shoulder	Muscle disorders
M62019	Separation of muscle (nontraumatic), unspecified shoulder	Muscle disorders
M62021	Separation of muscle (nontraumatic), right upper arm	Muscle disorders
M62022	Separation of muscle (nontraumatic), left upper arm	Muscle disorders
M62029	Separation of muscle (nontraumatic), unspecified upper arm	Muscle disorders
M62031	Separation of muscle (nontraumatic), right forearm	Muscle disorders
M62032	Separation of muscle (nontraumatic), left forearm	Muscle disorders
M62039	Separation of muscle (nontraumatic), unspecified forearm	Muscle disorders
M62041	Separation of muscle (nontraumatic), right hand	Muscle disorders
M62042	Separation of muscle (nontraumatic), left hand	Muscle disorders
M62049	Separation of muscle (nontraumatic), unspecified hand	Muscle disorders
M62051	Separation of muscle (nontraumatic), right thigh	Muscle disorders
M62052	Separation of muscle (nontraumatic), left thigh	Muscle disorders
M62059	Separation of muscle (nontraumatic), unspecified thigh	Muscle disorders
M62061	Separation of muscle (nontraumatic), right lower leg	Muscle disorders
M62062	Separation of muscle (nontraumatic), left lower leg	Muscle disorders
M62069	Separation of muscle (nontraumatic), unspecified lower leg	Muscle disorders
M62071	Separation of muscle (nontraumatic), right ankle and foot	Muscle disorders
M62072	Separation of muscle (nontraumatic), left ankle and foot	Muscle disorders
M62079	Separation of muscle (nontraumatic), unspecified ankle and foot	Muscle disorders
M6208	Separation of muscle (nontraumatic), other site	Muscle disorders
M6210	Other rupture of muscle (nontraumatic), unspecified site	Muscle disorders
M62111	Other rupture of muscle (nontraumatic), right shoulder	Muscle disorders
M62112	Other rupture of muscle (nontraumatic), left shoulder	Muscle disorders
M62119	Other rupture of muscle (nontraumatic), unspecified shoulder	Muscle disorders
M62121	Other rupture of muscle (nontraumatic), right upper arm	Muscle disorders
M62122	Other rupture of muscle (nontraumatic), left upper arm	Muscle disorders
M62129	Other rupture of muscle (nontraumatic), unspecified upper arm	Muscle disorders

M62131	Other rupture of muscle (nontraumatic), right forearm	Muscle disorders
M62132	Other rupture of muscle (nontraumatic), left forearm	Muscle disorders
M62139	Other rupture of muscle (nontraumatic), unspecified forearm	Muscle disorders
M62141	Other rupture of muscle (nontraumatic), right hand	Muscle disorders
M62142	Other rupture of muscle (nontraumatic), left hand	Muscle disorders
M62149	Other rupture of muscle (nontraumatic), unspecified hand	Muscle disorders
M62151	Other rupture of muscle (nontraumatic), right thigh	Muscle disorders
M62152	Other rupture of muscle (nontraumatic), left thigh	Muscle disorders
M62159	Other rupture of muscle (nontraumatic), unspecified thigh	Muscle disorders
M62161	Other rupture of muscle (nontraumatic), right lower leg	Muscle disorders
M62162	Other rupture of muscle (nontraumatic), left lower leg	Muscle disorders
M62169	Other rupture of muscle (nontraumatic), unspecified lower leg	Muscle disorders
M62171	Other rupture of muscle (nontraumatic), right ankle and foot	Muscle disorders
M62172	Other rupture of muscle (nontraumatic), left ankle and foot	Muscle disorders
M62179	Other rupture of muscle (nontraumatic), unspecified ankle and foot	Muscle disorders
M6218	Other rupture of muscle (nontraumatic), other site	Muscle disorders
M6220	Nontraumatic ischemic infarction of muscle, unspecified site	Muscle disorders
M62211	Nontraumatic ischemic infarction of muscle, right shoulder	Muscle disorders
M62212	Nontraumatic ischemic infarction of muscle, left shoulder	Muscle disorders
M62219	Nontraumatic ischemic infarction of muscle, unspecified shoulder	Muscle disorders
M62221	Nontraumatic ischemic infarction of muscle, right upper arm	Muscle disorders
M62222	Nontraumatic ischemic infarction of muscle, left upper arm	Muscle disorders
M62229	Nontraumatic ischemic infarction of muscle, unspecified upper arm	Muscle disorders
M62231	Nontraumatic ischemic infarction of muscle, right forearm	Muscle disorders
M62232	Nontraumatic ischemic infarction of muscle, left forearm	Muscle disorders
M62239	Nontraumatic ischemic infarction of muscle, unspecified forearm	Muscle disorders
M62241	Nontraumatic ischemic infarction of muscle, right hand	Muscle disorders
M62242	Nontraumatic ischemic infarction of muscle, left hand	Muscle disorders
M62249	Nontraumatic ischemic infarction of muscle, unspecified hand	Muscle disorders
M62251	Nontraumatic ischemic infarction of muscle, right thigh	Muscle disorders
M62252	Nontraumatic ischemic infarction of muscle, left thigh	Muscle disorders
M62259	Nontraumatic ischemic infarction of muscle, unspecified thigh	Muscle disorders
M62261	Nontraumatic ischemic infarction of muscle, right lower leg	Muscle disorders
M62262	Nontraumatic ischemic infarction of muscle, left lower leg	Muscle disorders
M62269	Nontraumatic ischemic infarction of muscle, unspecified lower leg	Muscle disorders
M62271	Nontraumatic ischemic infarction of muscle, right ankle and foot	Muscle disorders
M62272	Nontraumatic ischemic infarction of muscle, left ankle and foot	Muscle disorders
M62279	Nontraumatic ischemic infarction of muscle, unspecified ankle and foot	Muscle disorders
M6228	Nontraumatic ischemic infarction of muscle, other site	Muscle disorders
M623	Immobility syndrome (paraplegic)	Muscle disorders
M6240	Contracture of muscle, unspecified site	Muscle disorders
M62411	Contracture of muscle, right shoulder	Muscle disorders
M62412	Contracture of muscle, left shoulder	Muscle disorders
M62419	Contracture of muscle, unspecified shoulder	Muscle disorders
M62421	Contracture of muscle, right upper arm	Muscle disorders
M62422	Contracture of muscle, left upper arm	Muscle disorders
M62429	Contracture of muscle, unspecified upper arm	Muscle disorders
M62431	Contracture of muscle, right forearm	Muscle disorders
M62432	Contracture of muscle, left forearm	Muscle disorders
M62439	Contracture of muscle, unspecified forearm	Muscle disorders
M62441	Contracture of muscle, right hand	Muscle disorders
M62442	Contracture of muscle, left hand	Muscle disorders
M62449	Contracture of muscle, unspecified hand	Muscle disorders
M62451	Contracture of muscle, right thigh	Muscle disorders
M62452	Contracture of muscle, left thigh	Muscle disorders
M62459	Contracture of muscle, unspecified thigh	Muscle disorders
M62461	Contracture of muscle, right lower leg	Muscle disorders
M62462	Contracture of muscle, left lower leg	Muscle disorders
M62469	Contracture of muscle, unspecified lower leg	Muscle disorders
M62471	Contracture of muscle, right ankle and foot	Muscle disorders
M62472	Contracture of muscle, left ankle and foot	Muscle disorders
M62479	Contracture of muscle, unspecified ankle and foot	Muscle disorders
M6248	Contracture of muscle, other site	Muscle disorders
M6249	Contracture of muscle, multiple sites	Muscle disorders
M6250	Muscle wasting and atrophy, not elsewhere classified, unspecified site	Muscle disorders
M62511	Muscle wasting and atrophy, not elsewhere classified, right shoulder	Muscle disorders
M62512	Muscle wasting and atrophy, not elsewhere classified, left shoulder	Muscle disorders
M62519	Muscle wasting and atrophy, not elsewhere classified, unspecified shoulder	Muscle disorders
M62521	Muscle wasting and atrophy, not elsewhere classified, right upper arm	Muscle disorders

M62522	Muscle wasting and atrophy, not elsewhere classified, left upper arm	Muscle disorders
M62529	Muscle wasting and atrophy, not elsewhere classified, unspecified upper arm	Muscle disorders
M62531	Muscle wasting and atrophy, not elsewhere classified, right forearm	Muscle disorders
M62532	Muscle wasting and atrophy, not elsewhere classified, left forearm	Muscle disorders
M62539	Muscle wasting and atrophy, not elsewhere classified, unspecified forearm	Muscle disorders
M62541	Muscle wasting and atrophy, not elsewhere classified, right hand	Muscle disorders
M62542	Muscle wasting and atrophy, not elsewhere classified, left hand	Muscle disorders
M62549	Muscle wasting and atrophy, not elsewhere classified, unspecified hand	Muscle disorders
M62551	Muscle wasting and atrophy, not elsewhere classified, right thigh	Muscle disorders
M62552	Muscle wasting and atrophy, not elsewhere classified, left thigh	Muscle disorders
M62559	Muscle wasting and atrophy, not elsewhere classified, unspecified thigh	Muscle disorders
M62561	Muscle wasting and atrophy, not elsewhere classified, right lower leg	Muscle disorders
M62562	Muscle wasting and atrophy, not elsewhere classified, left lower leg	Muscle disorders
M62569	Muscle wasting and atrophy, not elsewhere classified, unspecified lower leg	Muscle disorders
M62571	Muscle wasting and atrophy, not elsewhere classified, right ankle and foot	Muscle disorders
M62572	Muscle wasting and atrophy, not elsewhere classified, left ankle and foot	Muscle disorders
M62579	Muscle wasting and atrophy, not elsewhere classified, unspecified ankle and foot	Muscle disorders
M6258	Muscle wasting and atrophy, not elsewhere classified, other site	Muscle disorders
M6259	Muscle wasting and atrophy, not elsewhere classified, multiple sites	Muscle disorders
M6281	Muscle weakness (generalized)	Muscle disorders
M6282	Rhabdomyolysis	Muscle disorders
M62831	Muscle spasm of calf	Muscle disorders
M62838	Other muscle spasm	Muscle disorders
M6284	Sarcopenia	Muscle disorders
M6289	Other specified disorders of muscle	Muscle disorders
M629	Disorder of muscle, unspecified	Muscle disorders
M6380	Disorders of muscle in diseases classified elsewhere, unspecified site	Muscle disorders
M63811	Disorders of muscle in diseases classified elsewhere, right shoulder	Muscle disorders
M63812	Disorders of muscle in diseases classified elsewhere, left shoulder	Muscle disorders
M63819	Disorders of muscle in diseases classified elsewhere, unspecified shoulder	Muscle disorders
M63821	Disorders of muscle in diseases classified elsewhere, right upper arm	Muscle disorders
M63822	Disorders of muscle in diseases classified elsewhere, left upper arm	Muscle disorders
M63829	Disorders of muscle in diseases classified elsewhere, unspecified upper arm	Muscle disorders
M63831	Disorders of muscle in diseases classified elsewhere, right forearm	Muscle disorders
M63832	Disorders of muscle in diseases classified elsewhere, left forearm	Muscle disorders
M63839	Disorders of muscle in diseases classified elsewhere, unspecified forearm	Muscle disorders
M63841	Disorders of muscle in diseases classified elsewhere, right hand	Muscle disorders
M63842	Disorders of muscle in diseases classified elsewhere, left hand	Muscle disorders
M63849	Disorders of muscle in diseases classified elsewhere, unspecified hand	Muscle disorders
M63851	Disorders of muscle in diseases classified elsewhere, right thigh	Muscle disorders
M63852	Disorders of muscle in diseases classified elsewhere, left thigh	Muscle disorders
M63859	Disorders of muscle in diseases classified elsewhere, unspecified thigh	Muscle disorders
M63861	Disorders of muscle in diseases classified elsewhere, right lower leg	Muscle disorders
M63862	Disorders of muscle in diseases classified elsewhere, left lower leg	Muscle disorders
M63869	Disorders of muscle in diseases classified elsewhere, unspecified lower leg	Muscle disorders
M63871	Disorders of muscle in diseases classified elsewhere, right ankle and foot	Muscle disorders
M63872	Disorders of muscle in diseases classified elsewhere, left ankle and foot	Muscle disorders
M63879	Disorders of muscle in diseases classified elsewhere, unspecified ankle and foot	Muscle disorders
M6388	Disorders of muscle in diseases classified elsewhere, other site	Muscle disorders
M6389	Disorders of muscle in diseases classified elsewhere, multiple sites	Muscle disorders
**Renal system disorders**		
N170	Acute kidney failure with tubular necrosis	Renal failure
N171	Acute kidney failure with acute cortical necrosis	Renal failure
N172	Acute kidney failure with medullary necrosis	Renal failure
N178	Other acute kidney failure	Renal failure
N179	Acute kidney failure, unspecified	Renal failure
N19	Unspecified kidney failure	Renal failure
N181	Chronic kidney disease, stage 1	Chronic Kidney Disease
N182	Chronic kidney disease, stage 2 (mild)	Chronic Kidney Disease
N183	Chronic kidney disease, stage 3 (moderate)	Chronic Kidney Disease
N1830	Chronic kidney disease, stage 3 unspecified	Chronic Kidney Disease
N1831	Chronic kidney disease, stage 3a	Chronic Kidney Disease
N1832	Chronic kidney disease, stage 3b	Chronic Kidney Disease
N184	Chronic kidney disease, stage 4 (severe)	Chronic Kidney Disease
N185	Chronic kidney disease, stage 5	Chronic Kidney Disease
N186	End stage renal disease	Chronic Kidney Disease
N189	Chronic kidney disease, unspecified	Chronic Kidney Disease
R880	Cloudy (hemodialysis) (peritoneal) dialysis effluent	Chronic Kidney Disease
**Gastro-intestinal system disorders**		

K929	Other specified and unspecified gastrointestinal disorders	Gastrointestinal and esophageal
K580	Irritable bowel syndrome with diarrhea	Gastrointestinal and esophageal
K581	Irritable bowel syndrome with constipation	Gastrointestinal and esophageal
K582	Mixed irritable bowel syndrome	Gastrointestinal and esophageal
K588	Other irritable bowel syndrome	Gastrointestinal and esophageal
K589	Irritable bowel syndrome without diarrhea	Gastrointestinal and esophageal
K5900	Constipation, unspecified	Gastrointestinal and esophageal
K5901	Slow transit constipation	Gastrointestinal and esophageal
K5902	Outlet dysfunction constipation	Gastrointestinal and esophageal
K5903	Drug induced constipation	Gastrointestinal and esophageal
K5904	Chronic idiopathic constipation	Gastrointestinal and esophageal
K5909	Other constipation	Gastrointestinal and esophageal
K591	Functional diarrhea	Gastrointestinal and esophageal
K592	Neurogenic bowel, not elsewhere classified	Gastrointestinal and esophageal
K598	Other specified functional intestinal disorders	Gastrointestinal and esophageal
K5989	Other specified functional intestinal disorders	Gastrointestinal and esophageal
K599	Functional intestinal disorder, unspecified	Gastrointestinal and esophageal
K929	Disease of digestive system, unspecified	Gastrointestinal and esophageal
K200	Eosinophilic esophagitis	Gastrointestinal and esophageal
K208	Other esophagitis	Gastrointestinal and esophageal
K2080	Other esophagitis without bleeding	Gastrointestinal and esophageal
K2081	Other esophagitis with bleeding	Gastrointestinal and esophageal
K209	Esophagitis, unspecified	Gastrointestinal and esophageal
K2090	Esophagitis, unspecified without bleeding	Gastrointestinal and esophageal
K2091	Esophagitis, unspecified with bleeding	Gastrointestinal and esophageal
K210	Gastro-esophageal reflux disease with esophagitis	Gastrointestinal and esophageal
K2100	Gastro-esophageal reflux disease with esophagitis, without bleeding	Gastrointestinal and esophageal
K2101	Gastro-esophageal reflux disease with esophagitis, with bleeding	Gastrointestinal and esophageal
K219	Gastro-esophageal reflux disease without esophagitis	Gastrointestinal and esophageal
K220	Achalasia of cardia	Gastrointestinal and esophageal
K2210	Ulcer of esophagus without bleeding	Gastrointestinal and esophageal
K2211	Ulcer of esophagus with bleeding	Gastrointestinal and esophageal
K222	Esophageal obstruction	Gastrointestinal and esophageal
K223	Perforation of esophagus	Gastrointestinal and esophageal
K224	Dyskinesia of esophagus	Gastrointestinal and esophageal
K225	Diverticulum of esophagus, acquired	Gastrointestinal and esophageal
K226	Gastro-esophageal laceration-hemorrhage syndrome	Gastrointestinal and esophageal
K228	Other specified diseases of esophagus	Gastrointestinal and esophageal
K2289	Other specified disease of esophagus	Gastrointestinal and esophageal
K229	Disease of esophagus, unspecified	Gastrointestinal and esophageal
K23	Disorders of esophagus in diseases classified elsewhere	Gastrointestinal and esophageal
**Neurological disorders**		
G933	Postviral fatigue syndrome	Neurological conditions
F05	Delirium or Encephalopathy	Neurological conditions
R40.0	Delirium or Encephalopathy	Neurological conditions
R41	Delirium or Encephalopathy	Neurological conditions
R44	Delirium or Encephalopathy	Neurological conditions
F01	Dementia	Neurological conditions
F02	Dementia	Neurological conditions
F03	Dementia	Neurological conditions
G31	Dementia	Neurological conditions
A85	Encephalitis	Neurological conditions
A86	Encephalitis	Neurological conditions
G04	Encephalitis	Neurological conditions
G05	Encephalitis	Neurological conditions
R29	Encephalitis	Neurological conditions
R26	Ataxia / Trouble walking	Neurological conditions
R27	Ataxia / Trouble walking	Neurological conditions
G26	Ataxia / Trouble walking	Neurological conditions
G50	Peripheral Nerve Disorders	Neurological conditions
G51	Peripheral Nerve Disorders	Neurological conditions
G52	Peripheral Nerve Disorders	Neurological conditions
G53	Peripheral Nerve Disorders	Neurological conditions
G54	Peripheral Nerve Disorders	Neurological conditions
G55	Peripheral Nerve Disorders	Neurological conditions
G56	Peripheral Nerve Disorders	Neurological conditions
G57	Peripheral Nerve Disorders	Neurological conditions
G58	Peripheral Nerve Disorders	Neurological conditions
G59	Peripheral Nerve Disorders	Neurological conditions

G61	Peripheral Nerve Disorders	Neurological conditions
G62	Peripheral Nerve Disorders	Neurological conditions
G64	Peripheral Nerve Disorders	Neurological conditions
G65	Peripheral Nerve Disorders	Neurological conditions
R438	Other disturbances of smell and taste	Smell and taste disturbances
R439	Unspecified disturbances of smell and taste	Smell and taste disturbances
R43	Disturbances of smell and taste (All sub-codes)	Smell and taste disturbances
G40	Epilepsy and recurrent seizures	Seizures
H53	Visual disturbances	Ophthalmologic conditions
H54	Visual impairment including blindness (binocular or monocular)	Ophthalmologic conditions
G21	Secondary parkinsonism	Parkinsonism
G24	Dystonia	Parkinsonism
G25	Other extrapyramidal and movement disorders	Parkinsonism
G90	Disorders of autonomic nervous system	Autonomic disorders
G43	Migraine	Headache
G44	Other headache syndromes	Headache
R51	Headache	Headache
**Skin and subcutaneous system disorders**		
L209	Atopic dermatitis	Skin disorders
L309	Eczema	Skin disorders
L501	Urticaria	Skin disorders
L508	Other Urticaria	Skin disorders
B001	Herpes viral vesicular dermatitis	Skin disorders
B09	Unspecified viral infection characterized by skin and mucous membrane lesions	Skin disorders
R21	ash and other nonspecific skin eruption	Skin disorders
R23	Cyanosis	Skin disorders
T691XXA	Chilblains	Skin disorders
L63	Alopecia areata	Alopecia
L65	Other nonscarring hair loss	Alopecia
**Endocrine system disorders**		
E1010	Type 1 diabetes mellitus with ketoacidosis without coma	Type 1 diabetes
E1011	Type 1 diabetes mellitus with ketoacidosis with coma	Type 1 diabetes
E1021	Type 1 diabetes mellitus with diabetic nephropathy	Type 1 diabetes
E1022	Type 1 diabetes mellitus with diabetic chronic kidney disease	Type 1 diabetes
E1029	Type 1 diabetes mellitus with other diabetic kidney complication	Type 1 diabetes
E10311	Type 1 diabetes mellitus with unspecified diabetic retinopathy with macular edema	Type 1 diabetes
E10319	Type 1 diabetes mellitus with unspecified diabetic retinopathy without macular edema	Type 1 diabetes
E10321	Type 1 diabetes mellitus with mild nonproliferative diabetic retinopathy with macular	Type 1 diabetes
E103211	Type 1 diabetes mellitus with mild nonproliferative diabetic retinopathy with macular	Type 1 diabetes
E103212	Type 1 diabetes mellitus with mild nonproliferative diabetic retinopathy with macular	Type 1 diabetes
E103213	Type 1 diabetes mellitus with mild nonproliferative diabetic retinopathy with macular	Type 1 diabetes
E103219	Type 1 diabetes mellitus with mild nonproliferative diabetic retinopathy with macular	Type 1 diabetes
E10329	Type 1 diabetes mellitus with mild nonproliferative diabetic retinopathy without macular	Type 1 diabetes
E103291	Type 1 diabetes mellitus with mild nonproliferative diabetic retinopathy without macular	Type 1 diabetes
E103292	Type 1 diabetes mellitus with mild nonproliferative diabetic retinopathy without macular	Type 1 diabetes
E103293	Type 1 diabetes mellitus with mild nonproliferative diabetic retinopathy without macular	Type 1 diabetes
E103299	Type 1 diabetes mellitus with mild nonproliferative diabetic retinopathy without macular	Type 1 diabetes
E10331	Type 1 diabetes mellitus with moderate nonproliferative diabetic retinopathy with macular	Type 1 diabetes
E103311	Type 1 diabetes mellitus with moderate nonproliferative diabetic retinopathy with macular	Type 1 diabetes
E103312	Type 1 diabetes mellitus with moderate nonproliferative diabetic retinopathy with macular	Type 1 diabetes
E103313	Type 1 diabetes mellitus with moderate nonproliferative diabetic retinopathy with macular	Type 1 diabetes
E103319	Type 1 diabetes mellitus with moderate nonproliferative diabetic retinopathy with macular	Type 1 diabetes
E10339	Type 1 diabetes mellitus with moderate nonproliferative diabetic retinopathy without macular	Type 1 diabetes
E103391	Type 1 diabetes mellitus with moderate nonproliferative diabetic retinopathy without macular	Type 1 diabetes
E103392	Type 1 diabetes mellitus with moderate nonproliferative diabetic retinopathy without macular	Type 1 diabetes
E103393	Type 1 diabetes mellitus with moderate nonproliferative diabetic retinopathy without macular	Type 1 diabetes
E103399	Type 1 diabetes mellitus with moderate nonproliferative diabetic retinopathy without macular	Type 1 diabetes
E10341	Type 1 diabetes mellitus with severe nonproliferative diabetic retinopathy with macular	Type 1 diabetes
E103411	Type 1 diabetes mellitus with severe nonproliferative diabetic retinopathy with macular	Type 1 diabetes
E103412	Type 1 diabetes mellitus with severe nonproliferative diabetic retinopathy with macular	Type 1 diabetes
E103413	Type 1 diabetes mellitus with severe nonproliferative diabetic retinopathy with macular	Type 1 diabetes
E103419	Type 1 diabetes mellitus with severe nonproliferative diabetic retinopathy with macular	Type 1 diabetes
E10349	Type 1 diabetes mellitus with severe nonproliferative diabetic retinopathy without macular	Type 1 diabetes
E103491	Type 1 diabetes mellitus with severe nonproliferative diabetic retinopathy without macular	Type 1 diabetes
E103492	Type 1 diabetes mellitus with severe nonproliferative diabetic retinopathy without macular	Type 1 diabetes
E103493	Type 1 diabetes mellitus with severe nonproliferative diabetic retinopathy without macular	Type 1 diabetes
E103499	Type 1 diabetes mellitus with severe nonproliferative diabetic retinopathy without macular	Type 1 diabetes
E10351	Type 1 diabetes mellitus with proliferative diabetic retinopathy with macular edema	Type 1 diabetes
E103511	Type 1 diabetes mellitus with proliferative diabetic retinopathy with macular edema,	Type 1 diabetes

E103512	Type 1 diabetes mellitus with proliferative diabetic retinopathy with macular edema,	Type 1 diabetes
E103513	Type 1 diabetes mellitus with proliferative diabetic retinopathy with macular edema,	Type 1 diabetes
E103519	Type 1 diabetes mellitus with proliferative diabetic retinopathy with macular edema,	Type 1 diabetes
E103521	Type 1 diabetes mellitus with proliferative diabetic retinopathy with traction retinal	Type 1 diabetes
E103522	Type 1 diabetes mellitus with proliferative diabetic retinopathy with traction retinal	Type 1 diabetes
E103523	Type 1 diabetes mellitus with proliferative diabetic retinopathy with traction retinal	Type 1 diabetes
E103529	Type 1 diabetes mellitus with proliferative diabetic retinopathy with traction retinal	Type 1 diabetes
E103531	Type 1 diabetes mellitus with proliferative diabetic retinopathy with traction retinal	Type 1 diabetes
E103532	Type 1 diabetes mellitus with proliferative diabetic retinopathy with traction retinal	Type 1 diabetes
E103533	Type 1 diabetes mellitus with proliferative diabetic retinopathy with traction retinal	Type 1 diabetes
E103539	Type 1 diabetes mellitus with proliferative diabetic retinopathy with traction retinal	Type 1 diabetes
E103541	Type 1 diabetes mellitus with proliferative diabetic retinopathy with combined traction	Type 1 diabetes
E103542	Type 1 diabetes mellitus with proliferative diabetic retinopathy with combined traction	Type 1 diabetes
E103543	Type 1 diabetes mellitus with proliferative diabetic retinopathy with combined traction	Type 1 diabetes
E103549	Type 1 diabetes mellitus with proliferative diabetic retinopathy with combined traction	Type 1 diabetes
E103551	Type 1 diabetes mellitus with stable proliferative diabetic retinopathy, right eye	Type 1 diabetes
E103552	Type 1 diabetes mellitus with stable proliferative diabetic retinopathy, left eye	Type 1 diabetes
E103553	Type 1 diabetes mellitus with stable proliferative diabetic retinopathy, bilateral	Type 1 diabetes
E103559	Type 1 diabetes mellitus with stable proliferative diabetic retinopathy, unspecified eye	Type 1 diabetes
E10359	Type 1 diabetes mellitus with proliferative diabetic retinopathy without macular edema	Type 1 diabetes
E103591	Type 1 diabetes mellitus with proliferative diabetic retinopathy without macular edema,	Type 1 diabetes
E103592	Type 1 diabetes mellitus with proliferative diabetic retinopathy without macular edema,	Type 1 diabetes
E103593	Type 1 diabetes mellitus with proliferative diabetic retinopathy without macular edema,	Type 1 diabetes
E103599	Type 1 diabetes mellitus with proliferative diabetic retinopathy without macular edema,	Type 1 diabetes
E1036	Type 1 diabetes mellitus with diabetic cataract	Type 1 diabetes
E1037X1	Type 1 diabetes mellitus with diabetic macular edema, resolved following treatment,	Type 1 diabetes
E1037X2	Type 1 diabetes mellitus with diabetic macular edema, resolved following treatment,	Type 1 diabetes
E1037X3	Type 1 diabetes mellitus with diabetic macular edema, resolved following treatment,	Type 1 diabetes
E1037X9	Type 1 diabetes mellitus with diabetic macular edema, resolved following treatment,	Type 1 diabetes
E1039	Type 1 diabetes mellitus with other diabetic ophthalmic complication	Type 1 diabetes
E1040	Type 1 diabetes mellitus with diabetic neuropathy, unspecified	Type 1 diabetes
E1041	Type 1 diabetes mellitus with diabetic mononeuropathy	Type 1 diabetes
E1042	Type 1 diabetes mellitus with diabetic polyneuropathy	Type 1 diabetes
E1043	Type 1 diabetes mellitus with diabetic autonomic (poly)neuropathy	Type 1 diabetes
E1044	Type 1 diabetes mellitus with diabetic amyotrophy	Type 1 diabetes
E1049	Type 1 diabetes mellitus with other diabetic neurological complication	Type 1 diabetes
E1051	Type 1 diabetes mellitus with diabetic peripheral angiopathy without gangrene	Type 1 diabetes
E1052	Type 1 diabetes mellitus with diabetic peripheral angiopathy with gangrene	Type 1 diabetes
E1059	Type 1 diabetes mellitus with other circulatory complications	Type 1 diabetes
E10610	Type 1 diabetes mellitus with diabetic neuropathic arthropathy	Type 1 diabetes
E10618	Type 1 diabetes mellitus with other diabetic arthropathy	Type 1 diabetes
E10620	Type 1 diabetes mellitus with diabetic dermatitis	Type 1 diabetes
E10621	Type 1 diabetes mellitus with foot ulcer	Type 1 diabetes
E10622	Type 1 diabetes mellitus with other skin ulcer	Type 1 diabetes
E10628	Type 1 diabetes mellitus with other skin complications	Type 1 diabetes
E10630	Type 1 diabetes mellitus with periodontal disease	Type 1 diabetes
E10638	Type 1 diabetes mellitus with other oral complications	Type 1 diabetes
E10641	Type 1 diabetes mellitus with hypoglycemia with coma	Type 1 diabetes
E10649	Type 1 diabetes mellitus with hypoglycemia without coma	Type 1 diabetes
E1065	Type 1 diabetes mellitus with hyperglycemia	Type 1 diabetes
E1069	Type 1 diabetes mellitus with other specified complication	Type 1 diabetes
E108	Type 1 diabetes mellitus with unspecified complications	Type 1 diabetes
E109	Type 1 diabetes mellitus without complications	Type 1 diabetes
E1100	Type 2 diabetes mellitus with hyperosmolarity without nonketotic hyperglycemic-hyperosmolar coma (NKHHC)	Type 2 diabetes
E1101	Type 2 diabetes mellitus with hyperosmolarity with coma	Type 2 diabetes
E1110	Type 2 diabetes mellitus with ketoacidosis without coma	Type 2 diabetes
E1111	Type 2 diabetes mellitus with ketoacidosis with coma	Type 2 diabetes
E1121	Type 2 diabetes mellitus with diabetic nephropathy	Type 2 diabetes
E1122	Type 2 diabetes mellitus with diabetic chronic kidney disease	Type 2 diabetes
E1129	Type 2 diabetes mellitus with other diabetic kidney complication	Type 2 diabetes
E11311	Type 2 diabetes mellitus with unspecified diabetic retinopathy with macular edema	Type 2 diabetes
E11319	Type 2 diabetes mellitus with unspecified diabetic retinopathy without macular edema	Type 2 diabetes
E11321	Type 2 diabetes mellitus with mild nonproliferative diabetic retinopathy with macular	Type 2 diabetes
E113211	Type 2 diabetes mellitus with mild nonproliferative diabetic retinopathy with macular	Type 2 diabetes
E113212	Type 2 diabetes mellitus with mild nonproliferative diabetic retinopathy with macular	Type 2 diabetes
E113213	Type 2 diabetes mellitus with mild nonproliferative diabetic retinopathy with macular	Type 2 diabetes
E113219	Type 2 diabetes mellitus with mild nonproliferative diabetic retinopathy with macular	Type 2 diabetes
E11329	Type 2 diabetes mellitus with mild nonproliferative diabetic retinopathy without macular	Type 2 diabetes

E113291	Type 2 diabetes mellitus with mild nonproliferative diabetic retinopathy without macular	Type 2 diabetes
E113292	Type 2 diabetes mellitus with mild nonproliferative diabetic retinopathy without macular	Type 2 diabetes
E113293	Type 2 diabetes mellitus with mild nonproliferative diabetic retinopathy without macular	Type 2 diabetes
E113299	Type 2 diabetes mellitus with mild nonproliferative diabetic retinopathy without macular	Type 2 diabetes
E11331	Type 2 diabetes mellitus with moderate nonproliferative diabetic retinopathy with macular	Type 2 diabetes
E113311	Type 2 diabetes mellitus with moderate nonproliferative diabetic retinopathy with macular	Type 2 diabetes
E113312	Type 2 diabetes mellitus with moderate nonproliferative diabetic retinopathy with macular	Type 2 diabetes
E113313	Type 2 diabetes mellitus with moderate nonproliferative diabetic retinopathy with macular	Type 2 diabetes
E113319	Type 2 diabetes mellitus with moderate nonproliferative diabetic retinopathy with macular	Type 2 diabetes
E11339	Type 2 diabetes mellitus with moderate nonproliferative diabetic retinopathy without macular	Type 2 diabetes
E113391	Type 2 diabetes mellitus with moderate nonproliferative diabetic retinopathy without macular	Type 2 diabetes
E113392	Type 2 diabetes mellitus with moderate nonproliferative diabetic retinopathy without macular	Type 2 diabetes
E113393	Type 2 diabetes mellitus with moderate nonproliferative diabetic retinopathy without macular	Type 2 diabetes
E113399	Type 2 diabetes mellitus with moderate nonproliferative diabetic retinopathy without macular	Type 2 diabetes
E11341	Type 2 diabetes mellitus with severe nonproliferative diabetic retinopathy with macular	Type 2 diabetes
E113411	Type 2 diabetes mellitus with severe nonproliferative diabetic retinopathy with macular	Type 2 diabetes
E113412	Type 2 diabetes mellitus with severe nonproliferative diabetic retinopathy with macular	Type 2 diabetes
E113413	Type 2 diabetes mellitus with severe nonproliferative diabetic retinopathy with macular	Type 2 diabetes
E113419	Type 2 diabetes mellitus with severe nonproliferative diabetic retinopathy with macular	Type 2 diabetes
E11349	Type 2 diabetes mellitus with severe nonproliferative diabetic retinopathy without macular	Type 2 diabetes
E113491	Type 2 diabetes mellitus with severe nonproliferative diabetic retinopathy without macular	Type 2 diabetes
E113492	Type 2 diabetes mellitus with severe nonproliferative diabetic retinopathy without macular	Type 2 diabetes
E113493	Type 2 diabetes mellitus with severe nonproliferative diabetic retinopathy without macular	Type 2 diabetes
E113499	Type 2 diabetes mellitus with severe nonproliferative diabetic retinopathy without macular	Type 2 diabetes
E11351	Type 2 diabetes mellitus with proliferative diabetic retinopathy with macular edema	Type 2 diabetes
E113511	Type 2 diabetes mellitus with proliferative diabetic retinopathy with macular edema,	Type 2 diabetes
E113512	Type 2 diabetes mellitus with proliferative diabetic retinopathy with macular edema,	Type 2 diabetes
E113513	Type 2 diabetes mellitus with proliferative diabetic retinopathy with macular edema,	Type 2 diabetes
E113519	Type 2 diabetes mellitus with proliferative diabetic retinopathy with macular edema,	Type 2 diabetes
E113521	Type 2 diabetes mellitus with proliferative diabetic retinopathy with traction retinal	Type 2 diabetes
E113522	Type 2 diabetes mellitus with proliferative diabetic retinopathy with traction retinal	Type 2 diabetes
E113523	Type 2 diabetes mellitus with proliferative diabetic retinopathy with traction retinal	Type 2 diabetes
E113529	Type 2 diabetes mellitus with proliferative diabetic retinopathy with traction retinal	Type 2 diabetes
E113531	Type 2 diabetes mellitus with proliferative diabetic retinopathy with traction retinal	Type 2 diabetes
E113532	Type 2 diabetes mellitus with proliferative diabetic retinopathy with traction retinal	Type 2 diabetes
E113533	Type 2 diabetes mellitus with proliferative diabetic retinopathy with traction retinal	Type 2 diabetes
E113539	Type 2 diabetes mellitus with proliferative diabetic retinopathy with traction retinal	Type 2 diabetes
E113541	Type 2 diabetes mellitus with proliferative diabetic retinopathy with combined traction	Type 2 diabetes
E113542	Type 2 diabetes mellitus with proliferative diabetic retinopathy with combined traction	Type 2 diabetes
E113543	Type 2 diabetes mellitus with proliferative diabetic retinopathy with combined traction	Type 2 diabetes
E113549	Type 2 diabetes mellitus with proliferative diabetic retinopathy with combined traction	Type 2 diabetes
E113551	Type 2 diabetes mellitus with stable proliferative diabetic retinopathy, right eye	Type 2 diabetes
E113552	Type 2 diabetes mellitus with stable proliferative diabetic retinopathy, left eye	Type 2 diabetes
E113553	Type 2 diabetes mellitus with stable proliferative diabetic retinopathy, bilateral	Type 2 diabetes
E113559	Type 2 diabetes mellitus with stable proliferative diabetic retinopathy, unspecified eye	Type 2 diabetes
E11359	Type 2 diabetes mellitus with proliferative diabetic retinopathy without macular edema	Type 2 diabetes
E113591	Type 2 diabetes mellitus with proliferative diabetic retinopathy without macular edema,	Type 2 diabetes
E113592	Type 2 diabetes mellitus with proliferative diabetic retinopathy without macular edema,	Type 2 diabetes
E113593	Type 2 diabetes mellitus with proliferative diabetic retinopathy without macular edema,	Type 2 diabetes
E113599	Type 2 diabetes mellitus with proliferative diabetic retinopathy without macular edema,	Type 2 diabetes
E1136	Type 2 diabetes mellitus with diabetic cataract	Type 2 diabetes
E1137X1	Type 2 diabetes mellitus with diabetic macular edema, resolved following treatment,	Type 2 diabetes
E1137X2	Type 2 diabetes mellitus with diabetic macular edema, resolved following treatment,	Type 2 diabetes
E1137X3	Type 2 diabetes mellitus with diabetic macular edema, resolved following treatment,	Type 2 diabetes
E1137X9	Type 2 diabetes mellitus with diabetic macular edema, resolved following treatment,	Type 2 diabetes
E1139	Type 2 diabetes mellitus with other diabetic ophthalmic complication	Type 2 diabetes
E1140	Type 2 diabetes mellitus with diabetic neuropathy, unspecified	Type 2 diabetes
E1141	Type 2 diabetes mellitus with diabetic mononeuropathy	Type 2 diabetes
E1142	Type 2 diabetes mellitus with diabetic polyneuropathy	Type 2 diabetes
E1143	Type 2 diabetes mellitus with diabetic autonomic (poly)neuropathy	Type 2 diabetes
E1144	Type 2 diabetes mellitus with diabetic amyotrophy	Type 2 diabetes
E1149	Type 2 diabetes mellitus with other diabetic neurological complication	Type 2 diabetes
I99	Other specified and unspecified circulatory disease	Type 2 diabetes
E1151	Type 2 diabetes mellitus with diabetic peripheral angiopathy without gangrene	Type 2 diabetes
E1152	Type 2 diabetes mellitus with diabetic peripheral angiopathy with gangrene	Type 2 diabetes
E1159	Type 2 diabetes mellitus with other circulatory complications	Type 2 diabetes
E11610	Type 2 diabetes mellitus with diabetic neuropathic arthropathy	Type 2 diabetes
E11618	Type 2 diabetes mellitus with other diabetic arthropathy	Type 2 diabetes
E11620	Type 2 diabetes mellitus with diabetic dermatitis	Type 2 diabetes

E11621	Type 2 diabetes mellitus with foot ulcer	Type 2 diabetes
E11622	Type 2 diabetes mellitus with other skin ulcer	Type 2 diabetes
E11628	Type 2 diabetes mellitus with other skin complications	Type 2 diabetes
E11630	Type 2 diabetes mellitus with periodontal disease	Type 2 diabetes
E11638	Type 2 diabetes mellitus with other oral complications	Type 2 diabetes
E11641	Type 2 diabetes mellitus with hypoglycemia with coma	Type 2 diabetes
E11649	Type 2 diabetes mellitus with hypoglycemia without coma	Type 2 diabetes
E1165	Type 2 diabetes mellitus with hyperglycemia	Type 2 diabetes
E1169	Type 2 diabetes mellitus with other specified complication	Type 2 diabetes
E118	Type 2 diabetes mellitus with unspecified complications	Type 2 diabetes
E119	Type 2 diabetes mellitus without complications	Type 2 diabetes
E1151	Type 2 diabetes mellitus with diabetic peripheral angiopathy without gangrene	Type 2 diabetes
E1152	Type 2 diabetes mellitus with diabetic peripheral angiopathy with gangrene	Type 2 diabetes
E1159	Type 2 diabetes mellitus with other circulatory complications	Type 2 diabetes
E11610	Type 2 diabetes mellitus with diabetic neuropathic arthropathy	Type 2 diabetes
E11618	Type 2 diabetes mellitus with other diabetic arthropathy	Type 2 diabetes
E11620	Type 2 diabetes mellitus with diabetic dermatitis	Type 2 diabetes
E11621	Type 2 diabetes mellitus with foot ulcer	Type 2 diabetes
E11622	Type 2 diabetes mellitus with other skin ulcer	Type 2 diabetes
E11628	Type 2 diabetes mellitus with other skin complications	Type 2 diabetes
E11630	Type 2 diabetes mellitus with periodontal disease	Type 2 diabetes
E11638	Type 2 diabetes mellitus with other oral complications	Type 2 diabetes
E11641	Type 2 diabetes mellitus with hypoglycemia with coma	Type 2 diabetes
E11649	Type 2 diabetes mellitus with hypoglycemia without coma	Type 2 diabetes
E1165	Type 2 diabetes mellitus with hyperglycemia	Type 2 diabetes
E1169	Type 2 diabetes mellitus with other specified complication	Type 2 diabetes
E118	Type 2 diabetes mellitus with unspecified complications	Type 2 diabetes
E119	Type 2 diabetes mellitus without complications	Type 2 diabetes
**Mental and behavioral disorders**		
F064	Anxiety disorder due to known physiological condition	Anxiety and fear-related
F4000	Agoraphobia, unspecified	Anxiety and fear-related
F4001	Agoraphobia with panic disorder	Anxiety and fear-related
F4002	Agoraphobia without panic disorder	Anxiety and fear-related
F4010	Social phobia, unspecified	Anxiety and fear-related
F4011	Social phobia, generalized	Anxiety and fear-related
F40228	Other natural environment type phobia	Anxiety and fear-related
F40230	Fear of blood	Anxiety and fear-related
F40231	Fear of injections and transfusions	Anxiety and fear-related
F40232	Fear of other medical care	Anxiety and fear-related
F40233	Fear of injury	Anxiety and fear-related
F40240	Claustrophobia	Anxiety and fear-related
F40248	Other situational type phobia	Anxiety and fear-related
F408	Other phobic anxiety disorders	Anxiety and fear-related
F409	Phobic anxiety disorder, unspecified	Anxiety and fear-related
F410	Panic disorder [episodic paroxysmal anxiety]	Anxiety and fear-related
F411	Generalized anxiety disorder	Anxiety and fear-related
F413	Other mixed anxiety disorders	Anxiety and fear-related
F418	Other specified anxiety disorders	Anxiety and fear-related
F419	Anxiety disorder, unspecified	Anxiety and fear-related
F930	Separation anxiety disorder of childhood	Anxiety and fear-related
F431	PTSD	Anxiety and fear-related
F304	Manic episode in full remission	Mood disorders
F3170	Bipolar disorder, currently in remission, most recent episode unspecified	Mood disorders
F3172	Bipolar disorder, in full remission, most recent episode hypomanic	Mood disorders
F3174	Bipolar disorder, in full remission, most recent episode manic	Mood disorders
F3176	Bipolar disorder, in full remission, most recent episode depressed	Mood disorders
F3178	Bipolar disorder, in full remission, most recent episode mixed	Mood disorders
F325	Major depressive disorder, single episode, in full remission	Mood disorders
F3340	Major depressive disorder, recurrent, in remission, unspecified	Mood disorders
F3342	Major depressive disorder, recurrent, in full remission	Mood disorders
F99	Other mental conditions and symptoms	Mood disorders
R450	Nervousness	Mood disorders
R451	Restlessness and agitation	Mood disorders
R452	Unhappiness	Mood disorders
R453	Demoralization and apathy	Mood disorders
R454	Irritability and anger	Mood disorders
R455	Hostility	Mood disorders
R456	Violent behavior	Mood disorders
R457	State of emotional shock and stress, unspecified	Mood disorders

R4581	Low self-esteem	Mood disorders
R4582	Worries	Mood disorders
R4583	Excessive crying of child, adolescent or adult	Mood disorders
R4584	Anhedonia	Mood disorders
R45850	Homicidal ideations	Mood disorders
R4586	Emotional lability	Mood disorders
R4587	Impulsiveness	Mood disorders
R4589	Other symptoms and signs involving emotional state	Mood disorders
R460	Very low level of personal hygiene	Mood disorders
R461	Bizarre personal appearance	Mood disorders
R462	Strange and inexplicable behavior	Mood disorders
R463	Overactivity	Mood disorders
R464	Slowness and poor responsiveness	Mood disorders
R465	Suspiciousness and marked evasiveness	Mood disorders
R466	Undue concern and preoccupation with stressful events	Mood disorders
R467	Verbosity and circumstantial detail obscuring reason for contact	Mood disorders
R4681	Obsessive-compulsive behavior	Mood disorders
R4689	Other symptoms and signs involving appearance and behavior	Mood disorders
F39	Other specified and unspecified mood disorders	Mood disorders
F0630	Mood disorder due to known physiological condition, unspecified	Mood disorders
F348	Other persistent mood [affective] disorders	Mood disorders
F3481	Disruptive mood dysregulation disorder	Mood disorders
F3489	Other specified persistent mood disorders	Mood disorders
F349	Persistent mood [affective] disorder, unspecified	Mood disorders
F39	Unspecified mood [affective] disorder	Mood disorders
F32	depressive episode	Mood disorders
F33	major depressive disorder	Mood disorders
F34	persistent mood disorder	Mood disorders
G4700	Insomnia, unspecified	Sleep disorders
G4701	Insomnia due to medical condition	Sleep disorders
G4709	Other insomnia	Sleep disorders
G4710	Hypersomnia, unspecified	Sleep disorders
G4711	Idiopathic hypersomnia with long sleep time	Sleep disorders
G4712	Idiopathic hypersomnia without long sleep time	Sleep disorders
G4713	Recurrent hypersomnia	Sleep disorders
G4714	Hypersomnia due to medical condition	Sleep disorders
G4719	Other hypersomnia	Sleep disorders
G4720	Circadian rhythm sleep disorder, unspecified type	Sleep disorders
G4721	Circadian rhythm sleep disorder, delayed sleep phase type	Sleep disorders
G4722	Circadian rhythm sleep disorder, advanced sleep phase type	Sleep disorders
G4723	Circadian rhythm sleep disorder, irregular sleep wake type	Sleep disorders
G4724	Circadian rhythm sleep disorder, free running type	Sleep disorders
G4725	Circadian rhythm sleep disorder, jet lag type	Sleep disorders
G4726	Circadian rhythm sleep disorder, shift work type	Sleep disorders
G4727	Circadian rhythm sleep disorder in conditions classified elsewhere	Sleep disorders
G4729	Other circadian rhythm sleep disorder	Sleep disorders
G4730	Sleep apnea, unspecified	Sleep disorders
G4731	Primary central sleep apnea	Sleep disorders
G4732	High altitude periodic breathing	Sleep disorders
G4733	Obstructive sleep apnea (adult) (pediatric)	Sleep disorders
G4734	Idiopathic sleep related nonobstructive alveolar hypoventilation	Sleep disorders
G4735	Congenital central alveolar hypoventilation syndrome	Sleep disorders
G4736	Sleep related hypoventilation in conditions classified elsewhere	Sleep disorders
G4737	Central sleep apnea in conditions classified elsewhere	Sleep disorders
G4739	Other sleep apnea	Sleep disorders
G47411	Narcolepsy with cataplexy	Sleep disorders
G47419	Narcolepsy without cataplexy	Sleep disorders
G47421	Narcolepsy in conditions classified elsewhere with cataplexy	Sleep disorders
G47429	Narcolepsy in conditions classified elsewhere without cataplexy	Sleep disorders
G4750	Parasomnia, unspecified	Sleep disorders
G4751	Confusional arousals	Sleep disorders
G4752	REM sleep behavior disorder	Sleep disorders
G4753	Recurrent isolated sleep paralysis	Sleep disorders
G4754	Parasomnia in conditions classified elsewhere	Sleep disorders
G4759	Other parasomnia	Sleep disorders
G4761	Periodic limb movement disorder	Sleep disorders
G4762	Sleep related leg cramps	Sleep disorders
G4763	Sleep related bruxism	Sleep disorders
G4769	Other sleep related movement disorders	Sleep disorders

G478	Other sleep disorders	Sleep disorders
G479	Sleep disorder, unspecified	Sleep disorders
R063	Periodic breathing	Sleep disorders
F1011	Alcohol abuse, in remission	substance use disorders
F1021	Alcohol dependence, in remission	substance use disorders
F1111	Opioid abuse, in remission	substance use disorders
F1121	Opioid dependence, in remission	substance use disorders
F1211	Cannabis abuse, in remission	substance use disorders
F1221	Cannabis dependence, in remission	substance use disorders
F1311	Sedative, hypnotic or anxiolytic abuse, in remission	substance use disorders
F1321	Sedative, hypnotic or anxiolytic dependence, in remission	substance use disorders
F1411	Cocaine abuse, in remission	substance use disorders
F1421	Cocaine dependence, in remission	substance use disorders
F1511	Other stimulant abuse, in remission	substance use disorders
F1521	Other stimulant dependence, in remission	substance use disorders
F1611	Hallucinogen abuse, in remission	substance use disorders
F1621	Hallucinogen dependence, in remission	substance use disorders
F17201	Nicotine dependence, unspecified, in remission	substance use disorders
F17211	Nicotine dependence, cigarettes, in remission	substance use disorders
F17221	Nicotine dependence, chewing tobacco, in remission	substance use disorders
F17291	Nicotine dependence, other tobacco product, in remission	substance use disorders
F1811	Inhalant abuse, in remission	substance use disorders
F1821	Inhalant dependence, in remission	substance use disorders
F1910	Other psychoactive substance abuse, uncomplicated	substance use disorders
F1911	Other psychoactive substance abuse, in remission	substance use disorders
F19120	Other psychoactive substance abuse with intoxication, uncomplicated	substance use disorders
F19121	Other psychoactive substance abuse with intoxication delirium	substance use disorders
F19122	Other psychoactive substance abuse with intoxication with perceptual disturbances	substance use disorders
F19129	Other psychoactive substance abuse with intoxication, unspecified	substance use disorders
F19130	Other psychoactive substance abuse with withdrawal, uncomplicated	substance use disorders
F19131	Other psychoactive substance abuse with withdrawal delirium	substance use disorders
F19132	Other psychoactive substance abuse with withdrawal with perceptual disturbance	substance use disorders
F19139	Other psychoactive substance abuse with withdrawal, unspecified	substance use disorders
F1914	Other psychoactive substance abuse with psychoactive substance-induced mood disorder	substance use disorders
F1916	Other psychoactive substance abuse with psychoactive substance-induced persisting amnestic disorder	substance use disorders
F1917	Other psychoactive substance abuse with psychoactive substance-induced persisting dementia	substance use disorders
F19181	Other psychoactive substance abuse with psychoactive substance-induced sexual dysfunction	substance use disorders
F19182	Other psychoactive substance abuse with psychoactive substance-induced sleep disorder	substance use disorders
F19188	Other psychoactive substance abuse with other psychoactive substance-induced disorder	substance use disorders
F1919	Other psychoactive substance abuse with unspecified psychoactive substance-induced disorder	substance use disorders
F1920	Other psychoactive substance dependence, uncomplicated	substance use disorders
F1921	Other psychoactive substance dependence, in remission	substance use disorders
F19220	Other psychoactive substance dependence with intoxication, uncomplicated	substance use disorders
F19221	Other psychoactive substance dependence with intoxication delirium	substance use disorders
F19222	Other psychoactive substance dependence with intoxication with perceptual disturbance	substance use disorders
F19229	Other psychoactive substance dependence with intoxication, unspecified	substance use disorders
F19230	Other psychoactive substance dependence with withdrawal, uncomplicated	substance use disorders
F19231	Other psychoactive substance dependence with withdrawal delirium	substance use disorders
F19232	Other psychoactive substance dependence with withdrawal with perceptual disturbance	substance use disorders
F19239	Other psychoactive substance dependence with withdrawal, unspecified	substance use disorders
F1924	Other psychoactive substance dependence with psychoactive substance-induced mood disorder	substance use disorders
F1926	Other psychoactive substance dependence with psychoactive substance-induced persisting amnestic disorder	substance use disorders
F1927	Other psychoactive substance dependence with psychoactive substance-induced persisting dementia	substance use disorders
F19281	Other psychoactive substance dependence with psychoactive substance-induced sexual dysfunction	substance use disorders
F19282	Other psychoactive substance dependence with psychoactive substance-induced sleep disorder	substance use disorders
F19288	Other psychoactive substance dependence with other psychoactive substance-induced disorder	substance use disorders
F1929	Other psychoactive substance dependence with unspecified psychoactive substance-induced disorder	substance use disorders
F1990	Other psychoactive substance use, unspecified, uncomplicated	substance use disorders
F19920	Other psychoactive substance use, unspecified with intoxication, uncomplicated	substance use disorders
F19921	Other psychoactive substance use, unspecified with intoxication with delirium	substance use disorders
F19922	Other psychoactive substance use, unspecified with intoxication with perceptual disturbance	substance use disorders
F19929	Other psychoactive substance use, unspecified with intoxication, unspecified	substance use disorders
F19930	Other psychoactive substance use, unspecified with withdrawal, uncomplicated	substance use disorders
F19931	Other psychoactive substance use, unspecified with withdrawal delirium	substance use disorders

F19932	Other psychoactive substance use, unspecified with withdrawal with perceptual disturbance	substance use disorders
F19939	Other psychoactive substance use, unspecified with withdrawal, unspecified	substance use disorders
F1994	Other psychoactive substance use, unspecified with psychoactive substance-induced mood disorder	substance use disorders
F1996	Other psychoactive substance use, unspecified with psychoactive substance-induced persisting amnestic disorder	substance use disorders
F1997	Other psychoactive substance use, unspecified with psychoactive substance-induced persisting dementia	substance use disorders
F19981	Other psychoactive substance use, unspecified with psychoactive substance-induced sexual dysfunction	substance use disorders
F19982	Other psychoactive substance use, unspecified with psychoactive substance-induced sleep disorder	substance use disorders
F19988	Other psychoactive substance use, unspecified with other psychoactive substance-induced disorder	substance use disorders
F1999	Other psychoactive substance use, unspecified with unspecified psychoactive substance-induced disorder	substance use disorders
