# Supplementary material for: Comparative risk of post-acute sequelae following SARS-CoV-2 or influenza virus infection: A retrospective cohort study among United States adults
Source: PLoS Med. 2025 Oct 9;22(10):e1004777. doi: 10.1371/journal.pmed.1004777 (PMC12551960; doi:10.1371/journal.pmed.1004777)
Supplement: S2 Table — (PDF) [file pmed.1004777.s005.pdf]

Table S2: Case counts under alternative categorization of continuous covariates.

Characteristic		Index infection	
		COVID-19	*Influenza*
		N=74,738	*N*=18,790
Age	18-24 years	3,626 (4.9)	2,486 (13.2)
	25-34 years	8,348 (11.2)	3,518 (18.7)
	35-49 years	16,746 (22.4)	4,834 (25.7)
	50-64 years	20,650 (27.6)	4,116 (21.9)
	65-74 years	12,302 (16.5)	2,038 (10.8)
	75-84 years	8,904 (11.9)	1,325 (7.1)
	≥85 years	4,162 (5.6)	473 (2.5)
Neighborhood deprivation index			
	1st quartile (most deprived)	19,058 (25.5)	4,404 (23.4)
	2nd quartile	18,810 (25.2)	4,659 (24.8)
	3rd quartile	18,654 (25.0)	4,608 (24.5)
	4th quartile (least deprived)	18,185 (24.3)	5,112 (27.2)
	Unknown	31 (0.0)	7 (0.0)
Charlson comorbidity index			
	0	36,285 (48.5)	10,992 (58.5)
	1	14,611 (19.5)	3,686 (19.6)
	2-3	12,005 (16.1)	2,209 (11.8)
	4-7	9,497 (12.7)	1,570 (8.4)
	≥8	2,340 (3.1)	333 (1.8)
Prior-year ambulatory healthcare utilization			
	0 visits	3,250 (4.3)	1,460 (7.8)
	1-5 visits	23,379 (31.3)	7,462 (39.7)
	6-15 visits	29,949 (40.1)	6,621 (35.2)
	16-25 visits	10,845 (14.5)	2,005 (10.7)
	26-40 visits	4,978 (6.7)	869 (4.6)
	≥41 visits	2,337 (3.1)	373 (2.0)

Eligible individuals were Kaiser Permanente Southern California healthcare plan members aged ≥18 years with a positive molecular test for SARS-CoV-2 or influenza between September 1, 2022 and December 31, 2023, who had been members of KPSC for ≥12 months preceding their index episode. We restricted analyses to individuals with accompanying acute respiratory illness diagnoses in any clinical setting between 7 days before or after the positive test.
