# Supplementary material for: Comparative risk of post-acute sequelae following SARS-CoV-2 or influenza virus infection: A retrospective cohort study among United States adults
Source: PLoS Med. 2025 Oct 9;22(10):e1004777. doi: 10.1371/journal.pmed.1004777 (PMC12551960; doi:10.1371/journal.pmed.1004777)
Supplement: S3 Table — (PDF) [file pmed.1004777.s006.pdf]

Table S3: Individual Charlson comorbidity categories among COVID-19 cases and influenza cases.

Condition	Index infection, *n* (%)	
	COVID-19	*Influenza*
	N=74,738	*N*=18,790
Myocardial infarction	2,871 (3.8)	430 (2.3)
Congestive heart failure	5,193 (6.9)	865 (4.6)
Peripheral vascular disease	15,766 (21.1)	2,399 (12.8)
Cerebrovascular disease	3,584 (4.8)	487 (2.6)
Dementia	2,419 (3.2)	295 (1.6)
Chronic pulmonary disease	13,815 (18.5)	3,606 (19.2)
Connective tissue disease/rheumatic disease	1,853 (2.5)	374 (2.0)
Peptic ulcer disease	474 (0.6)	72 (0.4)
Diabetes without complications	8,639 (11.6)	1,668 (8.9)
Diabetes with complications	10,273 (13.7)	1,699 (9.0)
Paraplegia and hemiplegia	804 (1.1)	107 (0.6)
Renal disease	9,235 (12.4)	1,414 (7.5)
Mild liver disease	4,656 (6.2)	867 (4.6)
Moderate or severe liver disease	406 (0.5)	55 (0.3)
Cancer	3,191 (4.3)	540 (2.9)
Metastatic carcinoma	1,265 (1.7)	206 (1.1)
AIDS/HIV	135 (0.2)	34 (0.2)
