# Supplementary material for: Comparative risk of post-acute sequelae following SARS-CoV-2 or influenza virus infection: A retrospective cohort study among United States adults
Source: PLoS Med. 2025 Oct 9;22(10):e1004777. doi: 10.1371/journal.pmed.1004777 (PMC12551960; doi:10.1371/journal.pmed.1004777)
Supplement: S4 Table — (PDF) [file pmed.1004777.s007.pdf]

Table S4: Cohort retention and frequency of post-acute sequelae managed in any clinical setting.

Outcome		Participants, *n* (%)			
		Within 31-90d		*Within 91-180d*	
		COVID-19	Influenza	COVID-19	Influenza
		N=72,745	*N*=18,395	*N*=71,238	*N*=17,990
Death/disenrollment before PAS observed					
	Cumulative deaths/disenrollments before start of follow-up period[1]	1,993 (2.7% of 74,738)	395 (2.1% of 18,790)	3,500 (4.7% of 74,738)	800 (4.3% of 18,790)
	Cumulative deaths before start of follow-up[1]	826 (1.1% of 74,738)	89 (0.5% of 18,790)	1,391 (1.9% of 74,738)	149 (0.8% of 18,790)
	Deaths/disenrollments occurring during follow-up period[2]	1,507 (2.1)	405 (2.2)	1,878 (2.6)	378 (2.1)
	Deaths occurring during follow-up period[2]	565 (0.8)	60 (0.3)	513 (0.7)	60 (0.3)
PAS—by category					
	Any	32,878 (45.2)	7,162 (38.9)	37,615 (52.8)	7,916 (44.0)
	Cardiopulmonary	14,614 (20.0)	2,839 (15.4)	17,134 (23.9)	2,923 (16.1)
	Hemolytic	2,320 (3.2)	423 (2.3)	2,681 (3.7)	377 (2.1)
	Respiratory	10,076 (13.8)	2,573 (13.9)	12,130 (16.9)	2,738 (15.1)
	Musculoskeletal	9,063 (12.4)	1,730 (9.3)	11,793 (16.4)	2,218 (12.3)
	Renal	4,466 (6.1)	806 (4.4)	5,074 (7.1)	787 (4.3)
	Gastrointestinal	4,719 (6.4)	1,030 (5.6)	5,804 (9.1)	1,075 (5.9)
	Neurological	6,538 (8.9)	1,301 (7.1)	8,453 (11.9)	1,492 (8.2)
	Skin	1,800 (2.5)	443 (2.4)	2,616 (3.6)	592 (3.3)
	Endocrine	9,706 (13.3)	1,843 (10.0)	11,161 (15.6)	1,966 (10.9)
	Mental health	9,284 (12.7)	2,185 (11.8)	11,150 (15.5)	2,368 (13.1)
PAS—by clinical setting					
	Any setting	32,878 (45.2)	7,162 (38.9)	37,615 (52.8)	7,916 (44.0)
	Ambulatory or higher-acuity setting	27,082 (37.1)	5,723 (31.0)	32,361 (45.4)	6,526 (36.2)
	Emergency department or higher-acuity setting	9,121 (12.4)	2,067 (11.2)	11,771 (16.5)	2,565 (14.2)
	Inpatient setting	2,078 (2.8)	335 (1.8)	2,226 (3.1)	376 (2.1)

PAS: post-acute sequelae.

[1]Figures indicate the number of all otherwise-eligible COVID-19 and influenza cases (included in Table 1) who died or disenrolled before the beginning of each follow-up period. Totals represented in column headings indicate the number of individuals who remained alive and enrolled at the beginning of each follow-up period (31 days and 91 days after index).

[2]Figures indicate the number of COVID-19 and influenza cases who died or disenrolled during the indicated follow-up periods, calculated as a proportion of the total of all individuals who were retained in follow-up at 31 days and 91 days after index (column heading totals).
