# Supplementary material for: Comparative risk of post-acute sequelae following SARS-CoV-2 or influenza virus infection: A retrospective cohort study among United States adults
Source: PLoS Med. 2025 Oct 9;22(10):e1004777. doi: 10.1371/journal.pmed.1004777 (PMC12551960; doi:10.1371/journal.pmed.1004777)
Supplement: S5 Table — (PDF) [file pmed.1004777.s008.pdf]

Table S5: Adjusted hazard ratios of any post-acute sequelae estimated using models with alternative handling of continuous covariates.

Framework	Acuity of care setting for PAS diagnoses	Adjusted hazard ratio (95% CI), COVID-19 cases compared to influenza cases[1]	
		Within 31-90 days	*Within 91-180 days*
Alternative binning (per **Table S2**) of continuous covariates			
	Any setting	1.03 (0.98, 1.09)	1.01 (0.96, 1.06)
	Ambulatory or higher-acuity setting	1.05 (0.99, 1.11)	1.01 (0.96, 1.06)
	Emergency department or higher-acuity setting	1.05 (0.96, 1.16)	1.08 (0.99, 1.18)
	Inpatient setting	1.29 (1.06, 1.57)	1.24 (1.03, 1.49)
Defining continuous covariates			
	Any setting	1.04 (0.99, 1.10)	1.03 (0.98, 1.08)
	Ambulatory or higher-acuity setting	1.06 (1.00, 1.12)	1.02 (0.97, 1.08)
	Emergency department or higher-acuity setting	1.05 (0.95, 1.16)	1.09 (0.99, 1.19)
	Inpatient setting	1.28 (1.05, 1.57)	1.26 (1.04, 1.52)

PAS: post-acute sequelae; CI: Confidence interval.

[1]Estimates are computed as adjusted hazards ratios via doubly-robust Cox proportional hazards models weighted to account for individuals' inverse probability of infection with their identified virus and the inverse of their probability of retention through each 30-day period after the index date. Covariates used in weighting models are included in the analysis model. We use the sandwich variance estimator to account for repeated observations of individuals across multiple 30-day periods.
