# Supplementary material for: Comparative risk of post-acute sequelae following SARS-CoV-2 or influenza virus infection: A retrospective cohort study among United States adults
Source: PLoS Med. 2025 Oct 9;22(10):e1004777. doi: 10.1371/journal.pmed.1004777 (PMC12551960; doi:10.1371/journal.pmed.1004777)
Supplement: S6 Table — (PDF) [file pmed.1004777.s009.pdf]

Table S6: Cumulative incidence of post-acute sequelae as new-onset conditions or exacerbations of existing conditions, diagnosed in any clinical setting.

Outcome		Cumulative incidence per 100 persons during follow-up period, by index infection[1]			
		Within 31-90d		*Within 91-180d*	
		COVID-19	Influenza	COVID-19	Influenza
PAS—exacerbation[2]					
	Cardiopulmonary	38.41	39.02	45.54	40.01
	Hemolytic	24.46	29.70	29.84	23.08
	Respiratory	21.97	22.77	26.92	24.32
	Musculoskeletal	19.82	16.63	25.02	20.41
	Renal	42.63	48.75	49.73	46.95
	Gastrointestinal	17.81	18.90	21.83	18.82
	Neurological	21.15	19.24	26.97	21.14
	Skin	6.76	7.20	9.14	8.39
	Endocrine	51.81	54.18	60.66	57.78
	Mental health	30.66	32.28	36.16	33.56
PAS—new onset[3]					
	Cardiopulmonary	4.20	3.24	5.53	3.89
	Hemolytic	0.94	0.60	1.09	0.81
	Respiratory	7.34	6.96	9.12	7.90
	Musculoskeletal	6.59	5.16	9.82	7.57
	Renal	0.80	0.48	1.05	0.66
	Gastrointestinal	2.92	2.58	3.87	3.05
	Neurological	3.86	3.33	5.56	4.33
	Skin	1.80	1.75	2.80	2.58
	Endocrine	0.68	0.49	0.94	0.76
	Mental health	3.50	3.35	5.09	4.59

PAS: post-acute sequelae.

[1]We present unadjusted cumulative incidence of each PAS outcome according to individuals' history of diagnosis or lack of diagnosis within each category.

[2]Analyses of exacerbation outcomes are limited to individuals with a history of any preceding diagnosis (prior to their index infection) with conditions included in the indicated PAS category.

[3]Analyses of new-onset PAS outcomes are limited to individuals without a history of any preceding diagnosis (prior to their index infection) with conditions included in the indicated PAS category
