# Supplementary material for: Comparative risk of post-acute sequelae following SARS-CoV-2 or influenza virus infection: A retrospective cohort study among United States adults
Source: PLoS Med. 2025 Oct 9;22(10):e1004777. doi: 10.1371/journal.pmed.1004777 (PMC12551960; doi:10.1371/journal.pmed.1004777)
Supplement: S7 Table — (PDF) [file pmed.1004777.s010.pdf]

Table S7: Adjusted hazard ratios of post-acute sequelae, subset by infecting virus lineage or period.

Characteristic	Stratum	Adjusted hazards ratio (95% CI), COVID-19 cases compared to influenza cases, according to severity of index episode[1]			
		Within 31-90 days		*Within 91-180 days*	
		Any severity	Inpatient managed	Any severity	Inpatient managed
Season					
	Season 1 (2022-23)	1.04 (0.98, 1.10)	1.30 (1.03, 1.65)	1.01 (0.95, 1.06)	1.18 (0.96, 1.45)
	Season 2 (2023-24)	1.02 (0.92, 1.13)	1.33 (0.96, 1.84)	1.03 (0.93, 1.14)	1.56 (1.14, 2.14)
Influenza comparator					
	Influenza A virus	1.04 (0.98, 1.09)	1.30 (1.06, 1.58)	1.02 (0.98, 1.07)	1.24 (1.03, 1.49)
	Influenza B virus	1.09 (0.80, 1.47)	1.75 (0.44, 6.98)	0.87 (0.66, 1.15)	1.24 (0.47, 3.29)
SARS-CoV-2 lineage (by variant era)					
	BA.4/BA.5	1.04 (0.96, 1.13)	1.30 (0.91, 1.85)	1.09 (1.01, 1.17)	1.16 (0.85, 1.57)
	XBB/XBB.1.5	1.05 (0.98, 1.13)	1.34 (1.04, 1.73)	0.98 (0.92, 1.04)	1.32 (1.05, 1.65)
	BA.2.86/JN.1	1.01 (0.95, 1.06)	1.26 (0.99, 1.61)	1.06 (1.00, 1.12)	0.97 (0.73, 1.27)

PAS: Post acute sequelae; CI: Confidence interval.

[1]Estimates are computed as adjusted hazards ratios, comparing times to PAS diagnoses among COVID-19 cases versus influenza cases, via doubly-robust Cox proportional hazards models weighted to account for individuals' inverse probability of infection with their identified virus and the inverse of their probability of retention through each 30-day period after the index date. Covariates used in weighting models are included in the analysis model. We use the sandwich variance estimator to account for repeated observations of individuals across multiple 30-day periods.
